# Supplementary material for: Analysis of the Glucose-Dependent Transcriptome in Murine Hypothalamic Cells
Source: Cells. 2022 Feb 11;11(4):639. doi: 10.3390/cells11040639 (PMC8870115; doi:10.3390/cells11040639)
Supplement: Supplementary file 1 [file cells-11-00639-s001.zip › cells-1581535-supplementary.pdf]

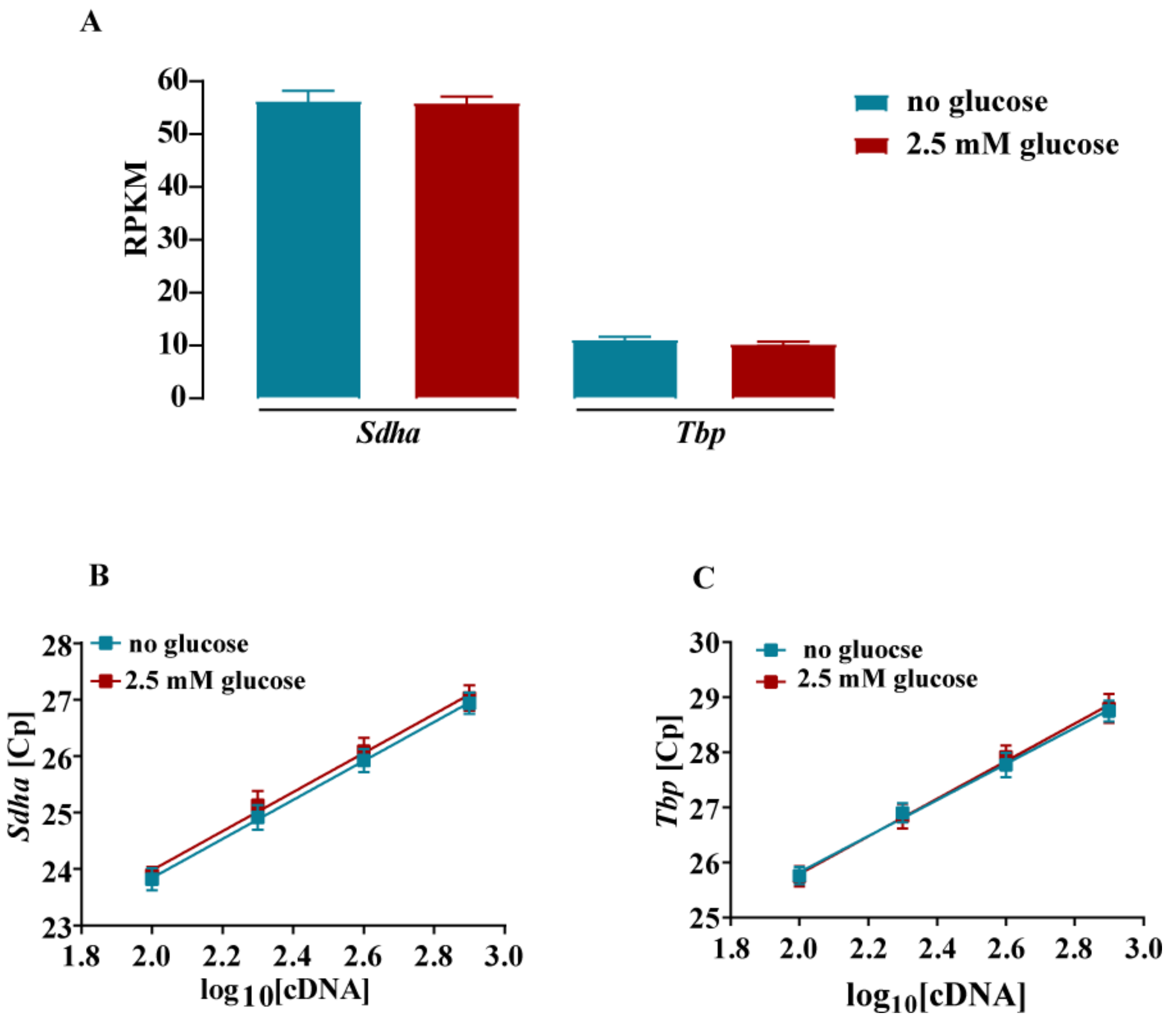

Figure S1. RNA expression of *Sdha* and *Tbp* is resistant against glucose treatment in mHypoA-2/10 cells. In (A), cells were cultured using protocol 1 indicated in section 2.5 and RPKM determined by RNA-seq. In (B and C), cells were cultured using protocol 1 indicated in section 2.5 and mRNA levels determined by qRT-PCR using different cDNA dilutions. In (B) data for *Sdha* and in (C) for *Tbp* of 3 independent (N=3) experiments performed in triplicates are expressed as the mean ±S.E.M

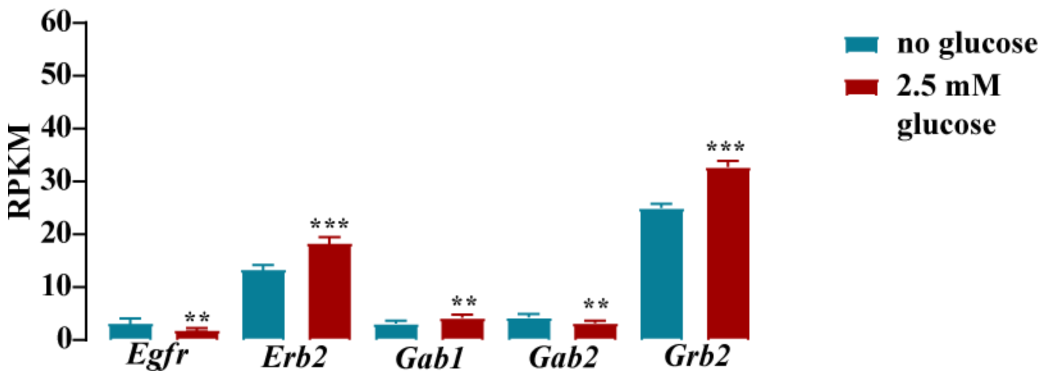

Figure S2. Effects of glucose on mRNA expression of EGF receptors and their adaptor proteins in mHypoA-2/10 cells. Cells were using protocol 1 indicated in section 2.5 and mRNA determined by RNA-seq. Data for the EGF receptor (*Egfr*), for *Erb2,* the Grb2-associated binder-1 (*Gab1*) or -2 (*Gab2*) and for the growth factor receptor-bound protein 2 (*Grb2*) are expressed as the mean ± S.E.M. Asterisks indicate significant differences calculated based on corrected Bonferroni p- values of the entire data set obtained by RNA-seq.

Table S1: List of glucose-dependent genes in mHypoa-2/10 cells with an absolute expression of RPMK > 1

	low gluocse		high glucose					
gene name	MW	STAB	MW	STAB	fold	Pvalue	FDR	GeneID
Sap25	1,27	0,16	8,04	0,98	6,02	0	0	751865
Gas1	2,46	0,25	14,27	1,00	5,64	0	0	14451
Elovl6	5,71	0,21	32,91	0,67	5,58	0	0	170439
Slc39a10	1,93	0,21	10,97	0,97	5,49	0	0	227059
Acat2	41,07	4,13	200,11	1,53	4,71	0	0	110460
Scd2	136,90	2,99	641,61	5,63	4,54	0	0	20250
Mrgprf	1,66	0,11	7,23	0,67	4,24	0	0	211577
Ppp1r10	4,38	0,42	18,82	1,27	4,14	0	0	52040
Pank1	1,44	0,27	5,88	0,42	3,98	0	0	75735
Hmgcs1	48,93	2,19	198,60	2,59	3,93	0	0	208715
Dhcr7	6,32	0,72	25,63	1,56	3,91	0	0	13360
Hsd17b7	7,72	0,49	31,07	0,29	3,89	0	0	15490
C1qtnf1	1,29	0,15	4,95	0,63	3,68	0	0	56745
Fzd2	5,86	0,44	21,65	1,93	3,57	0	0	57265
Pik3r3	2,55	0,37	8,72	0,07	3,30	0	0	18710
Efna2	1,41	0,07	4,71	0,61	3,26	0	0	13637
Cdc42ep2	4,39	0,56	14,84	0,95	3,23	0	0	104252
Gm38481	55,74	1,35	184,37	8,44	3,20	0	0	102635781
Srd5a1	1,15	0,18	3,74	0,60	3,20	0	0	78925
Mvk	16,07	1,76	53,31	3,32	3,19	0	0	17855
Aacs	21,38	1,21	70,42	2,88	3,18	0	0	78894
Crip1	4,82	0,68	15,93	1,02	3,15	0	0	12925
Gm15452	4,87	5,84	15,65	4,76	3,11	0,000371848	0,003243923	100039474
Nr2f1	4,96	0,25	15,50	0,24	3,03	0	0	13865
Gale	2,80	0,20	8,78	0,19	3,03	0	0	74246
Rab7b	1,90	0,09	5,95	0,17	3,02	0	0	226421
Ccnf	8,24	0,42	25,61	0,69	3,00	0	0	12449
Gdf11	2,92	0,20	9,09	0,54	2,99	0	0	14561
Mvd	38,81	4,83	117,17	9,03	2,92	0	0	192156
Efna4	2,90	0,27	8,76	0,24	2,91	0	0	13639
Plk1	6,73	0,63	20,43	1,88	2,91	0	0	18817
Cd248	6,32	1,17	19,09	1,77	2,91	0	0	70445
Idi1	18,59	2,67	55,37	4,77	2,89	0	0	319554
Stard4	21,49	0,46	63,71	1,19	2,87	0	0	170459
Trib2	2,86	0,21	8,32	0,43	2,81	0	0	217410
Hspa1b	9,09	0,77	26,27	1,32	2,81	0	0	15511
Gatsl2	2,67	0,21	7,66	0,69	2,80	0	0	80909
Scd1	62,13	2,26	179,00	1,07	2,79	0	0	20249
Nsdhl	38,81	2,95	111,80	2,05	2,79	0	0	18194
Pif1	1,27	0,22	3,66	0,44	2,77	0	0	208084
Dixdc1	1,93	0,07	5,49	0,10	2,76	0	0	330938
Dhcr24	27,15	1,59	77,24	3,42	2,75	0	0	74754

Gmpr	1,32	0,20	3,70	0,31	2,73	1,42109E-14	4,40325E-13	66355
Plekhg2	1,74	0,17	4,88	0,52	2,72	0	0	101497
Loxl1	12,10	0,58	33,90	3,21	2,71	0	0	16949
Tuba1a	250,79	26,06	696,91	35,57	2,69	0	0	22142
Gm4739	2,70	0,91	7,44	1,51	2,68	1,0023E-10	2,13448E-09	15352
Lss	26,99	1,69	74,83	3,23	2,68	0	0	16987
Mxd3	2,87	0,43	8,12	0,86	2,68	0	0	17121
LOC102637129	50,17	3,48	137,53	5,48	2,66	0	0	102637129
S100a11	131,39	7,81	359,62	25,33	2,65	0	0	20195
Tnfrsf22	5,69	0,58	15,41	0,98	2,63	0	0	79202
Adrb2	2,07	0,11	5,60	0,53	2,63	0	0	11555
Glrx	10,17	0,68	27,56	1,30	2,63	0	0	93692
Polr3g	2,17	0,28	5,87	0,34	2,62	0	0	67486
Rassf2	1,93	0,17	5,16	0,34	2,59	0	0	215653
Zbtb42	1,16	0,20	3,10	0,25	2,58	0	0	382639
Tuba1c	27,13	2,07	72,36	1,20	2,58	0	0	22146
Pcyt1b	3,27	0,17	8,64	0,25	2,57	0	0	236899
Btd	4,51	0,18	12,02	0,83	2,56	0	0	26363
Fam64a	17,70	3,31	46,80	2,68	2,55	0	0	109212
Sqle	67,14	1,84	174,89	1,85	2,52	0	0	20775
Kif22	5,90	0,47	15,45	0,48	2,52	0	0	110033
Gm15772	1,30	0,80	3,37	2,24	2,50	0,006369413	0,042753914	100034726
Ddit4l	3,41	0,20	8,72	1,22	2,50	0	0	73284
Cfp	1,10	0,28	2,82	0,31	2,49	3,04862E-09	5,43938E-08	18636
Mgat5	2,44	0,08	6,25	0,30	2,48	0	0	107895
Acly	75,25	4,09	192,05	8,28	2,47	0	0	104112
Clmp	3,02	0,17	7,68	0,67	2,47	0	0	71566
Dkk3	2,76	0,31	7,05	0,58	2,46	0	0	50781
Crabp2	1,27	0,22	3,21	0,10	2,46	1,4588E-06	1,84921E-05	12904
Dbi	58,20	3,09	148,03	7,07	2,46	0	0	13167
Lpin1	7,71	0,45	19,55	0,49	2,46	0	0	14245
Ppl	4,22	0,05	10,73	1,05	2,45	0	0	19041
Ehd2	17,86	0,99	45,28	0,52	2,45	0	0	259300
S100a4	56,88	0,28	143,63	6,15	2,45	0	0	20198
Pcdhb22	1,24	0,14	3,13	0,11	2,44	4,32987E-15	1,39547E-13	93893
Idh1	29,94	0,87	74,74	3,01	2,42	0	0	15926
Nlgn2	3,69	0,46	9,23	0,67	2,42	0	0	216856
Vegfd	3,18	0,21	7,94	0,02	2,42	0	0	14205
Pcf11	3,24	0,10	8,02	0,37	2,40	0	0	74737
Pfkfb4	1,68	0,06	4,15	0,08	2,40	0	0	270198
Cd24a	47,76	2,80	117,93	10,54	2,40	0	0	12484
Eif4ebp2	17,45	0,89	42,98	1,38	2,38	0	0	13688
Irs1	1,53	0,11	3,72	0,30	2,35	0	0	16367
Tm4sf1	34,46	2,40	83,49	2,25	2,34	0	0	17112
Them6	3,49	0,27	8,37	0,73	2,34	3,33067E-16	1,16357E-14	223626
Cdkn2c	11,17	0,31	26,75	0,81	2,33	0	0	12580

Etnk1	7,28	1,05	17,44	0,15	2,33	0	0	75320
Gpr153	4,60	0,08	11,01	0,91	2,31	0	0	100129
Tgfbr2	14,85	0,42	35,38	1,06	2,30	0	0	21813
Gramd2	2,05	0,16	4,84	0,29	2,29	0	0	546134
Gtse1	20,69	2,05	48,81	2,17	2,28	0	0	29870
Fam180a	2,81	0,38	6,52	0,25	2,27	9,76996E-14	2,79976E-12	208164
Slc39a13	4,56	0,16	10,73	1,29	2,27	0	0	68427
Wdr6	15,37	1,39	36,03	1,72	2,26	0	0	83669
Fscn1	57,39	2,59	134,05	8,04	2,26	0	0	14086
9930012K11Rik	3,57	0,21	8,39	0,70	2,26	0	0	268759
Angptl2	16,01	2,64	37,35	0,96	2,26	0	0	26360
Pmvk	18,53	1,33	43,21	3,39	2,25	0	0	68603
Fasn	35,69	1,06	82,95	13,67	2,25	0	0	14104
Emilin1	9,02	0,28	20,98	0,74	2,25	0	0	100952
Lynx1	3,58	0,05	8,27	0,48	2,25	0	0	23936
Oaf	12,93	0,42	30,05	2,31	2,24	0	0	102644
Plau	4,28	0,35	9,96	0,44	2,24	0	0	18792
Tmem119	7,24	0,48	16,83	1,28	2,24	0	0	231633
S100a13	12,25	1,15	28,43	1,80	2,24	0	0	20196
Trappc1	12,30	0,35	28,22	1,14	2,23	0	0	245828
Tgfb3	3,81	0,34	8,79	0,14	2,22	0	0	21809
0610007P14Rik	17,93	1,13	41,22	0,66	2,22	0	0	58520
Irgq	2,62	0,15	6,01	0,22	2,22	0	0	210146
Scarb1	19,27	1,54	44,20	2,19	2,22	0	0	20778
Ints3	8,57	0,23	19,60	0,74	2,21	0	0	229543
Gm9761	11,86	3,23	27,19	6,29	2,21	2,05341E-11	4,6707E-10	100043705
Peg13	2,51	0,35	5,70	0,28	2,21	0	0	353342
Cdh26	1,04	0,09	2,38	0,12	2,21	6,91869E-12	1,63804E-10	381409
Gpx8	16,66	1,78	37,90	1,49	2,20	0	0	69590
Hmmr	8,53	0,70	19,46	1,08	2,20	0	0	15366
Rnase4	9,40	0,60	21,23	0,53	2,20	0	0	58809
Rdh11	17,74	0,80	40,25	2,13	2,20	0	0	17252
Pxylp1	2,28	0,19	5,18	0,25	2,19	0	0	235534
St3gal5	7,55	0,85	17,05	0,32	2,19	0	0	20454
Msmo1	140,76	5,08	317,02	12,91	2,18	0	0	66234
Pcyt2	21,13	2,40	47,68	2,75	2,18	0	0	68671
Ccne1	4,09	0,45	9,20	0,57	2,18	0	0	12447
Sbk1	1,83	0,11	4,14	0,14	2,17	0	0	104175
Actb	340,55	25,78	763,42	38,02	2,17	0	0	11461
Bcl9l	6,75	0,27	15,15	1,68	2,17	0	0	80288
Dlk2	1,78	0,39	4,01	0,59	2,16	5,38497E-11	1,17466E-09	106565
Gm7331	3,57	1,13	7,97	2,08	2,16	2,88717E-05	0,000303611	654358
Kpna2	58,65	1,18	130,72	2,31	2,16	0	0	16647
Ly6a	1,55	0,24	3,47	0,48	2,16	4,85191E-06	5,74063E-05	110454
Arhgef39	1,43	0,16	3,23	0,65	2,15	1,52659E-09	2,82281E-08	230098
Lurap1l	5,30	0,33	11,69	1,05	2,15	1,11022E-16	3,99131E-15	52829

Card10	3,15	0,20	7,04	0,38	2,15	0	0	105844
Cxcl12	11,00	0,38	24,28	0,53	2,14	0	0	20315
Lmnb1	24,23	0,90	53,19	1,51	2,13	0	0	16906
Nrep	6,36	0,87	13,93	0,74	2,12	0	0	27528
Smim1	1,72	0,15	3,78	0,30	2,12	0	0	68859
Eogt	3,14	0,23	6,79	0,30	2,11	0	0	101351
Adamts5	2,16	0,35	4,71	0,16	2,11	0	0	23794
Insig1	69,23	1,36	150,86	5,36	2,11	0	0	231070
Cyp51	32,12	2,02	70,06	3,60	2,11	0	0	13121
Cenpl	2,62	0,05	5,68	0,42	2,11	0	0	70454
Lfng	3,14	0,27	6,88	0,18	2,11	0	0	16848
Tmem2	3,06	0,04	6,64	0,26	2,11	0	0	83921
Capn5	1,43	0,07	3,10	0,14	2,10	0	0	12337
Ccna2	23,83	0,49	51,63	1,88	2,10	0	0	12428
Acsl3	23,30	0,90	50,47	1,84	2,10	0	0	74205
Abca1	1,10	0,11	2,37	0,19	2,10	0	0	11303
Fkbp14	2,20	0,34	4,76	0,91	2,10	2,32658E-12	5,79935E-11	231997
Nusap1	16,94	1,16	36,74	1,87	2,10	0	0	108907
Midn	14,15	0,35	30,74	1,37	2,10	0	0	59090
Tnfaip8l1	1,77	0,13	3,83	0,37	2,10	3,39695E-11	7,53204E-10	66443
Thbs1	20,42	2,15	44,16	7,22	2,10	0	0	21825
Il15	1,55	0,26	3,30	0,43	2,09	1,0498E-09	1,98039E-08	16168
Kif14	1,35	0,11	2,91	0,11	2,09	0	0	381293
Kif20a	25,96	2,21	55,78	2,06	2,08	0	0	19348
Rap2a	6,50	0,53	13,85	0,80	2,08	0	0	76108
Pnp	21,67	0,62	46,39	1,35	2,07	0	0	18950
Aldh3a1	1,27	0,16	2,74	0,50	2,07	9,86307E-07	1,27943E-05	11670
Skp2	6,33	0,41	13,53	0,78	2,06	0	0	27401
Tnfrsf23	4,54	0,26	9,65	0,86	2,06	0	0	79201
Arhgef19	3,47	0,23	7,41	0,73	2,06	0	0	213649
Pgp	16,76	1,56	35,81	1,36	2,05	0	0	67078
Cdkn2d	7,32	0,49	15,51	0,54	2,05	0	0	12581
Ogn	12,70	1,03	26,91	0,14	2,05	0	0	18295
Azin2	2,09	0,11	4,44	0,32	2,05	0	0	242669
Msl1	7,35	0,20	15,44	0,44	2,04	0	0	74026
Cbx3-ps6	1,34	0,68	2,82	0,79	2,04	0,000251986	0,002268767	629578
Amd1	20,26	0,72	42,47	3,18	2,04	0	0	11702
Gstm1	31,65	1,56	66,64	2,71	2,03	0	0	14862
Lrp8	5,16	0,07	10,85	0,79	2,03	0	0	16975
Zfp579	2,06	0,15	4,37	0,79	2,03	7,83373E-13	2,04863E-11	68490
Srf	4,82	0,30	10,12	0,85	2,03	0	0	20807
C1galt1c1	4,96	0,39	10,35	1,52	2,03	1,33782E-13	3,78635E-12	59048
Fam83d	6,41	0,73	13,55	0,25	2,03	0	0	71878
Hyal2	5,40	0,20	11,39	0,71	2,03	0	0	15587
Ldlr	35,82	4,44	74,95	5,94	2,03	0	0	16835
Hn1	47,57	5,89	98,90	0,89	2,01	0	0	15374

Hspa1l	1,19	0,05	2,47	0,12	2,01	4,05093E-08	6,34682E-07	15482
2300009A05Rik	1,46	0,24	3,05	0,38	2,01	0,001905747	0,014418336	69478
Cmc4	1,61	0,40	3,41	0,36	2,01	2,84525E-05	0,000299754	105886298
Pitpnc1	1,12	0,15	2,36	0,09	2,01	0	0	71795
Lpcat3	17,45	0,45	36,13	0,62	2,01	0	0	14792
Apln	3,08	0,40	6,32	0,24	2,00	1,63203E-14	5,02623E-13	30878
Mis18bp1	3,06	0,15	6,36	0,37	2,00	0	0	217653
Med29	2,14	0,63	4,44	0,37	1,99	9,59269E-09	1,62056E-07	67224
Pip4k2b	14,41	0,21	29,64	0,56	1,99	0	0	108083
Ttyh3	7,00	0,16	14,42	0,30	1,99	0	0	78339
Celf5	1,11	0,20	2,25	0,07	1,99	1,36124E-12	3,46481E-11	319586
Mcee	2,99	0,55	6,19	1,05	1,99	7,34246E-07	9,6838E-06	73724
Kank2	18,09	1,06	37,12	2,00	1,99	0	0	235041
Jup	9,26	0,55	19,00	0,94	1,99	0	0	16480
Rgma	4,97	0,62	10,25	0,65	1,98	0	0	244058
Stxbp6	1,14	0,02	2,33	0,09	1,98	9,62789E-11	2,05513E-09	217517
Adgrl1	3,46	0,25	7,08	0,48	1,98	0	0	330814
Pml	5,43	0,13	11,10	0,35	1,98	0	0	18854
Fam212b	3,33	0,18	6,75	0,38	1,98	0	0	109050
Srl	1,41	0,02	2,87	0,07	1,97	0	0	106393
Rasl12	1,06	0,10	2,16	0,18	1,97	4,7093E-10	9,24984E-09	70784
Mtcp1	1,00	0,21	2,02	0,08	1,97	3,57571E-05	0,000369729	17763
Pidd1	2,60	0,24	5,32	0,17	1,97	0	0	57913
Txnip	5,05	0,26	10,26	0,52	1,97	0	0	56338
Mrps21	26,98	0,11	55,06	5,40	1,96	0	0	66292
Atp8b2	14,47	0,33	29,36	0,93	1,96	0	0	54667
Nav1	2,07	0,14	4,18	0,47	1,96	0	0	215690
Mettl7a1	2,71	0,18	5,44	0,67	1,96	5,35305E-12	1,2847E-10	70152
Gm9385	7,46	3,76	15,22	7,19	1,96	0,000928792	0,007512574	668829
Npcd	1,98	0,28	4,03	0,45	1,95	1,73417E-13	4,82751E-12	504193
Zfp385a	10,10	0,89	20,35	1,12	1,95	0	0	29813
Cped1	3,88	0,10	7,76	0,54	1,94	0	0	214642
Cd9	98,22	4,06	196,83	7,80	1,94	0	0	12527
Rcor2	2,36	0,24	4,76	0,26	1,94	1,44329E-15	4,84253E-14	104383
Ccnd1	19,09	1,40	38,43	1,22	1,94	0	0	12443
Slc35a4	24,52	0,98	49,09	0,24	1,94	0	0	67843
Gm2573	1,02	0,60	2,06	0,94	1,94	0,019574957	0,114911222	100040052
Fam26e	2,34	0,41	4,71	0,18	1,93	4,78681E-09	8,37098E-08	103511
Sesn1	8,28	0,39	16,53	0,54	1,93	0	0	140742
Snca	6,83	0,14	13,63	1,19	1,93	0	0	20617
Aurkb	11,22	0,65	22,52	0,97	1,93	0	0	20877
Dnajb1	13,66	0,45	27,11	1,43	1,93	0	0	81489
Col3a1	11,36	0,71	22,75	1,07	1,93	0	0	12825
Gnao1	1,94	0,11	3,87	0,16	1,93	0	0	14681
Ptrf	63,73	2,83	127,05	4,06	1,93	0	0	19285
4930579G24Rik	7,57	0,27	14,96	1,53	1,93	0	0	75939

Dmpk	2,67	0,37	5,39	0,17	1,93	2,22045E-16	7,82284E-15	13400
Sgk1	17,10	0,89	34,10	1,94	1,93	0	0	20393
Gm10774	21,17	1,88	42,62	5,67	1,93	3,39695E-12	8,34468E-11	100043714
Tef	8,02	0,30	15,93	0,23	1,93	0	0	21685
Aurka	11,78	1,43	23,56	1,28	1,93	0	0	20878
Ctnnal1	9,26	0,36	18,33	0,22	1,92	0	0	54366
Sertad3	2,36	0,51	4,69	0,28	1,92	2,49568E-07	3,52297E-06	170742
Ano8	4,32	0,23	8,52	0,18	1,92	0	0	382014
Fam171a2	3,44	0,17	6,84	0,18	1,92	0	0	217219
Patz1	6,03	0,34	11,91	0,38	1,92	0	0	56218
Hspb8	26,90	0,96	53,30	0,75	1,92	0	0	80888
Mttp	1,14	0,12	2,24	0,14	1,92	8,45615E-10	1,61991E-08	17777
Zfp52	2,96	0,40	5,85	0,33	1,92	0	0	22710
Chst12	9,68	0,24	19,13	0,52	1,92	0	0	59031
Mrpl18	23,44	0,24	46,24	2,87	1,91	0	0	67681
Ifitm2	62,00	6,14	122,66	5,66	1,91	0	0	80876
Rrm2	33,09	1,96	65,35	4,24	1,91	0	0	20135
Prc1	18,63	1,91	36,85	1,12	1,91	0	0	233406
Hmgb3	11,90	0,60	23,35	1,94	1,91	0	0	15354
Kcnf1	1,67	0,28	3,28	0,64	1,90	1,01954E-11	2,38666E-10	382571
Pbx3	2,41	0,19	4,72	0,03	1,90	1,11022E-16	3,99131E-15	18516
Mat2a	60,55	1,47	118,84	7,03	1,90	0	0	232087
Hacl1	2,19	0,50	4,23	0,20	1,90	5,00361E-10	9,78172E-09	56794
Utp14b	3,37	0,14	6,66	0,35	1,90	0	0	195434
Nop10	18,06	0,78	35,54	1,15	1,90	0	0	66181
Nxpe4	1,04	0,21	2,04	0,11	1,90	1,56097E-08	2,57805E-07	244853
Large	4,91	0,22	9,59	0,41	1,89	0	0	16795
Dynll1	40,90	2,76	79,67	4,43	1,89	0	0	56455
Tcf7l1	5,92	0,43	11,62	0,40	1,89	0	0	21415
Mrpl34	13,38	0,82	26,03	2,80	1,89	2,22045E-16	7,82284E-15	94065
Dffa	2,24	0,26	4,31	0,25	1,89	5,00061E-10	9,78005E-09	13347
Tmsb4x	115,97	6,64	225,77	6,50	1,89	0	0	19241
B3gnt3	2,47	0,24	4,76	0,54	1,88	2,48707E-10	5,0587E-09	72297
Dna2	2,49	0,09	4,82	0,25	1,88	0	0	327762
Cks2	23,41	4,38	45,38	2,69	1,88	0	0	66197
Cdca3	17,35	0,59	33,68	2,42	1,87	0	0	14793
Map3k8	1,40	0,16	2,70	0,14	1,87	2,58933E-08	4,17108E-07	26410
Frmd8	16,08	0,97	31,11	0,96	1,87	0	0	67457
Pgd	42,68	1,72	82,41	1,00	1,87	0	0	110208
Rpl36-ps2	2,11	0,64	4,13	2,72	1,87	0,0365476	0,195766737	100043483
Gm6788	9,63	3,74	18,55	7,70	1,87	0,00022212	0,002016928	627788
St6gal1	1,87	0,16	3,57	0,34	1,86	3,97582E-12	9,69384E-11	20440
Tbc1d4	1,28	0,10	2,42	0,21	1,86	2,01927E-12	5,0609E-11	210789
Sc5d	30,82	1,01	59,23	0,84	1,86	0	0	235293
Men1	17,09	1,08	32,78	1,16	1,86	0	0	17283
Tmem79	1,73	0,08	3,29	0,59	1,86	8,0544E-08	1,21333E-06	71913

Synpo	3,05	0,41	5,88	0,29	1,86	0	0	104027
Tfap2b	5,99	0,24	11,49	0,22	1,86	0	0	21419
Adamts10	2,87	0,46	5,54	0,77	1,86	2,42917E-13	6,645E-12	224697
Prrx2	10,82	1,49	20,85	0,66	1,85	0	0	20204
Rab6b	1,87	0,03	3,57	0,19	1,85	0	0	270192
Acss2	4,93	0,86	9,50	0,60	1,85	0	0	60525
Chst1	7,48	0,92	14,32	0,42	1,85	0	0	76969
Acaca	11,16	0,12	21,34	1,64	1,85	0	0	107476
Fabp5	59,06	2,61	112,73	3,14	1,85	0	0	16592
Ptgs2os2	1,25	0,15	2,38	0,11	1,85	4,55191E-14	1,34573E-12	102639566
Cdk1	30,73	2,44	58,51	2,92	1,84	0	0	12534
Mbd5	1,15	0,08	2,19	0,13	1,84	0	0	109241
Tcaf1	6,87	0,40	12,99	0,34	1,84	0	0	77574
Prcc	10,77	1,08	20,57	0,83	1,84	0	0	94315
Hmgcr	30,64	0,88	58,13	1,75	1,84	0	0	15357
Asap3	2,38	0,13	4,54	0,39	1,83	0	0	230837
Magee1	5,20	0,41	9,87	0,22	1,83	0	0	107528
Troap	9,42	1,16	17,93	1,45	1,83	0	0	78733
Bmp4	6,76	0,35	12,86	0,70	1,83	0	0	12159
Cisd3	6,86	0,65	13,03	0,52	1,83	1,58762E-14	4,89275E-13	217149
Spc25	10,36	1,19	19,58	2,17	1,83	0	0	66442
4933412E12Rik	1,36	0,07	2,57	0,16	1,82	0	0	71086
Sema3f	1,67	0,19	3,16	0,29	1,82	2,02218E-10	4,15745E-09	20350
Pcyox1l	3,95	0,26	7,42	0,44	1,82	1,11022E-16	3,99131E-15	240334
6330408A02Rik	1,68	0,24	3,13	0,22	1,82	8,97325E-09	1,52153E-07	321008
Rnaseh2c	14,49	1,83	27,47	3,42	1,82	6,76349E-11	1,46281E-09	68209
Fdps	163,57	8,53	307,25	5,36	1,82	0	0	110196
Dynlt1c	59,12	1,09	110,91	11,71	1,82	0	0	100040563
Slc27a3	3,32	0,20	6,23	0,25	1,82	1,22125E-15	4,12779E-14	26568
Tubb4b	114,30	12,58	214,49	5,31	1,82	0	0	227613
Cic	9,09	0,62	17,09	1,23	1,82	0	0	71722
Crip2	42,52	4,09	79,84	5,65	1,82	0	0	68337
Pgpep1	9,62	0,25	18,01	0,20	1,81	0	0	66522
Mknk2	42,00	3,17	78,72	0,60	1,81	0	0	17347
Suv420h2	2,50	0,27	4,69	0,65	1,81	8,38841E-11	1,80233E-09	232811
Fam110a	3,33	0,52	6,26	0,71	1,81	5,10288E-11	1,11472E-09	73847
Pbx1	10,22	0,27	19,11	0,52	1,81	0	0	18514
Cd81	56,47	0,66	105,57	2,37	1,81	0	0	12520
Abhd8	11,53	0,33	21,66	1,21	1,81	0	0	64296
Ly6e	15,22	2,05	28,57	0,97	1,81	0	0	17069
Polr2l	2,14	0,31	4,01	0,78	1,81	1,04464E-05	0,000117435	66491
Cdc20	23,01	2,96	43,06	3,24	1,81	0	0	107995
Mmab	9,63	0,34	17,97	0,15	1,81	0	0	77697
Rbbp6	6,79	0,65	12,63	0,53	1,80	0	0	19647
Tuba1b	202,76	17,23	377,77	11,64	1,80	0	0	22143
Abcd1	2,66	0,11	4,95	0,17	1,80	3,21965E-15	1,04576E-13	11666

Bok	8,40	1,40	15,72	1,23	1,80	6,7476E-12	1,60002E-10	51800
Adamts4	2,96	0,29	5,55	0,67	1,80	3,60251E-11	7,97238E-10	240913
Lenep	1,16	0,47	2,13	0,13	1,80	0,018487179	0,109423921	57275
Hrct1	1,02	0,32	1,97	0,19	1,80	0,007727461	0,050846532	100039781
Dnajb4	6,50	0,28	12,03	1,50	1,80	0	0	67035
Kif2c	14,04	2,11	26,16	1,15	1,80	0	0	73804
Espl1	6,77	0,46	12,62	1,29	1,80	0	0	105988
S100a1	20,00	1,39	37,36	3,60	1,80	0	0	20193
Tlcd1	2,37	0,12	4,40	0,29	1,80	1,00697E-13	2,87666E-12	68385
Sigmar1	10,31	0,42	19,16	0,44	1,80	0	0	18391
Top2a	49,80	1,74	92,35	3,02	1,80	0	0	21973
Phb	21,70	1,52	40,02	1,61	1,79	0	0	18673
Bak1	21,88	1,26	40,59	1,82	1,79	0	0	12018
Car5b	6,33	0,13	11,67	0,70	1,79	0	0	56078
Ell2	6,83	0,49	12,59	1,06	1,79	0	0	192657
E2f8	3,21	0,07	5,89	0,61	1,78	0	0	108961
Cyb5b	58,63	2,19	108,02	2,57	1,78	0	0	66427
Cd14	4,03	0,71	7,42	0,30	1,78	5,74186E-09	9,94988E-08	12475
Arhgef17	6,47	0,48	11,92	1,50	1,78	0	0	207212
Def6	4,25	0,48	7,91	0,54	1,78	1,13243E-14	3,54242E-13	23853
Alg14	4,62	0,26	8,51	0,23	1,78	1,03251E-14	3,2387E-13	66789
Dnd1	1,02	0,06	1,89	0,63	1,78	0,004044562	0,028516312	213236
Ank	46,53	2,74	85,36	1,76	1,78	0	0	11732
Galnt7	5,12	0,49	9,31	0,45	1,77	0	0	108150
Camta2	2,34	0,28	4,27	0,25	1,77	2,22045E-16	7,82284E-15	216874
Kif11	17,56	0,82	32,16	0,75	1,77	0	0	16551
Mylip	5,00	0,80	9,16	0,52	1,77	0	0	218203
Mesdc1	3,49	0,09	6,40	0,24	1,77	0	0	80889
S100a6	1011,41	20,71	1845,27	44,36	1,77	0	0	20200
Prkag2	3,69	0,27	6,70	0,07	1,77	0	0	108099
Tpcn1	8,13	0,47	14,88	0,76	1,76	0	0	252972
Ppil1	29,37	1,34	53,40	4,83	1,76	0	0	68816
Kcnk5	1,09	0,12	1,99	0,15	1,76	2,58626E-07	3,6441E-06	16529
Agfg2	13,01	1,19	23,71	1,53	1,76	0	0	231801
Mmgt2	3,36	0,20	6,06	0,36	1,76	7,02438E-13	1,84435E-11	216829
Hspa1a	1,15	0,55	2,09	0,75	1,76	0,004450035	0,031064039	193740
Prr11	6,59	0,68	12,00	0,13	1,75	0	0	270906
Gm42047	10,71	0,62	19,40	1,50	1,75	0	0	105246823
Cit	1,43	0,07	2,59	0,28	1,75	0	0	12704
Angpt1	1,04	0,16	1,89	0,09	1,75	3,89485E-07	5,33516E-06	11600
Tmem198b	5,38	0,50	9,77	0,21	1,75	0	0	73827
Nectin1	2,02	0,09	3,65	0,19	1,75	3,4972E-14	1,04676E-12	58235
Esco2	5,74	0,21	10,37	0,65	1,75	0	0	71988
Fbxl19	2,32	0,16	4,18	0,16	1,75	2,22045E-16	7,82284E-15	233902
Luc7l3	17,68	0,64	31,84	3,35	1,75	0	0	67684
Uqcrh	70,44	2,70	126,62	9,01	1,75	0	0	66576

Cryab	45,83	3,28	82,79	4,19	1,74	0	0	12955
Kif20b	11,93	0,40	21,51	0,30	1,74	0	0	240641
Mblac2	1,52	0,11	2,73	0,03	1,74	4,2149E-10	8,33239E-09	72852
Nfatc4	3,23	0,28	5,85	0,21	1,74	6,66134E-16	2,2956E-14	73181
Myl9	18,43	1,57	33,33	1,07	1,74	0	0	98932
Rpl37rt	10,81	4,44	19,58	6,87	1,74	0,000493673	0,004216674	100502825
Rps27rt	18,59	1,65	33,64	8,68	1,74	6,27199E-07	8,35383E-06	100043813
Dnajc12	2,01	0,17	3,54	0,77	1,74	0,000114008	0,00109063	30045
Scara3	6,01	0,34	10,81	0,67	1,74	0	0	219151
Irgm1	24,77	1,64	44,68	0,91	1,74	0	0	15944
Furin	8,11	0,80	14,61	0,87	1,74	0	0	18550
Ebp	28,14	1,18	50,44	3,32	1,74	0	0	13595
Ube2c	44,20	3,67	79,73	4,59	1,74	0	0	68612
Pttg1	6,12	0,10	11,09	1,33	1,74	6,36404E-10	1,23098E-08	30939
Marcksl1	49,84	1,51	89,39	1,91	1,74	0	0	17357
Nuf2	12,77	1,17	22,87	0,81	1,74	0	0	66977
Tpmt	1,79	0,28	3,17	0,38	1,73	3,82949E-08	6,01842E-07	22017
Tmem19	12,82	0,44	22,83	1,12	1,73	0	0	67226
Ttc21b	3,45	0,14	6,10	0,48	1,73	0	0	73668
Pcdhb17	2,21	0,15	3,97	0,25	1,73	3,12366E-11	6,95979E-10	93888
Gm7730	1,12	0,45	2,08	1,05	1,73	0,070654894	0,337035775	665649
Farp2	3,43	0,34	6,11	0,23	1,73	9,99201E-16	3,40241E-14	227377
Hdhd3	2,25	0,40	4,01	0,23	1,73	6,79385E-05	0,000674448	72748
Rhof	1,08	0,17	1,90	0,33	1,73	0,000259147	0,002325009	23912
Kcnd1	2,19	0,18	3,90	0,10	1,73	4,05764E-12	9,86706E-11	16506
Gm14137	1,25	0,13	2,19	0,26	1,73	5,35837E-06	6,30238E-05	623781
Rnf145	8,36	0,18	14,91	0,37	1,73	0	0	74315
Hsph1	24,08	0,71	42,82	3,83	1,72	0	0	15505
Dynlt1f	3,91	0,71	7,01	0,85	1,72	6,19682E-10	1,20168E-08	100040531
Casp6	5,18	0,20	9,16	0,55	1,72	2,22107E-11	5,03204E-10	12368
BC028528	1,00	0,19	1,79	0,08	1,72	0,001111692	0,008855799	229600
Nacc2	2,92	0,33	5,15	0,45	1,72	0	0	67991
Runx3	1,08	0,15	1,93	0,24	1,72	1,26799E-06	1,61868E-05	12399
Ccnb1	26,78	1,80	47,55	1,39	1,72	0	0	268697
Aif1l	12,29	0,90	21,75	0,52	1,72	0	0	108897
Eef1akmt1	11,34	0,56	19,93	1,02	1,72	1,95177E-13	5,41023E-12	68043
Tspan13	1,18	0,11	2,10	0,14	1,72	0,000134875	0,001273477	66109
Syk	3,19	0,26	5,65	0,14	1,72	0	0	20963
Slc9a3r1	7,09	0,22	12,46	0,97	1,71	2,33147E-15	7,69284E-14	26941
Cacna1a	1,36	0,13	2,38	0,08	1,71	4,77396E-15	1,53212E-13	12286
Sapcd2	1,83	0,09	3,27	0,25	1,71	3,75139E-10	7,46121E-09	72080
Zfp512b	4,81	0,22	8,52	0,33	1,71	0	0	269401
1190002N15Rik	3,96	0,55	6,95	0,59	1,71	3,33067E-16	1,16357E-14	68861
Dexi	1,39	0,15	2,48	0,20	1,71	1,47499E-10	3,07952E-09	58239
Mospd3	5,41	1,28	9,61	0,15	1,71	5,41551E-09	9,40216E-08	68929
6720489N17Rik	1,86	0,22	3,26	0,34	1,71	2,84104E-07	3,97615E-06	211378

Mis18a	8,25	0,79	14,35	1,32	1,71	2,62061E-09	4,73105E-08	66578
Ccng1	70,84	3,85	124,73	13,18	1,71	0	0	12450
Tmem37	5,06	0,42	9,00	0,55	1,70	6,52171E-07	8,66374E-06	170706
Cdk2	24,74	0,35	43,53	1,10	1,70	0	0	12566
Ccdc28b	2,42	0,24	4,25	0,12	1,70	0,000311097	0,002756506	66264
E2f7	6,65	0,54	11,73	0,40	1,70	0	0	52679
Echdc1	4,19	0,21	7,34	1,08	1,70	0	0	52665
Plpp1	12,90	1,17	22,61	1,00	1,70	0	0	19012
Hexim1	13,28	1,02	23,25	0,28	1,70	0	0	192231
Nuak2	3,53	0,23	6,17	0,37	1,70	7,37188E-14	2,13528E-12	74137
Mrps6	46,86	0,84	81,84	5,48	1,70	0	0	121022
Cdc42ep4	6,40	0,14	11,23	0,42	1,69	0	0	56699
Dad1	6,38	0,35	11,09	0,99	1,69	0	0	13135
LOC108167942	2,01	0,02	3,53	0,28	1,69	4,48342E-07	6,10484E-06	108167942
Gm12715	2,19	1,05	3,85	1,62	1,69	0,011731917	0,073599885	100039940
Fam57a	7,73	0,24	13,44	0,91	1,69	3,33067E-16	1,16357E-14	116972
Ephb3	1,54	0,22	2,71	0,56	1,69	4,74598E-06	5,62403E-05	13845
Actg1	483,33	38,61	844,68	40,98	1,69	0	0	11465
Tm7sf2	13,59	1,29	23,87	3,89	1,69	7,87084E-11	1,6943E-09	73166
Acadm	10,31	0,58	17,84	1,44	1,69	0	0	11364
St3gal2	14,23	0,45	24,83	1,18	1,69	0	0	20444
Ssc5d	2,36	0,16	4,14	0,56	1,69	2,70211E-09	4,85902E-08	269855
Klhl26	14,69	0,61	25,59	0,55	1,69	0	0	234378
Medag	10,18	0,86	17,81	0,74	1,69	0	0	70717
H3f3a-ps1	1,81	0,50	3,13	0,34	1,69	0,004209877	0,029599785	15079
Amot	3,39	0,23	5,87	0,06	1,68	0	0	27494
Tnfsfm13	1,75	0,23	3,06	0,69	1,68	0,000169072	0,001570129	619441
Nanos1	1,89	0,18	3,26	0,39	1,68	9,23564E-08	1,38172E-06	332397
Bnc2	1,28	0,07	2,22	0,29	1,68	2,22045E-16	7,82284E-15	242509
Lasp1	46,89	2,42	81,57	4,11	1,68	0	0	16796
Trappc13	7,65	0,61	13,28	1,39	1,68	0	0	66975
Cdca8	18,98	1,35	33,10	0,80	1,68	0	0	52276
Adamts7	3,27	0,33	5,72	0,23	1,68	1,11022E-16	3,99131E-15	108153
Chml	3,03	0,23	5,21	0,40	1,68	2,22045E-16	7,82284E-15	12663
Fam111a	39,63	1,11	68,63	4,03	1,68	0	0	107373
Dars2	2,37	0,28	4,11	0,36	1,68	3,03496E-11	6,77206E-10	226539
Mob3a	14,63	1,19	25,48	1,38	1,68	0	0	208228
Nacc1	26,51	0,87	45,99	2,05	1,68	0	0	66830
Apcdd1	2,76	0,09	4,79	0,48	1,68	1,91186E-10	3,93594E-09	494504
Nfic	17,54	0,66	30,38	2,29	1,68	0	0	18029
Fam189b	8,98	0,32	15,58	0,26	1,68	0	0	68521
Cox7b	45,70	2,65	78,72	5,00	1,68	0	0	66142
Kbtbd4	6,33	0,25	10,89	0,80	1,67	1,9984E-15	6,64175E-14	67136
1110012L19Rik	15,58	1,28	26,69	1,59	1,67	2,22045E-15	7,3318E-14	68618
Megf8	2,90	0,13	5,00	0,25	1,67	0	0	269878
Casp9	5,32	0,39	9,18	0,64	1,67	0	0	12371

LOC102638150	1,88	0,07	3,28	0,22	1,67	0,0102143	0,065226958	102638150
LOC102640133	2,33	0,73	3,96	0,86	1,67	0,000703608	0,005830274	102640133
Triobp	10,25	0,37	17,72	0,95	1,67	0	0	110253
Gm13192	1,32	0,79	2,26	1,89	1,67	0,212658436	0,748459734	100040426
LOC108167452	2,19	0,17	3,77	0,06	1,67	5,79536E-14	1,69256E-12	108167452
Cdca5	11,02	0,35	18,95	0,93	1,67	1,11022E-16	3,99131E-15	67849
Lrch4	10,56	1,18	18,26	1,29	1,67	0	0	231798
B4galt6	5,85	0,55	10,07	0,67	1,67	0	0	56386
S1pr1	5,34	0,46	9,29	0,90	1,67	4,55191E-15	1,46394E-13	13609
2810417H13Rik	19,06	0,38	32,66	1,74	1,66	0	0	68026
Ephb4	4,71	0,54	8,11	0,35	1,66	1,11022E-16	3,99131E-15	13846
Tle6	2,29	0,29	3,92	0,11	1,66	1,03949E-06	1,34233E-05	114606
Igip	1,95	0,62	3,29	0,19	1,66	0,000551272	0,004663394	109169
Fut11	3,48	0,27	5,93	0,54	1,66	9,45076E-09	1,59776E-07	73068
Rgmb	3,73	0,52	6,38	0,46	1,66	2,22045E-16	7,82284E-15	68799
Aspm	4,45	0,11	7,64	0,90	1,66	0	0	12316
Slc39a8	1,21	0,24	2,04	0,27	1,66	1,56204E-05	0,000171349	67547
Gm6166	68,45	5,72	117,46	6,19	1,66	0	0	101056162
Ints8	6,69	0,17	11,42	1,47	1,66	0	0	72656
Fads2	77,27	6,35	132,47	5,24	1,66	0	0	56473
Ankrd9	2,26	0,37	3,91	0,24	1,66	7,99608E-07	1,05035E-05	74251
Abcb10	11,66	0,41	19,96	0,42	1,66	0	0	56199
Gm6043	1,12	0,21	1,85	0,47	1,66	0,042936731	0,224321685	595138
Bad	10,02	0,61	17,18	1,06	1,66	2,65343E-14	8,01547E-13	12015
Trex1	6,67	1,04	11,53	0,57	1,66	1,885E-07	2,71878E-06	22040
Prdx1	184,99	8,76	315,92	17,17	1,66	0	0	18477
Ddah2	15,18	1,51	26,04	1,32	1,66	0	0	51793
Kif18b	9,00	0,97	15,43	1,02	1,66	0	0	70218
Ncaph	14,99	0,63	25,69	0,89	1,65	0	0	215387
Clic1	167,42	10,11	286,22	6,23	1,65	0	0	114584
Cenpf	5,07	0,25	8,67	0,75	1,65	0	0	108000
Trim12c	2,10	0,26	3,58	0,42	1,65	1,94467E-07	2,79779E-06	319236
Gsn	74,25	6,28	126,60	10,60	1,65	0	0	227753
Rasl11b	2,49	0,42	4,28	0,15	1,65	8,63534E-06	9,82831E-05	68939
Tor4a	3,26	0,15	5,58	0,20	1,65	4,49329E-12	1,08687E-10	227612
Tsc22d4	16,88	1,05	28,86	1,11	1,65	0	0	78829
Cstf3	4,72	0,18	8,03	0,33	1,65	0	0	228410
2810006K23Rik	2,64	0,20	4,43	0,48	1,65	2,80487E-07	3,93274E-06	72650
Atp5g3	101,87	2,54	172,95	4,86	1,65	0	0	228033
Lox	15,28	0,81	25,96	2,71	1,65	0	0	16948
Ivd	2,95	0,30	4,99	0,65	1,64	3,03721E-08	4,84313E-07	56357
Hoxb7	1,12	0,18	1,92	0,07	1,64	0,006122162	0,041257659	15415
Taok2	5,81	0,26	9,90	0,75	1,64	0	0	381921
Cdca2	3,29	0,23	5,60	0,15	1,64	0	0	108912
Sdhd	38,59	0,96	65,39	1,07	1,64	0	0	66925
Rab3d	3,35	0,30	5,68	0,40	1,64	3,87801E-13	1,04398E-11	19340

Pald1	4,02	0,18	6,84	0,14	1,64	5,77316E-15	1,84761E-13	27355
Fam63b	2,28	0,20	3,83	0,24	1,64	2,55351E-15	8,39522E-14	235461
Sipa1	11,94	0,96	20,28	1,39	1,64	0	0	20469
Jmjd4	2,76	0,16	4,67	0,59	1,64	1,61996E-08	2,67163E-07	194952
Pank3	15,23	1,63	25,75	1,25	1,64	0	0	211347
Tmtc3	10,61	0,48	17,91	1,28	1,64	0	0	237500
Ckap2	30,98	0,27	52,28	2,55	1,64	0	0	80986
Zfp219	2,31	0,04	3,93	0,32	1,64	3,44169E-15	1,11235E-13	69890
2810403A07Rik	12,08	1,39	20,30	0,97	1,64	0	0	74200
Nat6	3,77	0,18	6,37	0,20	1,63	2,17243E-09	3,9547E-08	56441
Ost4	43,11	4,36	72,91	1,46	1,63	0	0	67695
Rps6ka1	3,31	0,06	5,53	0,61	1,63	2,63693E-08	4,23881E-07	20111
Akap8	15,48	0,91	26,06	1,41	1,63	0	0	56399
Cav1	81,86	2,70	138,08	3,28	1,63	0	0	12389
Parvb	6,79	0,54	11,49	0,41	1,63	0	0	170736
Map3k6	1,79	0,30	3,05	0,18	1,63	8,17645E-08	1,2305E-06	53608
Ctxn1	13,63	1,35	23,00	0,52	1,63	3,9968E-14	1,18852E-12	330695
Cxxc5	4,83	0,07	8,12	0,39	1,63	6,43929E-15	2,05362E-13	67393
Man1a2	8,81	0,57	14,78	0,76	1,63	0	0	17156
Myl6	466,56	32,17	785,83	38,86	1,63	0	0	17904
Fhod1	7,90	0,78	13,34	1,12	1,63	0	0	234686
Smad6	1,90	0,26	3,23	0,25	1,63	3,02786E-06	3,68832E-05	17130
Depdc5	2,97	0,26	4,98	0,10	1,63	1,11022E-16	3,99131E-15	277854
Cenpe	8,52	0,41	14,36	0,96	1,63	0	0	229841
Zmynd8	6,09	0,28	10,24	0,47	1,63	0	0	228880
Samd1	17,54	0,29	29,44	1,06	1,63	0	0	666704
B3gat3	10,94	0,35	18,30	1,22	1,63	3,33067E-14	9,99528E-13	72727
Cnpy4	15,18	0,86	25,46	2,03	1,63	1,11022E-16	3,99131E-15	66455
Gpr137	2,27	0,08	3,84	0,34	1,63	7,67016E-09	1,31273E-07	107173
Pdgfa	6,45	0,24	10,82	0,29	1,63	1,94289E-14	5,94758E-13	18590
Ccdc80	10,36	0,26	17,43	1,61	1,63	0	0	67896
Grik5	8,47	0,47	14,22	1,23	1,63	0	0	14809
Tmem164	7,00	0,35	11,76	0,28	1,62	0	0	209497
Cbx6	26,96	0,29	45,19	1,13	1,62	0	0	494448
Dennd3	1,63	0,20	2,71	0,15	1,62	7,43423E-10	1,42893E-08	105841
2310022B05Rik	30,78	1,46	51,56	1,41	1,62	0	0	69551
Nfix	21,14	0,62	35,44	0,74	1,62	0	0	18032
Uqcr11	77,81	8,70	130,66	10,15	1,62	0	0	66594
Ccnd3	19,88	1,51	33,41	0,88	1,62	0	0	12445
Ei24	34,17	1,31	57,13	3,84	1,62	0	0	13663
Tmem260	1,10	0,10	1,83	0,12	1,62	1,05241E-06	1,35787E-05	218989
G6pdx	32,78	0,51	54,90	1,98	1,62	0	0	14381
5730408K05Rik	1,86	0,25	3,11	0,30	1,62	0,061256065	0,300405469	67531
Apc2	2,78	0,14	4,66	0,56	1,62	0	0	23805
Gstk1	1,65	0,24	2,74	0,68	1,62	0,010558245	0,067166815	76263
Ttk	7,35	0,14	12,31	0,28	1,62	8,88178E-16	3,03566E-14	22137

Cacna2d1	1,65	0,13	2,76	0,08	1,62	2,59792E-14	7,86856E-13	12293
Anxa8	21,14	2,24	35,40	0,67	1,62	0	0	11752
Cbx5	25,99	0,74	43,45	1,17	1,62	0	0	12419
Extl3	6,60	0,20	11,03	0,08	1,62	0	0	54616
1700025G04Rik	2,89	0,08	4,82	0,07	1,62	1,11022E-16	3,99131E-15	69399
Sh3bp1	11,86	0,18	19,87	1,23	1,62	0	0	20401
Tmem132a	10,12	0,68	17,00	1,08	1,62	0	0	98170
Sema6c	1,68	0,14	2,82	0,31	1,62	8,51283E-07	1,11375E-05	20360
Uhmk1	6,23	0,69	10,35	0,50	1,62	5,7272E-12	1,36655E-10	16589
Pcsk9	2,00	0,34	3,34	0,89	1,61	0,000123546	0,00117695	100102
Ptk2b	3,35	0,21	5,62	0,30	1,61	9,84657E-13	2,54018E-11	19229
1810022K09Rik	17,66	0,74	29,28	1,46	1,61	3,10862E-15	1,01401E-13	69126
Gm15151	6,72	1,00	11,06	1,37	1,61	0,001937878	0,014637228	102637290
Fam124a	2,87	0,22	4,75	0,23	1,61	3,17003E-11	7,05477E-10	629059
Klhl42	2,79	0,17	4,61	0,40	1,61	5,05151E-14	1,48479E-12	232539
Megf9	1,60	0,17	2,63	0,17	1,61	1,3924E-05	0,000153661	230316
Tspan17	4,20	0,43	7,00	0,12	1,61	2,48505E-08	4,02294E-07	74257
Fut10	4,07	0,04	6,75	0,36	1,61	0	0	171167
Gcnt1	6,00	0,20	9,94	1,12	1,61	1,11022E-16	3,99131E-15	14537
Podnl1	1,42	0,25	2,39	0,30	1,61	0,000195843	0,001800113	244550
Gm8041	5,20	0,48	8,55	1,60	1,61	0,000341527	0,002998843	666315
Lsm6	8,31	0,15	13,82	0,23	1,61	0	0	78651
Smim15	13,94	0,62	23,03	1,31	1,61	1,11022E-16	3,99131E-15	75616
Epb41l5	1,13	0,07	1,87	0,14	1,61	2,83928E-12	7,01998E-11	226352
Srsf1	36,46	2,60	60,41	4,92	1,61	0	0	110809
Fam69a	4,60	0,59	7,60	1,09	1,61	5,71892E-08	8,79161E-07	67266
Ankrd52	17,80	0,15	29,53	1,93	1,61	0	0	237615
Tsacc	1,26	0,68	2,04	0,50	1,60	0,082769995	0,381198432	76927
Tesk2	2,84	0,35	4,78	0,57	1,60	4,26711E-07	5,82069E-06	230661
4933431E20Rik	1,25	0,08	2,06	0,12	1,60	9,81152E-10	1,85854E-08	329735
Borcs8	6,39	0,36	10,53	0,32	1,60	2,67564E-13	7,30612E-12	72368
Acot1	1,06	0,25	1,75	0,43	1,60	0,014790663	0,090084467	26897
Rsu1	29,51	0,64	48,78	1,54	1,60	0	0	20163
Atp5k	61,00	3,82	100,94	2,96	1,60	1,64313E-13	4,58803E-12	11958
Pld2	4,11	0,17	6,80	0,14	1,60	1,77636E-15	5,93394E-14	18806
Fdft1	19,29	1,31	31,90	0,42	1,60	0	0	14137
Trim7	1,73	0,16	2,83	0,22	1,60	4,48181E-06	5,3303E-05	94089
Rprm	1,17	0,24	1,94	0,18	1,60	0,006266058	0,042159092	67874
Stat2	10,02	0,77	16,57	1,60	1,60	0	0	20847
Gpt	2,28	0,36	3,79	0,43	1,60	5,32255E-05	0,000536889	76282
1700007K13Rik	1,33	0,33	2,18	0,51	1,60	0,035412488	0,190530507	69327
Ccdc124	18,19	0,88	30,29	2,57	1,60	7,21645E-15	2,28396E-13	234388
Tgfa	1,00	0,12	1,64	0,21	1,60	2,87892E-05	0,000302813	21802
Pitpnm2	3,11	0,10	5,14	0,29	1,60	4,44089E-16	1,54436E-14	19679
Man1c1	2,84	0,13	4,67	0,10	1,60	4,11648E-12	9,99952E-11	230815
Usp5	29,95	1,72	49,47	1,08	1,60	0	0	22225

Arrb1	2,25	0,23	3,74	0,13	1,60	1,94622E-13	5,40138E-12	109689
Cenpi	6,03	0,41	9,90	0,53	1,60	3,44169E-15	1,11235E-13	102920
1700020I14Rik	3,82	0,27	6,25	0,33	1,59	4,10783E-15	1,32484E-13	66602
Zc3h4	7,48	0,12	12,30	0,74	1,59	0	0	330474
Gm29779	34,04	4,55	55,99	5,47	1,59	1,68035E-10	3,48285E-09	101056102
Smarcd3	2,40	0,25	3,93	0,20	1,59	1,83717E-09	3,36852E-08	66993
Fndc3c1	1,14	0,09	1,85	0,18	1,59	7,71247E-06	8,85058E-05	333564
Fam134c	16,97	0,32	27,87	0,69	1,59	0	0	67998
Gm6560	10,73	1,66	17,70	2,44	1,59	5,29574E-09	9,1977E-08	625174
Nr2f2	7,94	0,22	13,03	0,48	1,59	0	0	11819
Fbxw4	2,58	0,13	4,24	0,10	1,59	7,423E-11	1,60091E-09	30838
Ldlrad4	2,59	0,19	4,25	0,46	1,59	6,21725E-15	1,98557E-13	52662
Map2k3	18,08	0,97	29,64	0,63	1,59	0	0	26397
Aqp1	10,89	0,82	17,93	0,30	1,59	6,66134E-16	2,2956E-14	11826
Pdss2	1,64	0,19	2,68	0,38	1,59	5,11227E-06	6,02842E-05	71365
Nudt5	6,41	0,86	10,45	0,74	1,59	7,88258E-15	2,48791E-13	53893
Depdc1a	5,06	0,30	8,29	0,72	1,59	1,1362E-12	2,91343E-11	76131
Ptgr1	7,32	0,36	12,02	0,06	1,59	2,2693E-12	5,65964E-11	67103
Gm11478	2,63	0,49	4,33	1,11	1,58	0,010266323	0,065528353	100504632
Cenpc1	7,08	0,40	11,56	0,38	1,58	6,66134E-16	2,2956E-14	12617
Fzd1	2,96	0,11	4,86	0,59	1,58	1,21691E-07	1,79477E-06	14362
Tuba4a	9,97	1,09	16,36	1,05	1,58	2,88658E-15	9,44277E-14	22145
Acot13	22,29	2,44	36,20	1,82	1,58	5,50426E-12	1,31886E-10	66834
Ndufb3	18,12	0,99	29,46	1,55	1,58	2,41311E-11	5,43753E-10	66495
Pomgnt2	2,21	0,31	3,66	0,49	1,58	1,28007E-05	0,000142086	215494
Colec12	28,56	0,52	46,57	1,05	1,58	0	0	140792
Sned1	2,73	0,21	4,46	0,32	1,58	3,21965E-15	1,04576E-13	208777
Adcy6	11,50	0,44	18,77	0,38	1,58	0	0	11512
Rnf26	11,55	1,41	18,94	0,60	1,58	7,10543E-15	2,25194E-13	213211
Rom1	2,11	0,55	3,49	0,12	1,58	0,000432207	0,003724336	19881
2610001J05Rik	13,85	0,67	22,47	0,88	1,58	4,44089E-16	1,54436E-14	66520
Tspan12	3,63	0,41	5,88	1,22	1,58	8,18302E-07	1,07336E-05	269831
Pros1	4,99	0,47	8,07	0,23	1,58	2,12252E-12	5,29936E-11	19128
Ctdspl	5,11	0,41	8,37	0,16	1,58	2,62013E-14	7,92532E-13	69274
Gsta4	7,38	0,42	11,88	1,20	1,58	4,55688E-06	5,41677E-05	14860
Bub1	7,52	0,66	12,26	0,64	1,58	7,77156E-16	2,66217E-14	12235
Swi5	96,36	2,01	156,58	6,41	1,58	0	0	72931
Gm2676	1,94	0,33	3,10	0,34	1,58	0,000152427	0,001428004	100040234
Plppr3	1,46	0,23	2,40	0,42	1,57	1,13269E-05	0,000126618	216152
Sap30l	7,96	0,47	12,78	1,25	1,57	2,34184E-07	3,32014E-06	50724
Apaf1	13,45	1,00	21,81	0,67	1,57	0	0	11783
Trp53inp1	23,89	2,65	38,77	3,31	1,57	0	0	60599
Bahcc1	2,75	0,06	4,46	0,60	1,57	1,11022E-15	3,76366E-14	268515
Nedd9	7,53	0,65	12,25	0,71	1,57	3,33067E-16	1,16357E-14	18003
Tshz1	6,40	0,44	10,43	0,13	1,57	2,22045E-16	7,82284E-15	110796
Calm3	40,55	1,60	65,95	1,83	1,57	0	0	12315

Rida	12,55	1,20	20,19	0,88	1,57	1,36494E-10	2,86413E-09	15473
Lsm2	43,94	1,47	71,21	1,91	1,57	0	0	27756
Gm4735	1,84	0,82	3,00	1,15	1,57	0,020573877	0,119944394	103324
B230118H07Rik	2,20	0,29	3,52	0,15	1,57	1,72972E-05	0,000188748	68170
Pdp2	5,11	0,07	8,28	0,22	1,57	3,88578E-15	1,25499E-13	382051
Ncapd2	29,16	2,33	47,38	2,76	1,57	0	0	68298
Srrt	18,05	0,66	29,27	2,26	1,57	0	0	83701
Dlgap5	14,05	0,64	22,83	0,87	1,57	1,11022E-16	3,99131E-15	218977
Ttc7	3,24	0,32	5,28	0,40	1,57	9,17044E-14	2,63456E-12	225049
Elmo2	9,88	0,28	15,99	0,50	1,57	0	0	140579
Sap18	9,14	0,74	14,78	0,91	1,57	2,44249E-15	8,04177E-14	20220
Tubb6	81,71	7,60	132,27	7,54	1,57	0	0	67951
Zfp41	2,37	0,09	3,81	0,14	1,57	9,45052E-10	1,79686E-08	22701
Evc	5,20	0,29	8,41	0,19	1,57	4,10783E-15	1,32484E-13	59056
Sdc3	10,74	0,68	17,40	0,85	1,57	0	0	20970
C87436	3,61	0,44	5,78	0,08	1,57	1,76886E-11	4,04558E-10	232196
Mcam	6,08	0,37	9,86	0,28	1,57	1,03717E-12	2,67078E-11	84004
Vgll3	12,22	0,78	19,75	0,74	1,57	0	0	73569
Erc1	7,42	0,16	12,01	0,65	1,57	0	0	111173
Mfsd12	6,61	0,17	10,70	0,19	1,57	2,33147E-14	7,08497E-13	73822
Kif23	15,54	1,17	25,17	0,83	1,56	0	0	71819
Trp53inp2	6,41	0,61	10,37	0,36	1,56	1,44329E-15	4,84253E-14	68728
Smim11	16,30	1,37	26,56	1,54	1,56	2,54604E-08	4,10859E-07	68936
2900097C17Rik	36,81	1,32	59,33	2,03	1,56	0	0	347740
Eme1	4,44	0,19	7,18	0,20	1,56	2,08063E-09	3,79666E-08	268465
Col1a2	162,54	12,23	262,15	29,30	1,56	1,3231E-10	2,78218E-09	12843
Kat2b	6,75	0,81	10,83	0,80	1,56	3,10862E-15	1,01401E-13	18519
1110059G10Rik	2,19	0,13	3,51	0,26	1,56	8,31378E-08	1,24952E-06	66202
Lrrc57	4,54	0,78	7,27	0,99	1,56	1,39491E-06	1,77328E-05	66606
Pxdn	12,80	0,16	20,65	1,21	1,56	0	0	69675
Bub1b	13,43	1,58	21,69	0,82	1,56	0	0	12236
Pbx2	7,74	0,15	12,45	0,86	1,56	3,25961E-13	8,83222E-12	18515
Nes	21,98	0,59	35,45	1,64	1,56	0	0	18008
Pagr1a	7,48	0,71	12,10	1,06	1,56	1,80225E-09	3,30979E-08	67278
Apoe	1,27	0,50	2,05	0,19	1,56	0,010052402	0,064288455	11816
Gm13710	1,14	0,08	1,82	0,16	1,56	8,10664E-06	9,26806E-05	672763
Fstl1	63,09	1,11	101,62	5,36	1,56	0	0	14314
Napepld	2,11	0,18	3,38	0,39	1,56	3,94098E-09	6,94758E-08	242864
Klra4	3,94	0,06	6,36	0,68	1,56	5,17063E-05	0,000522371	16635
Gprasp1	2,14	0,26	3,44	0,11	1,56	3,53521E-07	4,87756E-06	67298
Dido1	2,92	0,27	4,69	0,34	1,56	0	0	23856
Borcs6	7,51	0,36	12,17	0,73	1,56	1,1523E-10	2,43918E-09	71923
Ap1s1	19,61	0,36	31,55	1,74	1,56	2,22045E-16	7,82284E-15	11769
Aldh4a1	3,83	0,20	6,13	0,47	1,56	6,68671E-11	1,44689E-09	212647
H3f3a	93,11	2,50	149,74	1,32	1,56	0	0	15078
Ndrg2	1,23	0,15	1,96	0,23	1,56	0,000196504	0,001805101	29811

Dstn	117,47	1,33	188,67	6,38	1,56	0	0	56431
Camkk1	1,51	0,20	2,43	0,15	1,56	7,46527E-06	8,59276E-05	55984
Hlcs	1,04	0,02	1,66	0,06	1,56	4,98179E-08	7,69981E-07	110948
Dag1	25,74	0,79	41,33	2,46	1,55	0	0	13138
Ahsa2	5,50	0,72	8,79	1,70	1,55	2,29882E-08	3,72805E-07	268390
Bbs12	1,09	0,16	1,73	0,11	1,55	6,76285E-09	1,16485E-07	241950
Nt5c2	6,50	0,57	10,44	0,45	1,55	3,33067E-16	1,16357E-14	76952
Gm8451	3,15	0,58	5,15	0,98	1,55	0,011747499	0,073687543	667090
Incenp	22,43	2,17	35,98	1,18	1,55	0	0	16319
Ttc28	1,61	0,08	2,58	0,55	1,55	7,70095E-08	1,16276E-06	209683
Spaca6	1,13	0,05	1,82	0,29	1,55	2,95305E-11	6,5925E-10	75202
Eml1	5,76	0,56	9,23	0,50	1,55	1,77636E-14	5,4597E-13	68519
Pcna	125,45	1,85	200,39	8,45	1,55	0	0	18538
Pard6b	2,91	0,22	4,64	0,15	1,55	7,53955E-09	1,29183E-07	58220
Gm7666	54,30	4,12	86,90	10,21	1,55	1,11022E-16	3,99131E-15	665511
Kifc1	31,54	4,01	50,49	1,55	1,55	0	0	100502766
Mthfd2l	2,97	0,14	4,68	0,52	1,55	1,28994E-05	0,000143147	665563
Nr2f6	6,65	0,47	10,61	0,49	1,55	3,58602E-14	1,07194E-12	13864
St3gal3	4,65	0,45	7,43	0,78	1,55	4,08728E-07	5,59039E-06	20441
Prkar2a	12,79	0,48	20,39	0,10	1,55	1,11022E-16	3,99131E-15	19087
Kif5a	1,07	0,05	1,73	0,17	1,55	1,47014E-07	2,14818E-06	16572
Ammecr1	3,89	0,04	6,19	0,62	1,55	9,37754E-10	1,78446E-08	56068
Smo	24,87	0,50	39,67	0,57	1,54	0	0	319757
Palld	9,42	0,19	15,00	0,27	1,54	0	0	72333
Atf7ip	3,35	0,05	5,35	0,13	1,54	4,44089E-16	1,54436E-14	54343
Dnlz	9,06	0,34	14,44	0,37	1,54	1,54432E-13	4,33594E-12	52838
Nxpe3	1,22	0,10	1,94	0,15	1,54	1,22452E-08	2,04675E-07	385658
Timm8b	34,40	2,03	54,62	0,04	1,54	1,15278E-11	2,6821E-10	30057
Tfdp1	51,73	0,49	82,29	3,37	1,54	0	0	21781
Ddx46	14,33	0,39	22,77	1,04	1,54	0	0	212880
Rassf7	7,14	0,93	11,47	1,13	1,54	5,65407E-07	7,58378E-06	66985
Ptgfrn	7,06	0,58	11,26	0,64	1,54	1,44329E-15	4,84253E-14	19221
Rfx7	7,25	0,65	11,51	0,22	1,54	1,11022E-16	3,99131E-15	319758
LOC108168103	10,04	0,62	16,08	1,09	1,54	1,56016E-08	2,57765E-07	108168103
Sertad4	6,37	0,34	10,09	0,66	1,54	6,45706E-13	1,70125E-11	214791
B4galt5	8,54	0,68	13,63	0,22	1,54	7,88258E-15	2,48791E-13	56336
Gm14567	10,57	3,11	16,58	1,26	1,54	0,000852532	0,006943605	100046796
Tyro3	5,32	0,15	8,46	0,32	1,54	1,59317E-13	4,45941E-12	22174
Vamp5	3,30	0,18	5,21	0,48	1,54	5,429E-05	0,000547265	53620
Anxa1	327,56	14,15	520,37	11,82	1,54	0	0	16952
Cxx1a	15,00	1,76	23,94	2,25	1,54	8,43259E-12	1,9811E-10	66158
Gulp1	2,61	0,25	4,09	0,30	1,54	6,41001E-08	9,77521E-07	70676
Fzr1	19,85	1,80	31,63	1,99	1,54	1,44329E-15	4,84253E-14	56371
Klhl25	2,45	0,29	3,94	0,25	1,54	2,18726E-08	3,55689E-07	207952
Sgol2a	7,05	0,28	11,17	0,19	1,54	6,66134E-15	2,11706E-13	68549
Gm7049	1,60	0,42	2,56	0,01	1,54	0,000929147	0,007514123	630951

Zfp217	1,72	0,14	2,74	0,04	1,53	4,17466E-10	8,26356E-09	228913
Efnb2	5,29	0,15	8,38	0,16	1,53	2,22045E-16	7,82284E-15	13642
Elk3	20,56	1,37	32,59	1,17	1,53	0	0	13713
Obfc1	14,10	0,49	22,23	1,45	1,53	2,89768E-14	8,73024E-13	108689
Irak4	2,51	0,21	3,95	0,32	1,53	1,19955E-07	1,77031E-06	266632
Gnptab	9,43	0,46	14,90	0,36	1,53	6,66134E-16	2,2956E-14	432486
Ect2	15,17	0,15	24,03	0,33	1,53	1,11022E-16	3,99131E-15	13605
Rbx1	41,72	2,65	65,85	4,08	1,53	1,11022E-16	3,99131E-15	56438
Foxk2	16,17	0,05	25,58	0,33	1,53	0	0	68837
B4galnt1	7,87	0,58	12,52	0,88	1,53	9,64784E-14	2,7665E-12	14421
Lacc1	4,03	0,34	6,34	0,15	1,53	1,83705E-09	3,36852E-08	210808
Cyb5a	79,01	0,73	124,71	6,53	1,53	1,11022E-16	3,99131E-15	109672
Ndrg4	9,32	0,19	14,75	0,47	1,53	1,5099E-14	4,66897E-13	234593
Safb	22,39	1,08	35,36	0,38	1,53	0	0	224903
Dzip1l	3,62	0,18	5,73	0,25	1,53	1,31872E-12	3,36032E-11	72507
Ak1	16,89	0,44	26,73	0,30	1,53	1,11022E-16	3,99131E-15	11636
Slc10a7	3,04	0,18	4,77	0,46	1,53	6,4211E-11	1,39073E-09	76775
Fan1	1,16	0,15	1,83	0,35	1,53	0,000159585	0,001489277	330554
Slc8b1	6,32	0,27	10,02	0,65	1,53	2,54254E-11	5,71791E-10	170756
Ptrhd1	2,37	0,10	3,73	0,24	1,53	3,00691E-08	4,79984E-07	69709
Ppp1r9b	16,84	0,79	26,63	1,05	1,53	1,11022E-16	3,99131E-15	217124
Ppp2r3d	1,66	0,13	2,64	0,19	1,53	0,000244241	0,002202934	19054
Mrpl51	27,40	0,82	43,00	2,35	1,53	6,55032E-15	2,08612E-13	66493
Kif4	8,09	0,56	12,80	0,40	1,53	7,77156E-16	2,66217E-14	16571
Tmeff2	11,95	0,56	18,78	1,55	1,53	1,18794E-14	3,70593E-13	56363
Crat	8,92	0,19	14,07	0,67	1,53	6,99441E-15	2,21829E-13	12908
Mpp2	1,34	0,16	2,12	0,11	1,52	4,44559E-05	0,00045272	50997
Lrrc20	7,94	0,31	12,52	0,05	1,52	1,00286E-12	2,5857E-11	216011
Tk1	12,84	1,48	20,30	0,79	1,52	7,42739E-13	1,94682E-11	21877
2700081O15Rik	7,45	0,44	11,71	0,24	1,52	1,82077E-14	5,5812E-13	108899
Arpc5	43,88	0,81	68,94	2,55	1,52	0	0	67771
5031439G07Rik	13,93	0,65	21,94	0,77	1,52	1,11022E-16	3,99131E-15	223739
Kctd17	19,37	0,58	30,57	1,07	1,52	7,77156E-16	2,66217E-14	72844
C1qtnf6	9,43	0,17	14,85	0,51	1,52	8,60423E-13	2,23989E-11	72709
Ldha	72,51	7,03	114,16	1,44	1,52	0	0	16828
Wbp1l	16,35	0,79	25,81	0,99	1,52	2,22045E-16	7,82284E-15	226178
Cox7a2	48,51	0,60	76,28	1,61	1,52	3,17524E-14	9,54762E-13	12866
C2cd2	1,36	0,07	2,15	0,07	1,52	1,01361E-08	1,70669E-07	207781
Zfp651	2,64	0,19	4,19	0,29	1,52	8,05124E-11	1,73232E-09	270210
Nudcd3	10,34	0,27	16,23	0,60	1,52	1,15463E-14	3,60694E-13	209586
Sertad1	12,78	1,36	20,30	0,96	1,52	1,18621E-10	2,504E-09	55942
Slc9a5	3,14	0,37	4,99	0,35	1,52	5,68644E-11	1,23629E-09	277973
Arpc4	28,53	0,70	44,77	1,52	1,52	3,33067E-16	1,16357E-14	68089
Tmem64	5,91	0,32	9,24	0,52	1,52	5,67768E-13	1,50283E-11	100201
Cerk	3,82	0,16	5,99	0,24	1,52	5,15609E-11	1,1258E-09	223753
Gipc1	34,55	4,01	54,39	1,93	1,52	9,99201E-16	3,40241E-14	67903

Pcyox1	23,11	0,19	36,22	1,98	1,52	0	0	66881
LOC102640526	1,16	0,20	1,83	0,21	1,52	0,000165333	0,001539148	102640526
Arsj	2,10	0,38	3,33	0,28	1,52	1,63144E-05	0,000178704	271970
Pip4k2a	1,18	0,01	1,86	0,13	1,52	2,25936E-07	3,21416E-06	18718
Cetn2	17,89	1,11	28,08	0,71	1,52	1,14722E-11	2,67187E-10	26370
Ophn1	1,03	0,04	1,60	0,11	1,52	3,2837E-08	5,20716E-07	94190
Emc8	9,12	0,31	14,25	0,84	1,52	7,99361E-15	2,51947E-13	18117
Mov10	5,81	0,21	9,14	0,26	1,52	8,22897E-12	1,93823E-10	17454
Hic1	2,73	0,35	4,31	0,20	1,52	1,98282E-06	2,46385E-05	15248
St6galnac6	7,42	0,22	11,64	0,54	1,52	2,267E-11	5,12089E-10	50935
Snrpd1	69,36	4,84	108,44	7,30	1,52	1,11022E-15	3,76366E-14	20641
Dusp19	2,89	0,23	4,47	0,41	1,52	0,000306719	0,002721405	68082
Skiv2l2	14,26	1,43	22,36	0,30	1,52	5,88418E-15	1,88183E-13	72198
Ska3	7,98	0,71	12,48	0,83	1,52	4,17257E-11	9,17625E-10	219114
Galnt10	7,43	0,38	11,64	0,64	1,52	3,74145E-14	1,11622E-12	171212
Ralgps2	1,44	0,06	2,24	0,18	1,52	3,15243E-06	3,82986E-05	78255
Tpgs2	3,14	0,26	4,89	0,27	1,52	5,89162E-12	1,40505E-10	66648
Rassf3	4,70	0,19	7,38	0,21	1,52	9,03162E-11	1,93508E-09	192678
Rtn3	38,31	0,69	59,85	3,71	1,51	0	0	20168
Pten	12,21	0,60	19,06	1,13	1,51	0	0	19211
S100a10	226,76	9,88	354,46	12,22	1,51	0	0	20194
Zfc3h1	6,36	0,61	9,92	0,45	1,51	1,14353E-14	3,5747E-13	216345
Zbtb7a	6,96	0,22	10,88	0,63	1,51	9,54792E-15	2,99904E-13	16969
Kif18a	4,58	0,12	7,14	0,16	1,51	4,93318E-10	9,66056E-09	228421
Pip5k1c	25,79	1,57	40,36	1,10	1,51	0	0	18717
Mllt6	5,41	0,19	8,45	0,22	1,51	4,22995E-14	1,25459E-12	246198
Sfpq	25,34	0,55	39,52	0,59	1,51	0	0	71514
Ctps2	6,75	0,18	10,54	0,14	1,51	4,4631E-14	1,32032E-12	55936
Dpp3	21,34	2,27	33,36	0,71	1,51	1,77636E-15	5,93394E-14	75221
Pgam1	119,57	3,35	186,42	3,03	1,51	0	0	18648
Ppp1r12b	3,40	0,42	5,32	0,18	1,51	4,11893E-14	1,22325E-12	329251
Mfsd14a	9,73	0,72	15,09	0,72	1,51	6,32827E-15	2,01962E-13	15247
Asf1b	16,27	0,62	25,43	1,67	1,51	3,01037E-12	7,42693E-11	66929
Tapbp	37,56	3,37	58,64	1,67	1,51	1,11022E-16	3,99131E-15	21356
Tmem173	3,96	0,15	6,14	0,17	1,51	1,21859E-07	1,79667E-06	72512
Pear1	12,13	1,02	18,95	1,50	1,51	2,77556E-15	9,0991E-14	73182
Tead2	21,62	1,09	33,67	1,63	1,51	1,46549E-14	4,53778E-13	21677
Gm20056	2,95	0,74	4,71	0,71	1,51	0,00271743	0,019949179	100504089
Tcaf2	3,03	0,21	4,71	0,32	1,51	2,56502E-06	3,15135E-05	232748
Mfsd7b	4,18	0,17	6,45	0,29	1,51	9,49301E-11	2,02918E-09	226844
Eps8l2	11,90	0,29	18,60	1,14	1,51	1,64091E-13	4,58463E-12	98845
Sh3yl1	3,97	0,29	6,19	0,67	1,51	4,50166E-05	0,000458022	24057
Ankle1	1,40	0,27	2,19	0,39	1,51	0,001762097	0,013462831	234396
4931428F04Rik	1,36	0,07	2,11	0,23	1,51	2,26596E-07	3,22255E-06	74356
Xxylt1	4,64	0,16	7,14	0,60	1,51	6,35147E-10	1,22959E-08	268880
Sardh	3,63	0,35	5,68	0,24	1,50	2,87765E-10	5,80158E-09	192166

Gm5518	1,20	0,39	1,94	0,51	1,50	0,042655541	0,223217789	433238
Vsig10	4,96	0,15	7,69	0,20	1,50	5,80666E-11	1,26183E-09	231668
Apex2	2,19	0,14	3,40	0,15	1,50	2,69476E-08	4,32418E-07	77622
Btg2	10,99	0,25	16,99	1,13	1,50	3,90799E-13	1,05143E-11	12227
Cxx1c	2,15	0,17	3,28	0,81	1,50	0,013930314	0,085527347	72865
Sdhaf4	8,04	0,70	12,33	0,95	1,50	0,000153227	0,00143521	68002
Pnpla6	6,84	0,24	10,63	0,31	1,50	4,1489E-13	1,11168E-11	50767
Eno3	4,94	0,88	7,68	0,50	1,50	1,97247E-06	2,45166E-05	13808
Arhgap19	2,75	0,11	4,23	0,18	1,50	1,95749E-09	3,58051E-08	71085
Slc25a1	33,77	2,51	52,61	2,29	1,50	6,66134E-15	2,11706E-13	13358
Nudt19	3,04	0,09	4,63	0,65	1,50	0,000106883	0,001027629	110959
Ckap2l	16,92	2,06	26,28	0,53	1,50	9,21485E-15	2,9004E-13	70466
Slc46a1	1,95	0,22	3,03	0,21	1,50	0,000678939	0,005637726	52466
Casp3	31,63	0,73	48,86	4,17	1,50	6,66134E-16	2,2956E-14	12367
Gpatch2l	5,72	0,30	3,93	0,17	-1,50	1,37346E-12	3,49396E-11	70373
Map3k2	9,48	0,85	6,51	0,22	-1,50	2,10942E-15	6,98537E-14	26405
AU040320	9,18	0,67	6,32	0,36	-1,50	5,42344E-13	1,44304E-11	100317
Gm5884	39,88	3,67	27,49	0,76	-1,50	4,77396E-15	1,53212E-13	545878
Foxc2	7,76	0,31	5,32	0,17	-1,50	2,51154E-09	4,53952E-08	14234
Rad18	24,29	0,93	16,69	0,43	-1,50	4,78506E-14	1,4101E-12	58186
Mgme1	7,88	0,37	5,39	0,31	-1,50	4,67061E-10	9,18962E-09	74528
Shmt1	34,84	2,06	23,89	1,71	-1,50	1,27676E-14	3,97218E-13	20425
Abhd10	7,89	0,49	5,41	0,05	-1,50	3,81111E-10	7,57012E-09	213012
Mtmr3	25,02	0,80	17,21	0,12	-1,50	1,11022E-16	3,99131E-15	74302
Mbip	9,19	0,70	6,27	0,62	-1,50	5,54781E-06	6,51683E-05	217588
Lnx2	4,84	0,31	3,35	0,20	-1,50	1,2927E-09	2,41077E-08	140887
Zfp935	8,00	0,68	5,47	0,26	-1,50	4,57936E-09	8,02659E-08	71508
Ptrh1	55,51	2,19	38,19	0,84	-1,50	4,01457E-13	1,07694E-11	329384
Dnajc27	2,18	0,05	1,50	0,14	-1,50	5,81249E-07	7,77804E-06	217378
2410131K14Rik	9,74	0,46	6,71	0,27	-1,50	2,6403E-10	5,35372E-09	76792
Foxn3	4,95	0,17	3,39	0,22	-1,50	1,78335E-12	4,4967E-11	71375
Nifk	38,92	0,78	26,74	0,42	-1,50	1,56541E-14	4,82757E-13	67949
Zxdb	4,36	0,42	3,01	0,31	-1,50	4,69845E-10	9,23248E-09	668166
Zfp760	11,06	0,42	7,55	0,99	-1,50	7,9925E-13	2,08657E-11	240034
Prmt7	23,23	1,24	15,98	0,43	-1,50	4,28546E-14	1,27023E-12	214572
Gm19038	1,01	0,47	0,69	0,30	-1,50	0,137922661	0,563574159	100418152
Usf1	28,54	2,45	19,68	1,41	-1,50	1,0314E-13	2,9446E-12	22278
Sipa1l2	7,08	0,06	4,87	0,18	-1,51	5,80647E-13	1,53425E-11	244668
Nim1k	1,60	0,30	1,07	0,12	-1,51	0,000978411	0,007877726	245269
Ccnh	22,55	0,10	15,39	2,01	-1,51	6,95E-13	1,82692E-11	66671
Nat10	11,15	0,67	7,66	0,34	-1,51	5,59552E-14	1,63733E-12	98956
Mnat1	16,40	0,69	11,15	1,00	-1,51	1,81E-12	4,55135E-11	17420
Ddx27	48,13	1,06	33,05	0,63	-1,51	2,22045E-16	7,82284E-15	228889
Ddx51	8,06	0,44	5,54	0,14	-1,51	3,74922E-12	9,16088E-11	69663
Arhgap29	26,04	0,98	17,85	0,37	-1,51	1,11022E-16	3,99131E-15	214137
Pex3	15,06	0,20	10,30	1,05	-1,51	7,27685E-12	1,72106E-10	56535

Gpr146	4,07	0,31	2,80	0,03	-1,51	2,42346E-09	4,38725E-08	80290
Mcc	3,91	0,20	2,69	0,11	-1,51	1,13698E-12	2,91343E-11	328949
Renbp	69,96	4,43	47,83	4,81	-1,51	1,33227E-15	4,48648E-14	19703
Ldlrad3	12,39	0,60	8,48	0,32	-1,51	3,56382E-13	9,62798E-12	241576
Tpd52l1	2,92	0,25	2,03	0,50	-1,51	0,003917973	0,027672701	21987
Fbxl4	6,11	0,81	4,18	0,57	-1,51	3,33707E-06	4,0381E-05	269514
Slc25a16	12,59	0,78	8,61	0,67	-1,51	2,81897E-12	6,97729E-11	73132
Oxld1	2,73	0,43	1,90	0,65	-1,51	0,04088955	0,215638427	66431
Maml1	9,39	0,37	6,44	0,08	-1,51	4,74065E-14	1,39881E-12	103806
Ktn1	26,57	0,76	18,14	0,73	-1,51	0	0	16709
Thnsl1	3,33	0,51	2,25	0,15	-1,51	8,6933E-06	9,89182E-05	208967
Pus1	20,55	1,63	14,17	1,29	-1,51	2,33391E-12	5,81445E-11	56361
Hif1a	33,93	1,76	23,17	0,84	-1,51	0	0	15251
Pspc1	27,65	1,30	18,85	0,42	-1,51	2,34257E-14	7,11398E-13	66645
Plin2_2	61,64	5,35	42,13	1,96	-1,51	1,11022E-16	3,99131E-15	11520
Zfp608	2,42	0,07	1,66	0,10	-1,51	2,60324E-11	5,84006E-10	269023
Unk	8,52	0,42	5,85	0,28	-1,51	2,31815E-13	6,36412E-12	217331
Irgm2	4,78	0,14	3,25	0,11	-1,51	1,46724E-08	2,43026E-07	54396
Tmem39a	9,49	0,43	6,48	0,33	-1,51	5,53492E-11	1,20555E-09	67846
Zfp790	2,35	0,14	1,61	0,04	-1,51	8,74432E-09	1,48546E-07	233056
Ivns1abp	34,29	2,05	23,38	1,49	-1,51	0	0	117198
Trip6	47,04	3,37	32,23	2,26	-1,51	8,88178E-16	3,03566E-14	22051
Zfp58	5,28	0,74	3,61	0,18	-1,51	1,55776E-05	0,000170921	238693
Thap3	9,98	0,58	6,84	0,26	-1,51	7,03195E-07	9,29706E-06	69876
Tyw5	10,23	0,68	6,94	0,28	-1,51	3,48237E-09	6,18524E-08	68736
Fnip1	21,28	1,86	14,50	0,76	-1,51	1,11022E-16	3,99131E-15	216742
Tex10	24,41	0,14	16,64	0,91	-1,51	1,88738E-15	6,29562E-14	269536
Snapc4	7,16	0,12	4,91	0,36	-1,51	1,78757E-12	4,50237E-11	227644
Kansl2-ps	40,80	1,23	27,70	3,59	-1,51	1,78746E-14	5,49014E-13	233391
Lbh	13,91	1,32	9,50	0,33	-1,51	8,47211E-13	2,20801E-11	77889
Eif3a	197,33	3,46	134,57	1,72	-1,51	0	0	13669
Fndc3a	13,87	0,91	9,43	0,18	-1,51	7,77156E-16	2,66217E-14	319448
Slc25a30	9,75	0,93	6,67	0,36	-1,51	1,09401E-12	2,8112E-11	67554
Fam151b	1,30	0,30	0,89	0,10	-1,51	0,046666609	0,240208901	73942
Far1os	1,07	0,21	0,73	0,10	-1,51	3,78299E-07	5,1866E-06	69295
Zfyve9	9,78	0,28	6,65	0,15	-1,51	3,26406E-14	9,80181E-13	230597
Dfna5	4,38	0,65	3,00	0,03	-1,51	7,37526E-06	8,49984E-05	54722
Apmap	16,97	0,21	11,55	0,43	-1,52	1,18794E-14	3,70593E-13	71881
Wdr74	41,65	3,04	28,49	1,12	-1,52	1,19682E-13	3,3978E-12	107071
Ppp1r15b	24,59	0,23	16,73	0,29	-1,52	1,11022E-16	3,99131E-15	108954
Gyg	28,56	1,15	19,37	1,13	-1,52	6,65024E-14	1,93729E-12	27357
Ice2	4,35	0,20	2,93	0,49	-1,52	1,19796E-06	1,53485E-05	93697
Map1b	16,73	0,51	11,39	1,35	-1,52	0	0	17755
Rps15a-ps6	10,24	0,96	7,03	1,01	-1,52	8,53512E-05	0,00083535	100042173
Cxcl16	9,04	1,53	6,16	0,34	-1,52	8,81063E-07	1,15041E-05	66102
Vapa	96,16	3,59	65,29	3,82	-1,52	0	0	30960

Ap1g2	4,40	0,29	2,95	0,30	-1,52	2,17133E-08	3,53257E-07	11766
Porcn	4,32	0,18	2,96	0,47	-1,52	9,03629E-05	0,000880822	53627
Mrpl2	49,01	2,68	33,34	0,62	-1,52	5,4734E-14	1,60262E-12	27398
Plpp6	5,74	0,29	3,86	0,42	-1,52	1,17748E-08	1,97029E-07	74411
Zeb2	10,10	0,19	6,87	0,13	-1,52	3,33067E-16	1,16357E-14	24136
Rfx1	4,88	0,54	3,35	0,27	-1,52	9,12067E-07	1,18886E-05	19724
Gm41291	1,05	0,24	0,68	0,30	-1,52	0,104758752	0,458169368	105245911
Senp2	17,59	0,73	11,97	0,41	-1,52	1,66533E-15	5,5712E-14	75826
Sp3	45,84	1,44	31,05	1,45	-1,52	0	0	20687
Cul5	16,40	1,30	11,12	0,43	-1,52	3,33067E-16	1,16357E-14	75717
Lpar4	2,76	0,15	1,84	0,26	-1,52	1,6661E-05	0,00018224	78134
Alg5	31,77	1,47	21,53	0,92	-1,52	2,07834E-13	5,73674E-12	66248
Ptbp2	8,60	0,47	5,80	0,27	-1,52	8,27027E-12	1,94696E-10	56195
Dis3	17,53	0,34	11,88	0,45	-1,52	1,11022E-16	3,99131E-15	72662
Scyl2	23,48	0,39	15,92	0,41	-1,52	1,11022E-16	3,99131E-15	213326
Eapp	15,43	0,36	10,36	1,07	-1,52	3,99812E-11	8,8053E-10	66266
Aftph	20,38	0,64	13,76	0,70	-1,52	9,99201E-16	3,40241E-14	216549
Gps2	21,47	0,58	14,58	0,06	-1,52	4,67626E-13	1,24932E-11	56310
Rabggta	6,78	0,56	4,63	0,21	-1,52	2,92349E-09	5,23654E-08	56187
Gm7107	1,17	0,32	0,81	0,40	-1,52	0,08886923	0,403482173	633238
Zc3h7a	18,68	0,34	12,64	0,41	-1,52	1,9984E-15	6,64175E-14	106205
Heatr1	13,07	0,24	8,85	0,26	-1,52	5,55112E-16	1,92606E-14	217995
Ncoa3	12,66	0,16	8,62	0,71	-1,52	3,33067E-16	1,16357E-14	17979
B3gntl1	1,82	0,11	1,22	0,21	-1,52	1,49575E-05	0,000164393	210004
Aasdhppt	10,11	0,10	6,78	0,88	-1,52	1,9206E-11	4,38166E-10	67618
Bcs1l	6,24	0,57	4,23	0,11	-1,52	2,30218E-07	3,27102E-06	66821
Rpl27	1,73	0,35	1,16	1,35	-1,53	0,266912875	0,875332693	19942
Mbnl2	26,52	0,61	17,90	0,20	-1,53	0	0	105559
Mrm1	5,88	0,48	3,95	0,26	-1,53	2,88279E-08	4,61296E-07	217038
Sun2	83,30	7,89	56,35	5,04	-1,53	0	0	223697
Stx11	6,29	0,36	4,23	0,25	-1,53	2,91698E-09	5,22693E-08	74732
Naa30	7,86	0,69	5,28	0,17	-1,53	1,55698E-12	3,9411E-11	70646
Chchd4	14,62	1,87	9,83	1,20	-1,53	1,49881E-06	1,89588E-05	72170
Fdxr	4,98	0,16	3,43	0,75	-1,53	2,2837E-05	0,000244762	14149
Ggcx	10,16	0,46	6,85	0,56	-1,53	5,06706E-13	1,35136E-11	56316
LOC108169038	1,51	0,44	1,01	0,16	-1,53	0,042271897	0,22175302	108169038
Gm1943	7,35	1,02	4,85	0,58	-1,53	7,26287E-05	0,00071867	384864
Prr3	9,94	0,11	6,69	0,24	-1,53	1,38973E-11	3,21543E-10	75210
Fam204a	13,64	0,83	9,16	0,40	-1,53	3,37508E-14	1,01153E-12	76539
Sh2b3	13,14	0,73	8,89	0,32	-1,53	7,77156E-16	2,66217E-14	16923
Ankzf1	5,16	0,35	3,46	0,24	-1,53	1,72242E-06	2,15845E-05	52231
Ss18l1	1,52	0,14	1,02	0,08	-1,53	0,000110067	0,001054847	269397
Stk19	21,73	1,82	14,74	0,48	-1,53	1,38296E-10	2,90061E-09	54402
Ribc1	1,07	0,15	0,73	0,07	-1,53	0,022006507	0,127323363	66611
Fip1l1	40,69	0,11	27,42	0,53	-1,53	0	0	66899
Akirin1	27,26	1,50	18,33	1,01	-1,53	3,44169E-15	1,11235E-13	68050

Ighmbp2	5,23	0,14	3,55	0,25	-1,53	1,20434E-11	2,79779E-10	20589
Tango6	7,37	0,43	4,96	0,24	-1,53	3,54416E-12	8,67837E-11	272538
Ddx10	11,51	0,64	7,71	0,72	-1,53	5,55112E-16	1,92606E-14	77591
Cpeb1	6,32	0,24	4,26	0,22	-1,53	1,53766E-13	4,31989E-12	12877
Plekhf2	7,95	0,55	5,31	0,14	-1,53	1,43003E-10	2,98974E-09	71801
Tasp1	3,25	0,41	2,16	0,32	-1,53	2,01552E-06	2,50109E-05	75812
LOC102640673	5,82	0,47	3,89	0,56	-1,53	1,19137E-08	1,99279E-07	102640673
Ucp2	7,15	0,56	4,88	0,46	-1,53	8,77049E-06	9,97222E-05	22228
Fam134b	16,87	1,24	11,33	0,07	-1,53	9,99201E-16	3,40241E-14	66270
Smim10l1	15,32	0,14	10,26	0,71	-1,53	0	0	381820
4632415L05Rik	6,19	0,17	4,14	0,38	-1,54	4,6214E-11	1,01236E-09	70808
Poli	6,35	0,19	4,27	0,08	-1,54	9,70735E-12	2,27474E-10	26447
Nfkbib	6,28	0,45	4,22	0,63	-1,54	5,3005E-06	6,23949E-05	18036
Sh3bp5	9,58	1,01	6,49	0,61	-1,54	1,19447E-11	2,77627E-10	24056
Runx2	3,24	0,11	2,19	0,13	-1,54	1,98137E-11	4,51581E-10	12393
Rbm48	5,90	0,29	3,94	0,26	-1,54	1,30801E-08	2,17994E-07	269623
Smad9	1,83	0,06	1,22	0,06	-1,54	2,84653E-07	3,98215E-06	55994
LOC108167824	1,10	0,10	0,74	0,10	-1,54	4,73947E-05	0,000480935	108167824
Pwp1	40,54	0,44	27,21	0,44	-1,54	0	0	103136
Mir17hg	2,01	0,22	1,36	0,18	-1,54	0,00081779	0,006686822	75957
Coq3	4,71	0,23	3,13	0,35	-1,54	4,49461E-08	7,00599E-07	230027
B230217C12Rik	5,65	0,75	3,71	0,54	-1,54	0,000158326	0,001478132	68127
Phykpl	4,44	0,36	2,94	0,32	-1,54	8,72106E-06	9,91847E-05	72947
Sorbs1	2,03	0,08	1,38	0,13	-1,54	1,71274E-12	4,32342E-11	20411
Trmt1	34,01	0,74	22,85	0,87	-1,54	0	0	212528
Cep44	8,89	0,14	5,96	0,31	-1,54	2,25832E-10	4,61805E-09	382010
Ggct	3,10	0,87	2,10	0,08	-1,54	0,003301701	0,023732107	110175
1700020L24Rik	2,35	0,23	1,53	0,21	-1,54	0,012173008	0,075992051	66330
Zfp942	10,73	0,93	7,13	0,54	-1,54	8,24119E-13	2,14905E-11	73233
Tube1	4,21	0,34	2,84	0,54	-1,54	6,44873E-06	7,49812E-05	71924
Mmp3	5,49	0,49	3,62	0,69	-1,54	2,10727E-05	0,000226863	17392
Pnpla8	17,94	0,60	11,95	0,62	-1,54	0	0	67452
Nudt16	1,50	0,18	1,01	0,28	-1,54	0,004269098	0,02996553	75686
Mospd1	17,09	0,88	11,34	0,76	-1,54	9,86988E-14	2,82133E-12	70380
Rpl17-ps10	1,70	0,69	1,12	0,40	-1,54	0,147989395	0,590937499	100042880
Whamm	3,53	0,47	2,37	0,16	-1,54	9,81902E-06	0,000110873	434204
Mettl3	16,86	1,09	11,38	0,98	-1,54	8,57869E-13	2,23451E-11	56335
Syne3	13,71	1,99	9,18	1,01	-1,54	4,05559E-09	7,13862E-08	212073
Klhl11	11,60	0,95	7,74	0,56	-1,54	7,81952E-12	1,84559E-10	217194
Alkbh3	10,03	0,60	6,69	0,31	-1,55	6,39477E-12	1,51951E-10	69113
Snupn	7,98	0,40	5,29	0,77	-1,55	8,31578E-06	9,48798E-05	66069
Dennd4b	1,36	0,22	0,92	0,14	-1,55	2,8955E-05	0,000304277	229541
Calr	2127,49	177,85	1420,09	184,15	-1,55	3,07248E-10	6,1645E-09	12317
Brwd1	6,31	0,54	4,21	0,25	-1,55	1,11022E-16	3,99131E-15	93871
Slc2a1	21,41	1,88	14,27	0,71	-1,55	2,44249E-15	8,04177E-14	20525
Ddx18	43,46	2,20	29,01	1,44	-1,55	0	0	66942

AI413582	29,85	0,88	20,07	1,58	-1,55	5,05496E-12	1,21694E-10	106672
2410016O06Rik	7,09	0,20	4,65	0,57	-1,55	2,63014E-09	4,74265E-08	71952
Ascc2	23,31	1,00	15,60	0,83	-1,55	0	0	75452
Jun	19,85	0,86	13,25	0,13	-1,55	7,77156E-16	2,66217E-14	16476
Nek1	7,44	0,50	4,92	0,64	-1,55	5,36238E-14	1,57314E-12	18004
Rnf146	25,43	0,88	16,89	0,65	-1,55	0	0	68031
Rnaseh1	14,04	0,05	9,42	0,88	-1,55	2,15059E-10	4,41156E-09	19819
Tbc1d9	1,95	0,20	1,30	0,06	-1,55	7,66423E-06	8,80405E-05	71310
Zfp956	5,46	0,66	3,62	0,24	-1,55	6,42904E-09	1,10861E-07	101197
Mrpl32	40,18	1,07	26,68	3,00	-1,55	1,62914E-12	4,12148E-11	75398
Rasl11a	3,35	0,88	2,23	0,09	-1,55	0,004298838	0,030146537	68895
Tet2	1,65	0,13	1,10	0,12	-1,55	4,63329E-09	8,1118E-08	214133
Pfkfb2	3,23	0,37	2,13	0,16	-1,55	1,39888E-12	3,55468E-11	18640
Lrp1	80,19	3,37	53,17	15,21	-1,55	5,66086E-06	6,63939E-05	16971
Gm527	3,04	0,29	2,00	0,25	-1,56	0,000727881	0,006020508	217648
Cnksr3	4,75	0,34	3,15	0,09	-1,56	2,21721E-10	4,54007E-09	215748
Mrps2	23,24	0,69	15,42	0,23	-1,56	2,10942E-15	6,98537E-14	118451
Exoc6	7,20	0,62	4,73	0,56	-1,56	8,20155E-12	1,93277E-10	107371
Slmo1	1,98	0,15	1,32	0,19	-1,56	0,000146788	0,001378568	225655
Znhit6	10,35	0,46	6,83	0,46	-1,56	4,77063E-13	1,27379E-11	229937
Larp1b	3,51	0,28	2,31	0,10	-1,56	2,13163E-14	6,49494E-13	214048
Arpc5l	38,67	0,44	25,62	1,66	-1,56	0	0	74192
Rnf11	60,39	2,49	39,88	1,83	-1,56	0	0	29864
Thap1	5,16	0,18	3,41	0,08	-1,56	8,30924E-09	1,41629E-07	73754
Clpp	39,32	2,55	26,22	2,07	-1,56	6,99441E-15	2,21829E-13	53895
Trnt1	19,03	0,39	12,54	1,12	-1,56	0	0	70047
Riok1	15,27	0,72	10,09	0,31	-1,56	2,58682E-14	7,84531E-13	71340
Trpt1	4,58	0,47	2,99	0,32	-1,56	0,000333671	0,002934368	107328
Psph	37,54	1,68	24,86	0,41	-1,56	0	0	100678
Dnajc3	35,96	2,76	23,74	1,58	-1,56	0	0	100037258
Rft1	3,86	0,27	2,53	0,52	-1,56	5,01594E-07	6,77552E-06	328370
Lyst	1,85	0,21	1,21	0,11	-1,56	9,26326E-12	2,17402E-10	17101
Utp15	15,89	0,29	10,51	0,44	-1,56	2,22045E-16	7,82284E-15	105372
Zxdc	10,15	0,17	6,73	0,24	-1,56	1,11022E-16	3,99131E-15	80292
Zfp131	16,39	0,37	10,82	0,18	-1,56	1,11022E-16	3,99131E-15	72465
Ctgf	43,43	5,87	28,71	3,67	-1,56	9,47417E-10	1,79986E-08	14219
Mina	10,71	0,10	7,07	0,05	-1,56	2,42695E-13	6,6429E-12	67014
Rpl17-ps5	2,74	1,38	1,81	0,73	-1,56	0,093461854	0,419074763	100042067
Endov	5,67	0,42	3,78	0,25	-1,56	2,13718E-13	5,89205E-12	338371
Ttc17	10,37	0,51	6,82	0,31	-1,56	1,11022E-16	3,99131E-15	74569
Gm8615	4,69	0,40	3,05	0,45	-1,56	1,29469E-05	0,00014364	667410
Dpp7	113,45	1,39	74,83	1,48	-1,56	0	0	83768
Cwf19l1	5,88	0,41	3,89	0,27	-1,56	1,47882E-13	4,16994E-12	72502
Gpr137b	33,05	1,98	21,76	0,77	-1,56	0	0	83924
Avil	3,73	0,28	2,43	0,32	-1,56	2,52412E-09	4,55866E-08	11567
Rnf125	1,74	0,12	1,15	0,05	-1,56	1,3215E-05	0,000146437	67664

Arhgap39	4,26	0,19	2,83	0,24	-1,56	9,48657E-11	2,02875E-09	223666
Ddx59	9,83	0,44	6,46	0,40	-1,57	8,69971E-13	2,26217E-11	67997
2010315B03Rik	4,95	0,12	3,22	0,35	-1,57	9,5675E-09	1,6169E-07	630836
Tada2a	16,08	0,46	10,59	1,21	-1,57	1,21014E-14	3,77263E-13	217031
Tmed8	3,48	0,21	2,28	0,03	-1,57	7,80542E-12	1,84322E-10	382620
Tmem179b	11,40	1,24	7,56	1,22	-1,57	1,21229E-05	0,000134956	67706
Gm12338	2,04	0,91	1,37	0,56	-1,57	0,130856445	0,540652422	654359
Saraf	105,27	1,94	69,33	1,32	-1,57	0	0	67887
Ghitm	122,28	0,58	80,44	3,97	-1,57	0	0	66092
Ormdl3	31,45	0,17	20,68	1,00	-1,57	1,11022E-16	3,99131E-15	66612
Snx16	11,05	0,54	7,23	0,60	-1,57	9,40026E-13	2,42915E-11	74718
Parl	44,04	0,97	28,90	1,30	-1,57	3,33067E-16	1,16357E-14	381038
Nmnat3	1,84	0,18	1,20	0,02	-1,57	0,000369467	0,003224997	74080
Adpgk	21,81	0,70	14,40	1,20	-1,57	6,66134E-16	2,2956E-14	72141
Tfap4	4,58	0,26	3,01	0,15	-1,57	7,35745E-13	1,9307E-11	83383
Dip2c	6,40	0,45	4,20	0,07	-1,57	8,88178E-16	3,03566E-14	208440
Phf20l1	11,20	1,42	7,35	0,47	-1,57	0	0	239510
Nt5dc1	7,25	0,84	4,77	0,27	-1,57	1,4322E-07	2,09407E-06	319638
Slc4a11	7,16	0,03	4,69	0,15	-1,57	8,17157E-12	1,9267E-10	269356
Nup43	35,66	1,46	23,33	1,24	-1,57	3,21965E-15	1,04576E-13	69912
Intu	1,85	0,11	1,21	0,07	-1,57	7,52991E-09	1,29066E-07	380614
Cnppd1	37,06	0,37	24,32	0,53	-1,57	0	0	69171
Slc35b1	73,37	3,30	48,05	5,40	-1,57	0	0	110172
Atg13	21,05	0,23	13,81	0,31	-1,57	0	0	51897
Rad9a	70,00	3,48	46,05	1,70	-1,57	0	0	19367
Sclt1	6,17	0,72	4,03	0,57	-1,57	2,36775E-07	3,35377E-06	67161
Mamld1	3,89	0,38	2,56	0,23	-1,57	2,0755E-07	2,96736E-06	333639
Atg2b	7,05	0,32	4,62	0,17	-1,57	0	0	76559
Zfp316	4,27	0,11	2,82	0,19	-1,57	5,89084E-13	1,55475E-11	54201
Meox1	1,27	0,07	0,83	0,05	-1,57	0,004797187	0,033233916	17285
Rab24	26,73	2,30	17,48	0,70	-1,57	9,99201E-16	3,40241E-14	19336
Ahi1	4,63	0,17	3,04	0,04	-1,57	8,29881E-12	1,95067E-10	52906
Kcmf1	28,20	0,68	18,46	0,26	-1,57	0	0	74287
Gorab	12,57	0,65	8,18	0,87	-1,58	2,0095E-13	5,56015E-12	98376
Zfp715	4,99	0,40	3,24	0,09	-1,58	2,39351E-11	5,39866E-10	69930
Fitm2	7,63	0,99	5,00	0,54	-1,58	2,19769E-06	2,7161E-05	228859
Mtfr1	27,45	1,36	17,93	1,32	-1,58	0	0	67472
Zfp605	4,03	0,14	2,63	0,17	-1,58	3,76015E-11	8,30517E-10	675812
Gm33378	2,31	0,13	1,49	0,20	-1,58	6,79094E-06	7,87601E-05	102636264
Tinf2	10,53	0,69	6,96	0,64	-1,58	2,03171E-14	6,19874E-13	28113
Mmp11	33,42	2,62	21,93	1,03	-1,58	0	0	17385
Gm10171	18,65	4,57	12,19	3,15	-1,58	0,000111905	0,001071183	100041219
Spry2	4,99	0,38	3,28	0,12	-1,58	4,67926E-10	9,20267E-09	24064
Cbx4	4,50	0,16	2,94	0,19	-1,58	8,03757E-12	1,89608E-10	12418
Rhpn1	1,47	0,29	0,98	0,13	-1,58	0,002504844	0,018516262	14787
Mios	8,10	0,13	5,26	0,57	-1,58	1,48992E-13	4,19349E-12	252875

Eif3c	240,28	8,19	157,01	4,01	-1,58	0	0	56347
Nr4a1	1,80	0,14	1,18	0,01	-1,58	1,10055E-05	0,000123296	15370
Tab2	42,39	0,57	27,66	0,38	-1,58	0	0	68652
Dgke	4,47	0,26	2,93	0,08	-1,58	5,67724E-12	1,35676E-10	56077
Smpd5	1,59	0,20	1,03	0,21	-1,58	0,001549399	0,011963602	100503915
Atg16l2	1,20	0,03	0,78	0,05	-1,58	3,60357E-05	0,000372358	73683
Yae1d1	7,86	0,56	5,10	0,46	-1,58	1,05704E-12	2,71926E-11	67008
Kdm4c	7,77	0,63	5,05	0,21	-1,58	1,9984E-14	6,10526E-13	76804
Blzf1	6,01	0,51	3,91	0,29	-1,58	3,28115E-12	8,07757E-11	66352
Mrpl47	19,56	1,28	12,82	1,22	-1,58	3,42155E-10	6,8319E-09	74600
Acp6	12,19	1,29	7,95	0,62	-1,58	9,29937E-08	1,38944E-06	66659
Dnajc21	24,24	0,71	15,70	1,21	-1,58	4,44089E-16	1,54436E-14	78244
Epha2	23,90	2,42	15,58	1,19	-1,59	0	0	13836
Derl2	12,62	0,71	8,15	0,57	-1,59	1,11022E-16	3,99131E-15	116891
Bbc3	7,74	0,32	5,03	0,22	-1,59	3,87468E-13	1,0437E-11	170770
A430005L14Rik	29,84	2,98	19,40	1,07	-1,59	5,41789E-14	1,58739E-12	97159
Krr1	21,11	1,41	13,68	0,97	-1,59	0	0	52705
Afap1	9,74	0,39	6,36	0,42	-1,59	0	0	70292
AA467197	1,33	0,02	0,86	0,16	-1,59	0,042259755	0,221714739	433470
Fbxo32	10,60	0,69	6,90	0,47	-1,59	0	0	67731
Pla2g12a	23,00	0,54	14,89	0,50	-1,59	3,33067E-15	1,08028E-13	66350
Shq1	4,51	0,39	2,92	0,27	-1,59	1,19994E-09	2,24787E-08	72171
Hsp90b1	1783,17	225,29	1155,54	162,91	-1,59	1,77472E-11	4,05696E-10	22027
Glce	28,46	1,03	18,41	0,37	-1,59	0	0	93683
1700003E16Rik	1,29	0,29	0,83	0,18	-1,59	0,011162667	0,070366319	71837
Dcp1b	1,90	0,05	1,24	0,27	-1,59	2,57291E-05	0,000273275	319618
Atxn2l	36,79	1,04	23,87	1,24	-1,59	0	0	233871
Tmem220	1,58	0,18	1,00	0,15	-1,59	0,00725727	0,048099775	338369
Adamts6	2,89	0,21	1,90	0,26	-1,60	4,86395E-08	7,53295E-07	108154
Tra2b	77,84	2,13	50,25	2,71	-1,60	0	0	20462
Ncoa4	3,74	1,11	2,41	0,35	-1,60	5,65955E-05	0,000569001	27057
Brinp1	4,02	0,16	2,64	0,36	-1,60	2,87592E-09	5,15739E-08	56710
Acsf3	10,06	1,14	6,50	0,34	-1,60	1,13265E-12	2,90722E-11	257633
Taf1b	9,83	0,55	6,25	0,66	-1,60	1,07192E-12	2,75598E-11	21340
Zscan25	12,73	1,20	8,30	0,76	-1,60	2,14939E-13	5,91859E-12	666311
Tfam	24,82	0,64	15,95	0,76	-1,60	0	0	21780
Plin4	7,25	0,30	4,69	0,42	-1,60	1,11022E-16	3,99131E-15	57435
Zeb1	6,64	0,34	4,28	0,17	-1,60	1,11022E-16	3,99131E-15	21417
Zfp36l1	30,89	0,99	19,95	0,70	-1,60	0	0	12192
Srgap3	7,69	0,17	4,96	0,54	-1,60	0	0	259302
Lrrfip2	10,18	0,17	6,57	0,14	-1,60	0	0	71268
Rps23-ps2	1,16	0,21	0,69	0,38	-1,60	0,16488538	0,637114101	100503854
Lpin3	3,02	0,16	1,97	0,21	-1,60	1,57972E-09	2,91633E-08	64899
Tmem209	45,25	1,02	29,09	2,37	-1,60	0	0	72649
Aasdh	1,48	0,04	0,94	0,20	-1,60	1,66259E-06	2,09034E-05	231326
Arl13b	12,05	0,45	7,70	0,75	-1,60	2,22045E-16	7,82284E-15	68146

Mef2a	20,87	0,83	13,40	0,41	-1,60	0	0	17258
Mthfd1l	47,33	3,02	30,46	1,71	-1,60	0	0	270685
Siah1a	5,80	0,35	3,72	0,19	-1,61	1,34337E-14	4,17092E-13	20437
Pstk	6,76	0,71	4,37	0,08	-1,61	3,15867E-06	3,8354E-05	214580
Zfp593	9,60	0,11	6,19	0,81	-1,61	7,76045E-08	1,17059E-06	68040
Scaf8	27,73	0,39	17,83	0,05	-1,61	0	0	106583
Ift52	16,50	0,71	10,54	0,54	-1,61	2,77556E-15	9,0991E-14	245866
Rel	5,62	0,14	3,65	0,96	-1,61	3,64295E-07	5,01261E-06	19696
Tifa	5,10	0,14	3,25	0,23	-1,61	1,73576E-10	3,59443E-09	211550
Fam221a	1,47	0,31	0,95	0,06	-1,61	0,002652114	0,019522924	231946
Arhgap12	19,76	0,98	12,66	0,56	-1,61	0	0	75415
1110038B12Rik	60,06	1,50	38,52	1,11	-1,61	0	0	68763
Polg2	2,69	0,21	1,71	0,13	-1,61	8,42518E-05	0,000825119	50776
Kansl1l	2,64	0,10	1,69	0,14	-1,61	2,22045E-16	7,82284E-15	68691
Irak1bp1	16,23	1,58	10,44	0,14	-1,61	3,36398E-14	1,00886E-12	65099
Pdpr	10,93	1,22	7,00	0,22	-1,61	0	0	319518
Utp18	24,08	0,70	15,38	0,94	-1,61	0	0	217109
Lif	4,21	0,46	2,72	0,20	-1,61	1,40776E-13	3,97939E-12	16878
Cep131	5,53	0,29	3,56	0,02	-1,61	5,45786E-13	1,45136E-11	12009
Snx5	81,10	2,12	51,91	2,52	-1,61	0	0	69178
BC048403	7,42	0,15	4,74	0,34	-1,61	3,59712E-13	9,7065E-12	270802
Slco2a1	12,50	0,54	8,06	0,74	-1,61	0	0	24059
Gtpbp3	10,84	0,73	7,00	0,88	-1,61	7,99361E-15	2,51947E-13	70359
Insl6	3,61	0,34	2,29	0,17	-1,61	0,007591719	0,05005547	27356
C430049B03Rik	1,30	0,07	0,84	0,08	-1,61	0,000117511	0,001122028	72575
Sesn2	29,58	1,16	18,98	0,95	-1,61	0	0	230784
Tcea1	159,59	2,92	102,15	2,98	-1,61	0	0	21399
Gm9260	1,78	0,25	1,11	0,68	-1,61	0,037083064	0,19823694	668595
Slc37a4	4,47	0,31	2,89	0,26	-1,61	4,22799E-09	7,43636E-08	14385
Taf1a	5,13	0,27	3,27	0,17	-1,61	2,97879E-09	5,32725E-08	21339
Reps1	28,45	0,65	18,23	0,48	-1,61	0	0	19707
Traf1	1,25	0,26	0,81	0,01	-1,61	3,88385E-05	0,000398888	22029
Nt5dc3	14,79	0,76	9,48	0,16	-1,61	0	0	103466
Ears2	1,78	0,14	1,13	0,07	-1,62	8,07424E-09	1,37931E-07	67417
Maml2	3,81	0,01	2,43	0,11	-1,62	2,34812E-13	6,43869E-12	270118
Gm5173	1,13	0,23	0,74	0,30	-1,62	0,065710659	0,318261012	382388
Ing3	16,83	0,62	10,74	0,23	-1,62	0	0	71777
Zmym6	11,58	0,70	7,36	0,56	-1,62	0	0	100177
Gm13033	1,22	0,28	0,77	0,07	-1,62	0,019927444	0,116681101	100038703
Odc1	158,83	6,45	101,37	2,55	-1,62	0	0	18263
Cdc42se2	39,43	1,14	25,11	1,07	-1,62	0	0	72729
Wsb1	43,73	2,96	27,90	2,29	-1,62	0	0	78889
Zfp287	3,89	0,17	2,46	0,14	-1,62	1,10578E-13	3,14715E-12	170740
Ascc3	18,33	0,88	11,67	0,08	-1,62	0	0	77987
Mks1	1,15	0,17	0,73	0,09	-1,62	0,002075558	0,01562301	380718
Vps39	11,24	0,59	7,14	0,16	-1,62	0	0	269338

Mtrr	11,34	0,28	7,20	0,28	-1,62	0	0	210009
Gm20585	1,05	0,42	0,68	0,09	-1,62	0,125094076	0,521728826	100416786
Ankrd12	12,20	1,09	7,77	0,26	-1,62	0	0	106585
Gm33804	4,58	0,21	2,92	0,50	-1,62	0,00189281	0,014334677	102636848
Gm9079	2,08	0,27	1,29	0,30	-1,62	0,001255577	0,009896912	668272
Hbegf	21,59	2,40	13,75	0,37	-1,62	0	0	15200
Gm10509	5,24	0,68	3,30	0,37	-1,62	1,6721E-06	2,10115E-05	100310809
Slc25a36	11,81	1,13	7,51	0,29	-1,62	0	0	192287
Zfp729a	5,30	0,54	3,36	0,40	-1,62	1,72085E-14	5,29264E-13	212281
LOC105244345	1,01	0,55	0,60	0,43	-1,62	0,228052445	0,783133501	105244345
Akap8l	20,75	1,62	13,18	0,66	-1,62	1,11022E-16	3,99131E-15	54194
Pde3b	7,04	0,18	4,46	0,18	-1,62	0	0	18576
6430548M08Rik	9,22	0,48	5,86	0,21	-1,62	0	0	234797
Uhrf1bp1l	11,10	0,10	7,05	0,13	-1,62	0	0	75089
Gnpda1	74,12	2,30	47,06	5,52	-1,62	0	0	26384
Pde1a	4,56	0,27	2,92	0,33	-1,62	1,56541E-14	4,82757E-13	18573
Nog	1,21	0,15	0,77	0,29	-1,62	0,015727111	0,09510455	18121
Prune2	27,88	2,19	17,71	2,17	-1,62	5,14078E-12	1,23635E-10	353211
Dlc1	4,22	0,22	2,69	0,19	-1,63	0	0	50768
Nepro	6,07	0,20	3,84	0,59	-1,63	7,21645E-15	2,28396E-13	212547
Coq10b	18,94	0,53	11,88	1,26	-1,63	3,44169E-15	1,11235E-13	67876
E130317F20Rik	3,46	0,07	2,17	0,46	-1,63	4,87917E-07	6,60051E-06	414101
Taf15	84,95	1,52	53,75	2,34	-1,63	0	0	70439
Snx33	13,66	1,17	8,66	0,17	-1,63	0	0	235406
Rdh14	9,28	0,78	5,86	0,87	-1,63	1,7661E-07	2,55292E-06	105014
Zfp62	10,20	0,24	6,42	0,51	-1,63	0	0	22720
Ric8a	17,58	1,84	11,16	0,95	-1,63	0	0	101489
Dbt	6,18	0,19	3,87	0,43	-1,63	2,08567E-08	3,40045E-07	13171
Slc7a3	36,14	0,70	22,78	1,70	-1,63	0	0	11989
Alyref2	2,48	0,39	1,61	0,19	-1,63	0,001951107	0,014727433	56009
Tmem120b	6,64	0,24	4,21	0,04	-1,63	5,82012E-10	1,13103E-08	330189
Ssc4d	2,41	0,02	1,50	0,22	-1,63	1,86749E-05	0,000202573	109267
Plagl1	3,89	0,11	2,44	0,14	-1,63	2,25242E-12	5,62061E-11	22634
B9d2	4,22	0,73	2,71	0,40	-1,63	8,04698E-06	9,20445E-05	232987
Riok2	20,71	1,18	13,09	0,49	-1,63	0	0	67045
Lrrc49	9,94	0,57	6,24	0,80	-1,63	3,21965E-15	1,04576E-13	102747
B3galt6	3,36	0,40	2,12	0,32	-1,63	9,97005E-07	1,29148E-05	117592
Diexf	4,58	0,43	2,89	0,19	-1,63	6,39488E-14	1,86409E-12	215193
Mettl14	18,10	1,02	11,37	1,20	-1,63	0	0	210529
Lrrfip1	34,00	2,26	21,48	1,00	-1,63	0	0	16978
Pnpo	11,38	0,53	7,10	0,73	-1,64	3,04312E-13	8,27993E-12	103711
9330151L19Rik	1,50	0,16	0,96	0,20	-1,64	0,000426433	0,003681516	414085
Repin1	10,71	0,49	6,75	0,41	-1,64	1,11022E-15	3,76366E-14	58887
Rpsa-ps7	1,04	0,46	0,65	0,28	-1,64	0,110532726	0,476245896	433141
Usp6nl	8,19	0,36	5,16	0,30	-1,64	0	0	98910
Zfp708	1,93	0,24	1,19	0,17	-1,64	9,84825E-05	0,000952865	432769

Nsun4	6,11	0,63	3,84	0,25	-1,64	9,20852E-09	1,55854E-07	72181
Tars2	10,69	0,53	6,73	0,26	-1,64	0	0	71807
Tbcc	13,35	0,93	8,40	0,96	-1,64	9,80327E-14	2,80579E-12	72726
Ggnbp2	47,64	0,41	29,98	0,38	-1,64	0	0	217039
Ltv1	37,67	1,48	23,70	1,27	-1,64	0	0	353258
Elovl4	13,64	0,36	8,53	0,75	-1,64	0	0	83603
Jak2	9,86	0,40	6,21	0,03	-1,64	0	0	16452
Yipf2	13,60	1,31	8,51	1,63	-1,64	1,56399E-09	2,88962E-08	74766
Tsr3	34,54	1,92	21,74	0,28	-1,64	0	0	68327
Hscb	12,81	0,41	8,06	0,67	-1,64	8,89322E-07	1,16053E-05	100900
Abca5	8,31	0,07	5,23	0,18	-1,64	0	0	217265
Smndc1	25,46	0,55	15,93	0,80	-1,64	0	0	76479
Tmem126a	63,13	0,38	39,64	0,82	-1,64	0	0	66271
Zfp383	3,61	0,16	2,27	0,14	-1,64	7,37316E-07	9,71868E-06	73729
Zfp97	10,71	1,31	6,72	1,86	-1,64	5,95719E-08	9,13031E-07	22759
Slc17a5	9,55	0,34	5,98	0,29	-1,64	2,22045E-16	7,82284E-15	235504
1500011K16Rik	2,75	0,35	1,79	0,46	-1,64	0,003369936	0,024169827	67885
Mtr	3,73	0,35	2,33	0,43	-1,64	1,02632E-09	1,93849E-08	238505
Zfp451	9,23	0,54	5,78	0,08	-1,64	0	0	98403
Gm5088	1,31	0,27	0,81	0,16	-1,64	0,000986774	0,007938078	328451
Tmem185b	8,28	0,09	5,19	0,24	-1,64	1,43552E-13	4,05534E-12	226351
Zfp280d	7,36	0,42	4,61	0,12	-1,65	0	0	235469
Ube2g2	62,19	3,53	39,06	2,01	-1,65	0	0	22213
Dgkd	11,32	0,37	7,09	0,22	-1,65	0	0	227333
Prickle2	1,92	0,08	1,20	0,17	-1,65	3,06623E-08	4,88431E-07	243548
Zfp869	17,83	1,08	11,17	0,17	-1,65	0	0	66869
Ttc26	4,63	0,40	2,88	0,11	-1,65	1,54521E-12	3,91564E-11	264134
Spty2d1	10,06	0,34	6,27	0,26	-1,65	0	0	101685
Dusp3	32,29	0,55	20,16	0,77	-1,65	0	0	72349
Gm7993	23,11	1,92	14,55	1,37	-1,65	7,77156E-16	2,66217E-14	666231
Ctns	21,88	2,40	13,63	0,81	-1,65	0	0	83429
Gm44504	5,77	1,83	3,62	0,35	-1,65	0,000521246	0,004427548	100169864
Rae1	37,37	0,65	23,33	0,56	-1,65	0	0	66679
2700062C07Rik	17,77	0,71	11,09	1,18	-1,65	3,39406E-12	8,34207E-11	68046
Gm18234	1,81	0,39	1,12	0,37	-1,65	0,02311357	0,132721774	100416762
Pign	11,36	0,22	7,08	0,25	-1,65	0	0	27392
Skil	7,86	0,20	4,91	0,09	-1,65	0	0	20482
Irf2bp2	16,96	1,24	10,62	0,68	-1,65	0	0	270110
Birc3	8,35	0,36	5,19	0,33	-1,65	1,9984E-15	6,64175E-14	11796
Lrrc61	2,56	0,18	1,58	0,12	-1,65	5,43516E-06	6,38778E-05	243371
BC029214	9,04	0,47	5,64	0,28	-1,66	1,28653E-11	2,98267E-10	227622
Kctd18	2,49	0,32	1,55	0,03	-1,66	1,71396E-07	2,48068E-06	51960
Slc29a2	2,79	0,33	1,76	0,23	-1,66	3,60696E-06	4,34515E-05	13340
Irf7	5,21	0,51	3,28	0,19	-1,66	7,59601E-07	9,99802E-06	54123
Kdm4a	28,16	1,02	17,57	1,17	-1,66	0	0	230674
Erlin1	32,93	0,48	20,46	0,45	-1,66	0	0	226144

**Brox**	**39,17**	**0,71**	**24,36**	**0,25**	**-1,66**	**0**	**0**	**71678**
**Neil1**	**3,02**	**0,13**	**1,84**	**0,36**	**-1,66**	**1,02818E-05**	**0,00011575**	**72774**
**Tcta**	**8,71**	**0,65**	**5,47**	**0,81**	**-1,66**	**2,9517E-08**	**4,72049E-07**	**102791**
**Mri1**	**16,03**	**0,25**	**9,98**	**0,21**	**-1,66**	**1,11022E-16**	**3,99131E-15**	**67873**
**Mthfr**	**18,57**	**0,47**	**11,58**	**0,66**	**-1,66**	**0**	**0**	**17769**
**Zfp850**	**2,05**	**0,09**	**1,26**	**0,10**	**-1,66**	**6,02425E-11**	**1,30663E-09**	**100043772**
**Wdr54**	**4,93**	**0,30**	**3,07**	**0,37**	**-1,66**	**2,78471E-06**	**3,40572E-05**	**75659**
**Pygo1**	**1,24**	**0,14**	**0,77**	**0,04**	**-1,66**	**0,00655241**	**0,043834389**	**72135**
**Eme2**	**10,80**	**0,51**	**6,66**	**0,92**	**-1,66**	**9,22973E-12**	**2,16726E-10**	**193838**
**Arap2**	**1,42**	**0,07**	**0,87**	**0,13**	**-1,66**	**3,72643E-07**	**5,11365E-06**	**212285**
**Yy2**	**4,44**	**0,25**	**2,73**	**0,16**	**-1,66**	**3,61704E-10**	**7,20965E-09**	**100073351**
**Supt3**	**5,84**	**0,19**	**3,60**	**0,25**	**-1,66**	**2,59792E-14**	**7,86856E-13**	**109115**
**Ggnbp1**	**2,02**	**0,13**	**1,27**	**0,22**	**-1,67**	**0,000376637**	**0,003281323**	**70772**
**Elac1**	**2,05**	**0,33**	**1,26**	**0,07**	**-1,67**	**6,01444E-07**	**8,03652E-06**	**114615**
**Rragd**	**2,93**	**0,13**	**1,79**	**0,28**	**-1,67**	**3,27602E-11**	**7,28156E-10**	**52187**
**Dhtkd1**	**1,91**	**0,03**	**1,21**	**0,25**	**-1,67**	**8,82787E-08**	**1,32331E-06**	**209692**
**Gm6505**	**6,02**	**0,53**	**3,69**	**0,31**	**-1,67**	**2,71933E-07**	**3,81983E-06**	**624446**
**Snhg7**	**2,97**	**0,41**	**1,86**	**0,56**	**-1,67**	**3,48414E-05**	**0,000360669**	**72091**
**Slc35c2**	**21,54**	**0,88**	**13,27**	**0,51**	**-1,67**	**0**	**0**	**228875**
**Pigw**	**4,25**	**0,13**	**2,57**	**0,43**	**-1,67**	**2,0408E-07**	**2,92049E-06**	**70325**
**Gm4419**	**1,78**	**0,47**	**1,09**	**0,05**	**-1,67**	**0,000178698**	**0,00165281**	**100043407**
**Nadsyn1**	**2,53**	**0,18**	**1,57**	**0,10**	**-1,67**	**6,90722E-06**	**7,99668E-05**	**78914**
**Smurf1**	**26,56**	**1,78**	**16,42**	**1,11**	**-1,67**	**0**	**0**	**75788**
**Ndufaf6**	**3,44**	**0,38**	**2,06**	**0,53**	**-1,67**	**0,000131855**	**0,001248317**	**76947**
**Smad1**	**15,12**	**0,08**	**9,33**	**0,15**	**-1,67**	**0**	**0**	**17125**
**Tmem17**	**2,19**	**0,27**	**1,33**	**0,27**	**-1,67**	**0,002147689**	**0,016087581**	**103765**
**Fam135a**	**7,04**	**0,41**	**4,33**	**0,13**	**-1,67**	**0**	**0**	**68187**
**Eps8**	**17,96**	**0,34**	**11,09**	**0,44**	**-1,68**	**0**	**0**	**13860**
**Tsc22d3**	**14,64**	**0,53**	**9,04**	**0,25**	**-1,68**	**0**	**0**	**14605**
**Trub2**	**12,55**	**0,51**	**7,71**	**0,30**	**-1,68**	**0**	**0**	**227682**
**Rspo2**	**3,68**	**0,11**	**2,27**	**0,21**	**-1,68**	**1,40327E-11**	**3,24456E-10**	**239405**
**Slc16a1**	**31,61**	**0,60**	**19,44**	**0,39**	**-1,68**	**0**	**0**	**20501**
**Zfp28**	**6,78**	**0,60**	**4,18**	**0,17**	**-1,68**	**6,66134E-16**	**2,2956E-14**	**22690**
**Mycl**	**2,70**	**0,08**	**1,67**	**0,09**	**-1,68**	**3,31118E-07**	**4,58644E-06**	**16918**
**Gm9892**	**2,44**	**0,87**	**1,48**	**0,25**	**-1,68**	**0,003731058**	**0,02651015**	**100040220**
**Dync1li1**	**40,06**	**2,43**	**24,58**	**1,49**	**-1,68**	**0**	**0**	**235661**
**Col12a1**	**13,98**	**0,33**	**8,57**	**2,04**	**-1,68**	**3,47278E-13**	**9,39312E-12**	**12816**
**Trio**	**12,11**	**0,31**	**7,43**	**0,99**	**-1,68**	**0**	**0**	**223435**
**Ndufaf4**	**6,88**	**0,77**	**4,18**	**0,58**	**-1,68**	**6,39974E-10**	**1,23736E-08**	**68493**
**Sema7a**	**1,99**	**0,29**	**1,23**	**0,16**	**-1,68**	**3,41361E-05**	**0,000353768**	**20361**
**Fbn1**	**34,31**	**1,58**	**21,01**	**4,80**	**-1,68**	**1,24678E-13**	**3,53525E-12**	**14118**
**Nupl2**	**4,47**	**0,20**	**2,74**	**0,04**	**-1,69**	**1,08449E-10**	**2,30202E-09**	**231042**
**Asxl2**	**7,72**	**0,56**	**4,73**	**0,27**	**-1,69**	**0**	**0**	**75302**
**Zfp9**	**11,80**	**0,42**	**7,21**	**0,19**	**-1,69**	**0**	**0**	**22750**
**Ino80**	**12,48**	**0,68**	**7,64**	**0,28**	**-1,69**	**0**	**0**	**68142**
**Cd274**	**1,82**	**0,06**	**1,11**	**0,14**	**-1,69**	**6,91522E-06**	**8,00392E-05**	**60533**

Atmin	9,29	0,51	5,66	0,38	-1,69	0	0	234776
Lcorl	1,91	0,22	1,17	0,05	-1,69	0	0	209707
Setdb2	4,95	0,61	3,01	0,29	-1,69	1,9976E-08	3,26268E-07	239122
BC003331	18,37	1,07	11,17	0,99	-1,69	0	0	226499
Jrkl	6,74	0,27	4,06	0,44	-1,69	1,30007E-13	3,68179E-12	77532
Zfp263	8,80	0,26	5,38	0,14	-1,69	0	0	74120
Pggt1b	16,20	0,81	9,88	0,26	-1,69	0	0	225467
Rif1	12,90	0,61	7,87	0,27	-1,69	0	0	51869
Zfp518a	2,51	0,26	1,52	0,12	-1,69	1,64594E-09	3,03003E-08	72672
Ankrd11	41,81	1,08	25,51	2,62	-1,69	0	0	77087
Fancf	4,27	0,45	2,57	0,19	-1,69	3,45487E-06	4,17513E-05	100040608
Tnnc1	2,56	0,49	1,50	0,69	-1,69	0,024401339	0,139069399	21924
Bend3	2,98	0,05	1,82	0,08	-1,69	0	0	331623
Gm10231	16,11	12,52	9,81	1,67	-1,69	0,016437662	0,098901445	100040342
Eif1a	73,47	1,84	44,74	0,30	-1,69	0	0	13664
Zfp661	1,02	0,09	0,62	0,03	-1,70	1,15462E-06	1,48055E-05	72180
Gm6451	1,75	0,52	1,05	0,29	-1,70	0,038061198	0,202873367	623804
Mphosph10	23,60	0,98	14,27	0,43	-1,70	0	0	67973
4933434E20Rik	20,53	1,73	12,42	0,51	-1,70	0	0	99650
Iqcb1	5,18	0,19	3,11	0,32	-1,70	0	0	320299
Nrip1	2,27	0,07	1,38	0,06	-1,70	0	0	268903
Cdk17	6,29	0,06	3,79	0,33	-1,70	0	0	237459
Dnase2a	35,11	1,65	21,41	1,20	-1,70	0	0	13423
Slc43a3	17,36	0,56	10,52	0,65	-1,70	0	0	58207
Ubr4	20,51	1,82	12,43	2,69	-1,70	4,69624E-14	1,3866E-12	69116
Rbm39	49,12	1,35	29,80	2,33	-1,70	0	0	170791
Nkd2	1,63	0,07	1,00	0,13	-1,70	5,25947E-06	6,19402E-05	72293
Tom1l1	10,42	0,33	6,24	0,75	-1,70	7,66054E-15	2,42116E-13	71943
LOC108167946	1,56	0,52	0,97	0,31	-1,70	0,045152403	0,233667237	108167946
Zxda	1,50	0,12	0,90	0,10	-1,70	1,71696E-07	2,48424E-06	668171
Zfp119b	4,18	0,12	2,53	0,08	-1,70	6,16174E-13	1,62438E-11	240120
Gtf2h1	40,15	0,52	24,30	0,40	-1,70	0	0	14884
Gm30124	1,54	0,46	0,92	0,06	-1,70	0,013098682	0,081095942	102631909
Glis1	1,60	0,28	0,98	0,23	-1,70	0,000124084	0,001181344	230587
Phkg2	10,23	0,71	6,23	0,57	-1,71	6,93889E-14	2,01369E-12	68961
Gm21168	2,43	0,02	1,48	0,13	-1,71	3,54276E-07	4,88569E-06	100861728
Luc7l	22,01	0,54	13,32	0,16	-1,71	0	0	66978
Ccdc174	17,28	0,46	10,40	0,35	-1,71	0	0	232236
Rpl5-ps2	1,96	0,44	1,21	0,43	-1,71	0,015536027	0,094148077	668936
Kri1	20,97	1,61	12,71	0,42	-1,71	0	0	215194
Rbsn	19,59	0,09	11,79	0,68	-1,71	0	0	78287
Elmod2	5,63	0,18	3,36	0,44	-1,71	1,11022E-16	3,99131E-15	244548
Mdn1	6,75	0,82	4,08	0,80	-1,71	1,58096E-13	4,43065E-12	100019
Soat1	21,52	0,62	12,97	0,32	-1,71	0	0	20652
Madd	6,23	0,18	3,75	0,22	-1,71	0	0	228355
Gm15801	9,94	1,01	5,98	0,66	-1,71	1,11022E-16	3,99131E-15	627166

Rcbtb1	17,06	0,72	10,28	0,45	-1,71	0	0	71330
Ifi44	2,39	0,46	1,47	0,21	-1,71	2,19525E-05	0,000236001	99899
Clcn7	36,78	1,70	22,26	1,73	-1,71	0	0	26373
D030028A08Rik	1,11	0,16	0,67	0,04	-1,71	3,15434E-10	6,3149E-09	319371
Erdr1_1	2,63	0,18	1,58	0,05	-1,71	6,88338E-15	2,18611E-13	170942
Malat1	56,87	19,54	34,26	6,01	-1,71	1,9499E-06	2,42493E-05	72289
Ccdc57	1,81	0,10	1,07	0,12	-1,71	5,32774E-07	7,16498E-06	71276
Thumpd3	23,27	0,92	13,93	0,78	-1,71	0	0	14911
Dtwd1	15,39	0,41	9,30	0,46	-1,72	2,22045E-16	7,82284E-15	69185
Med13l	12,30	0,21	7,41	1,05	-1,72	0	0	76199
Tmem201	7,51	0,48	4,55	0,30	-1,72	0	0	230917
Arl4a	5,59	0,57	3,34	0,73	-1,72	4,87072E-10	9,55458E-09	11861
Slc18a2	1,58	0,37	0,94	0,12	-1,72	0,000100989	0,000975265	214084
Zbtb49	1,24	0,15	0,75	0,03	-1,72	1,5405E-07	2,24383E-06	75079
Tlk2	16,01	0,42	9,60	0,33	-1,72	0	0	24086
Nlrc3	1,10	0,19	0,65	0,04	-1,72	9,44164E-08	1,40932E-06	268857
Chm	16,11	0,23	9,63	1,36	-1,72	0	0	12662
Bend6	6,41	0,57	3,81	0,40	-1,72	2,27152E-13	6,23985E-12	320705
Rest	9,35	0,52	5,61	0,29	-1,72	0	0	19712
Pcgf1	11,34	0,90	6,77	0,42	-1,72	1,44978E-07	2,1191E-06	69837
Zfp37	3,47	0,45	2,06	0,25	-1,72	1,03048E-08	1,73446E-07	22696
Mettl1	26,66	1,28	15,97	1,96	-1,72	0	0	17299
Mafk	9,81	0,56	5,90	0,24	-1,72	0	0	17135
Ccdc122	9,99	1,32	5,93	0,75	-1,72	1,22532E-09	2,29341E-08	108811
Mcoln1	67,44	0,54	40,34	4,03	-1,72	0	0	94178
Zscan26	13,62	0,80	8,09	0,40	-1,73	0	0	432731
Tbcel	9,84	0,39	5,87	0,12	-1,73	0	0	272589
Tmem230	11,74	1,08	6,94	0,65	-1,73	1,11022E-16	3,99131E-15	70612
Ring1	7,91	0,22	4,73	0,08	-1,73	5,7121E-13	1,51106E-11	19763
Slc2a6	10,80	1,11	6,52	1,35	-1,73	1,85777E-10	3,83323E-09	227659
Aldh1l2	121,14	2,61	72,36	2,35	-1,73	0	0	216188
Strbp	2,05	0,20	1,23	0,05	-1,73	0	0	20744
Zfp142	14,17	0,14	8,51	0,62	-1,73	0	0	77264
Smad7	5,91	0,19	3,52	0,19	-1,73	1,11022E-16	3,99131E-15	17131
Tmem202	2,02	0,15	1,21	0,17	-1,73	0,001368053	0,010698725	73893
Hspbap1	3,63	0,19	2,16	0,05	-1,73	0	0	66667
Ctage5	26,26	1,17	15,59	1,08	-1,73	0	0	217615
Uso1	43,76	0,86	26,05	0,99	-1,73	0	0	56041
Artn	1,03	0,10	0,62	0,07	-1,73	8,81551E-05	0,000860952	11876
Tmem194b	4,14	0,21	2,48	0,37	-1,73	1,31195E-12	3,34866E-11	227094
Helz2	4,78	0,18	2,86	0,27	-1,73	0	0	229003
Gm4767	3,44	0,75	2,05	0,25	-1,73	1,35064E-05	0,000149413	210583
Dph5	8,83	0,24	5,26	0,19	-1,73	2,36111E-12	5,87582E-11	69740
Eml2	3,38	0,41	2,00	0,44	-1,73	2,95429E-08	4,72242E-07	72205
Shmt2	301,73	29,66	179,51	10,78	-1,74	0	0	108037
Trim46	8,09	0,56	4,86	0,36	-1,74	0	0	360213

Cox18	8,77	0,79	5,19	0,25	-1,74	2,6465E-08	4,25271E-07	231430
Zkscan8	5,15	0,31	3,04	0,14	-1,74	0	0	93681
9130008F23Rik	3,15	0,44	1,90	0,23	-1,74	1,80788E-06	2,25874E-05	71583
Spata13	1,39	0,18	0,83	0,06	-1,74	2,96947E-09	5,31473E-08	219140
Myo1h	1,96	0,15	1,18	0,21	-1,74	8,58493E-08	1,28858E-06	231646
Cog3	14,12	0,47	8,37	0,31	-1,74	0	0	338337
Abcc1	24,58	0,91	14,64	1,24	-1,74	0	0	17250
Fn1	528,28	13,91	313,19	52,03	-1,74	1,33227E-15	4,48648E-14	14268
Rrp12	12,26	0,79	7,26	0,62	-1,74	0	0	107094
Bhlhb9	14,02	0,26	8,25	0,70	-1,74	0	0	70237
Clk4	11,57	0,42	6,82	0,35	-1,74	0	0	12750
Parp8	4,47	0,21	2,66	0,17	-1,74	0	0	52552
Apobr	5,69	0,20	3,38	0,22	-1,74	2,10942E-15	6,98537E-14	171504
Cdk10	22,07	1,15	13,04	0,67	-1,74	0	0	234854
Lars	130,15	1,57	77,13	3,20	-1,74	0	0	107045
H2-Q4	4,55	0,66	2,75	0,76	-1,74	1,14116E-05	0,000127477	15015
Lrp12	7,52	0,38	4,40	0,55	-1,74	0	0	239393
Tom1	6,34	2,60	3,74	0,59	-1,74	9,73153E-05	0,000942768	21968
Gm18095	1,11	0,22	0,66	0,30	-1,74	0,085909697	0,392890704	100416391
Rsrc2	46,41	0,92	27,37	2,28	-1,74	0	0	208606
Kif7	1,86	0,19	1,12	0,10	-1,74	7,47143E-08	1,1296E-06	16576
Zkscan6	4,50	0,43	2,68	0,10	-1,74	3,03568E-12	7,48535E-11	52712
Runx1	9,41	0,08	5,56	0,33	-1,75	0	0	12394
Pkp2	13,28	0,43	7,83	1,06	-1,75	0	0	67451
Fbxl12	3,90	0,30	2,31	0,10	-1,75	5,13938E-10	1,00386E-08	30843
Emx2	2,06	0,09	1,22	0,06	-1,75	7,74648E-06	8,88738E-05	13797
Gm10336	2,38	0,32	1,43	0,43	-1,75	1,12225E-05	0,000125605	328186
Fgfr2	1,94	0,02	1,15	0,14	-1,75	7,24539E-09	1,24329E-07	14183
Nat9	7,69	0,90	4,55	0,21	-1,75	7,12485E-08	1,08077E-06	66176
Fsbp	1,80	0,20	1,05	0,10	-1,75	3,42985E-07	4,7422E-06	100503583
Coa4	4,17	0,35	2,50	0,40	-1,75	3,19652E-06	3,87828E-05	68185
Lonp1	152,46	9,15	89,91	5,69	-1,75	0	0	74142
Edem1	25,58	2,48	15,05	0,72	-1,75	0	0	192193
Rwdd3	1,52	0,23	0,89	0,23	-1,75	0,001984581	0,014965291	66568
Rrm2b	8,78	0,98	5,15	0,58	-1,75	0	0	382985
Pdcd2l	9,89	0,73	5,80	0,42	-1,75	4,65816E-12	1,12496E-10	68079
Rab28	17,82	1,15	10,43	0,52	-1,75	0	0	100972
2810013P06Rik	4,45	0,46	2,58	0,49	-1,75	1,42983E-07	2,09129E-06	100503178
Osgin2	5,08	0,26	2,96	0,30	-1,75	3,67798E-10	7,31838E-09	209212
Ppef1	1,09	0,19	0,63	0,11	-1,76	0,000210431	0,001919929	237178
Tnip2	10,10	0,51	5,91	0,36	-1,76	2,77556E-15	9,0991E-14	231130
Pex16	7,68	0,95	4,54	0,05	-1,76	5,1585E-09	8,97979E-08	18633
Rfesd	4,41	0,28	2,61	0,19	-1,76	6,38867E-12	1,51884E-10	218341
Nol8	15,23	0,19	8,93	1,02	-1,76	0	0	70930
Tbc1d15	67,18	1,45	39,44	1,03	-1,76	0	0	66687
Trmt10c	19,64	0,36	11,58	0,70	-1,76	0	0	52575

Kat7	32,66	0,58	19,19	0,24	-1,76	0	0	217127
Nufip1	10,60	0,37	6,20	0,25	-1,76	0	0	27275
Rps10-ps2	2,38	1,40	1,42	1,15	-1,76	0,119044582	0,50417132	666274
Ngdn	40,13	0,66	23,49	0,59	-1,76	0	0	68966
Rbbp8	22,72	0,55	13,28	0,85	-1,76	0	0	225182
Csrnp2	5,83	0,40	3,42	0,36	-1,76	0	0	207785
Pprc1	21,97	1,54	12,90	0,73	-1,76	0	0	226169
Cirbp	25,39	0,85	14,97	0,97	-1,76	0	0	12696
Inpp5b	13,11	0,35	7,65	0,33	-1,76	0	0	16330
Krtcap3	1,27	0,67	0,75	0,29	-1,76	0,077320579	0,362039728	69815
Ttc37	10,78	0,30	6,28	0,34	-1,76	0	0	218343
Epb41l4aos	14,68	0,41	8,55	0,71	-1,76	8,9484E-14	2,574E-12	69749
Arhgef28	2,15	0,18	1,26	0,15	-1,76	5,62113E-11	1,22268E-09	110596
LOC105244102	1,22	0,31	0,70	0,18	-1,77	0,000106607	0,001025616	105244102
Tanc2	6,10	0,24	3,57	0,37	-1,77	0	0	77097
Mus81	12,31	0,90	7,19	0,26	-1,77	0	0	71711
Atp5g2	16,36	10,49	9,54	0,76	-1,77	0,002397021	0,017799718	67942
Myo6	14,49	0,50	8,44	0,29	-1,77	0	0	17920
Fbxo7	13,75	0,76	7,99	0,61	-1,77	0	0	69754
Ubr2	24,58	0,35	14,38	0,87	-1,77	0	0	224826
Tmem175	6,73	0,51	3,91	0,31	-1,77	0	0	72392
Rbm18	28,87	1,01	16,76	1,17	-1,77	0	0	67889
Clcn3	9,99	0,66	5,82	0,10	-1,77	0	0	12725
Tk2	12,07	0,76	7,01	0,61	-1,77	0	0	57813
Golga5	20,88	1,67	12,13	0,61	-1,77	0	0	27277
Tmem68	8,57	1,34	4,98	0,36	-1,77	2,09229E-11	4,75427E-10	72098
Nars2	2,83	0,21	1,64	0,07	-1,77	1,90152E-08	3,11019E-07	244141
Cep85l	1,19	0,26	0,69	0,10	-1,77	4,73771E-07	6,42433E-06	100038725
Slc4a7	8,38	0,24	4,88	0,08	-1,77	0	0	218756
Qsox2	8,60	0,36	4,99	0,51	-1,77	0	0	227638
Dusp12	20,48	0,98	11,87	0,30	-1,77	0	0	80915
A030001D20Rik	1,79	0,16	1,05	0,19	-1,77	4,39875E-06	5,23424E-05	100126243
Serp1	120,61	12,16	70,14	3,63	-1,77	0	0	28146
Wdr75	30,84	0,50	17,84	1,10	-1,78	0	0	73674
Nme6	10,53	0,39	6,09	0,57	-1,78	1,70974E-14	5,26203E-13	54369
Dpf2	42,07	3,02	24,53	0,89	-1,78	0	0	19708
Ptpn21	19,20	1,04	11,20	0,92	-1,78	0	0	24000
Rab15	2,87	0,40	1,66	0,19	-1,78	1,10476E-10	2,3418E-09	104886
Zfand2a	49,13	0,86	28,52	0,79	-1,78	0	0	100494
Fam107b	21,30	0,75	12,39	0,64	-1,78	0	0	66540
Bhlhe41	7,66	0,59	4,43	0,50	-1,78	0	0	79362
Mmp28	5,55	0,95	3,23	0,39	-1,78	8,26278E-11	1,77617E-09	118453
Acot8	2,66	0,48	1,55	0,29	-1,78	5,95739E-06	6,96754E-05	170789
Urb1	5,11	0,41	2,97	0,43	-1,78	0	0	207932
March3	3,10	0,17	1,79	0,10	-1,78	9,25371E-13	2,39669E-11	320253
Pnkp	5,72	0,02	3,35	0,59	-1,78	3,02364E-10	6,0745E-09	59047

Exog	5,81	0,39	3,35	0,17	-1,78	0	0	208194
Pvr	13,37	0,97	7,76	0,26	-1,78	0	0	52118
Arhgap33	3,01	0,32	1,75	0,20	-1,78	2,51971E-10	5,12282E-09	233071
Lrrc46	1,80	0,18	1,01	0,24	-1,78	0,002208305	0,016500091	69297
Suv420h1	9,01	0,56	5,19	0,22	-1,78	0	0	225888
Bag5	14,24	0,24	8,22	0,37	-1,78	0	0	70369
Ulk3	5,12	0,29	2,96	0,27	-1,78	2,21601E-13	6,09102E-12	71742
Noct	6,33	0,60	3,67	0,07	-1,78	5,93311E-11	1,2887E-09	12457
Ankrd1	78,16	5,99	45,24	0,89	-1,79	0	0	107765
Arrdc4	3,42	0,52	1,98	0,15	-1,79	1,5043E-09	2,78384E-08	66412
Trim23	10,13	0,46	5,84	0,20	-1,79	0	0	81003
Foxo1	4,53	0,20	2,59	0,23	-1,79	0	0	56458
Tmem161b	3,27	0,21	1,87	0,39	-1,79	5,98923E-11	1,29965E-09	72745
4921524J17Rik	16,04	1,15	9,31	0,60	-1,79	0	0	66714
Ccdc130	6,97	0,35	4,02	0,08	-1,79	1,11022E-16	3,99131E-15	67736
Paxbp1	19,75	2,22	11,35	0,59	-1,80	0	0	67367
Bbs1	1,85	0,04	1,06	0,09	-1,80	2,54964E-11	5,73105E-10	52028
Tefm	6,94	0,29	4,00	0,29	-1,80	3,99347E-13	1,07317E-11	68550
Fyn	29,64	0,94	17,07	0,56	-1,80	0	0	14360
Hspa9	611,99	0,40	351,66	7,61	-1,80	0	0	15526
Maff	18,06	1,04	10,43	0,96	-1,80	0	0	17133
1700021F05Rik	7,18	0,40	4,14	0,20	-1,80	0	0	67851
Sel1l	39,51	2,06	22,62	1,42	-1,80	0	0	20338
Pfkfb3	1,10	0,02	0,63	0,09	-1,80	4,12441E-07	5,63612E-06	170768
Krt19	4,87	1,00	2,83	0,55	-1,80	1,81393E-05	0,000197231	16669
Dph1	8,71	0,71	5,01	0,53	-1,81	4,96709E-11	1,08661E-09	116905
8030462N17Rik	8,72	0,34	4,95	0,31	-1,81	0	0	212163
Npc1	32,88	0,56	18,76	0,01	-1,81	0	0	18145
Shpk	1,45	0,12	0,83	0,15	-1,81	2,82693E-06	3,45366E-05	74637
Mical1	20,90	2,88	11,94	1,06	-1,81	0	0	171580
Ptgs2	44,16	1,60	25,20	0,88	-1,81	0	0	19225
Nfe2l1	146,76	5,76	83,62	5,25	-1,81	0	0	18023
3000002C10Rik	1,19	0,07	0,70	0,21	-1,81	0,009779232	0,062734306	378954
Daam1	5,29	0,21	2,98	0,26	-1,81	0	0	208846
Hax1	100,12	1,91	56,96	3,63	-1,81	0	0	23897
Scyl3	6,96	0,47	3,96	0,43	-1,81	0	0	240880
Mettl25	5,59	0,27	3,19	0,07	-1,81	0	0	216292
Rslcan18	1,20	0,16	0,66	0,11	-1,81	0,000115214	0,001101249	432770
Pcsk4	2,17	0,35	1,26	0,28	-1,82	7,69697E-06	8,83722E-05	18551
Optn	22,26	0,57	12,63	0,59	-1,82	0	0	71648
Wisp1	15,55	0,22	8,82	0,27	-1,82	0	0	22402
Arhgap5	13,31	0,87	7,55	0,31	-1,82	0	0	11855
Stam	20,23	0,52	11,46	0,28	-1,82	0	0	20844
Jmy	9,46	0,97	5,36	0,18	-1,82	0	0	57748
Flt3l	3,48	0,64	1,96	0,12	-1,82	3,15491E-06	3,83186E-05	14256
Atp8b3	2,69	0,24	1,55	0,31	-1,82	1,67782E-09	3,08623E-08	67331

Usp11	5,80	0,39	3,30	0,08	-1,82	0	0	236733
2610020H08Rik	1,05	0,05	0,61	0,09	-1,82	3,84938E-07	5,27446E-06	434234
Tmem67	3,77	0,21	2,13	0,06	-1,82	8,69305E-14	2,50527E-12	329795
Zfp647	3,77	0,13	2,11	0,18	-1,82	4,26996E-09	7,50729E-08	239546
Samd4	10,75	0,28	6,10	0,37	-1,82	0	0	74480
Rnmtl1	7,37	0,57	4,11	0,35	-1,83	3,31507E-09	5,90098E-08	67390
Atg3	49,94	2,13	28,19	1,05	-1,83	0	0	67841
Rbm4	8,07	0,50	4,56	0,42	-1,83	4,39904E-12	1,0652E-10	19653
Kiz	11,13	0,70	6,28	0,33	-1,83	0	0	228730
Ptar1	3,84	0,44	2,17	0,11	-1,83	0	0	72351
Sirt3	3,35	0,18	1,90	0,15	-1,83	5,71854E-07	7,66334E-06	64384
Xpnpep3	8,48	0,25	4,77	0,28	-1,83	0	0	321003
Pim3	24,19	1,11	13,72	0,52	-1,83	0	0	223775
Slc19a1	28,66	1,04	16,25	1,41	-1,83	0	0	20509
Rassf8	20,52	1,08	11,60	0,51	-1,83	0	0	71323
Lrrc8d	10,97	0,47	6,22	0,46	-1,83	0	0	231549
Ufl1	6,13	0,78	3,44	0,32	-1,83	0	0	67490
Pde4dip	10,38	0,16	5,86	0,25	-1,83	0	0	83679
4930503L19Rik	27,85	0,66	15,72	1,01	-1,83	0	0	269033
Leprotl1	45,88	1,00	25,81	1,05	-1,83	0	0	68192
Tbpl1	22,98	0,18	12,90	0,82	-1,83	0	0	237336
Ncoa1	6,08	0,45	3,43	0,34	-1,83	0	0	17977
Rcl1	21,47	0,26	12,07	0,42	-1,83	0	0	59028
Snrnp48	10,70	0,29	5,98	0,61	-1,83	0	0	67797
Plekhn1	7,45	1,27	4,23	0,57	-1,83	4,62325E-11	1,01236E-09	231002
Banp	9,38	0,53	5,29	0,18	-1,84	0	0	53325
Tnc	21,08	1,67	11,86	1,80	-1,84	0	0	21923
Mrps18b	53,76	1,59	30,05	1,61	-1,84	0	0	66973
Gm20075	27,16	2,12	15,34	1,80	-1,84	0	0	100504125
Slc30a4	71,46	2,70	40,03	2,24	-1,84	0	0	22785
Top1mt	11,67	0,24	6,53	0,42	-1,84	0	0	72960
Prodh	1,25	0,07	0,69	0,05	-1,84	0,00017146	0,00159069	19125
Gm14292	17,61	1,00	9,80	2,04	-1,84	1,90625E-13	5,29367E-12	100043168
Phlda1	29,28	3,46	16,45	0,97	-1,84	0	0	21664
Foxj3	13,71	0,15	7,67	0,25	-1,84	0	0	230700
Yrdc	17,93	0,82	10,07	0,39	-1,84	0	0	230734
Mtcl1	3,16	0,15	1,80	0,24	-1,84	0	0	68617
Pmaip1	6,61	0,31	3,68	0,26	-1,85	2,22045E-16	7,82284E-15	58801
Nfkb2	56,58	4,35	31,66	1,92	-1,85	0	0	18034
Arrdc3	13,24	0,42	7,38	0,25	-1,85	0	0	105171
Zbtb11os1	2,93	0,24	1,63	0,10	-1,85	1,70327E-05	0,000186013	66391
Garem	5,31	0,30	2,97	0,20	-1,85	0	0	381126
Mt2	477,55	10,63	266,97	5,49	-1,85	0	0	17750
Dnal4	18,46	1,69	10,34	0,12	-1,85	0	0	54152
Ppie	27,26	1,34	15,37	1,32	-1,85	0	0	56031
Zufsp	9,34	0,43	5,20	0,48	-1,85	0	0	72580

Cept1	6,03	0,29	3,34	0,21	-1,85	0	0	99712
3110043O21Rik	5,74	0,42	3,21	0,09	-1,85	0	0	73205
Rrs1	28,57	1,50	15,95	1,21	-1,85	0	0	59014
Rnf113a1	4,47	0,93	2,54	0,27	-1,85	6,62275E-06	7,68874E-05	69942
Gm6654	1,09	0,39	0,55	0,38	-1,85	0,080581957	0,37405929	626175
Slc45a3	2,31	0,14	1,28	0,29	-1,85	2,56244E-08	4,12922E-07	212980
Cpeb4	8,67	0,70	4,84	0,14	-1,85	0	0	67579
Sco1	7,08	0,36	3,92	0,42	-1,85	0	0	52892
Sft2d2	23,73	0,28	13,19	0,19	-1,86	0	0	108735
Ppip5k2	18,07	0,90	10,02	0,30	-1,86	0	0	227399
Pgf	4,92	0,13	2,72	0,50	-1,86	1,65153E-09	3,0391E-08	18654
Wdr17	1,07	0,07	0,60	0,03	-1,86	2,60738E-07	3,67047E-06	244484
Tmod1	1,76	0,15	0,96	0,32	-1,86	1,18634E-05	0,000132163	21916
Wdr31	1,61	0,31	0,89	0,11	-1,86	3,8385E-06	4,6047E-05	71354
Armcx5	6,75	0,69	3,70	0,30	-1,86	4,7905E-12	1,15571E-10	494468
Zfp746	15,64	0,65	8,69	0,27	-1,86	0	0	69228
AW549877	34,19	3,67	18,93	1,89	-1,86	0	0	106064
Ccdc134	12,67	0,47	7,05	0,64	-1,86	0	0	76457
Idua	4,90	0,11	2,71	0,17	-1,86	0	0	15932
Arl15	7,05	0,59	3,88	0,38	-1,86	0	0	218639
Zbtb18	3,93	0,26	2,15	0,22	-1,86	0	0	30928
Naf1	16,28	1,46	8,94	0,74	-1,87	0	0	234344
LOC102634078	1,53	0,15	0,86	0,20	-1,87	0,000179422	0,001659176	102634078
Mkrn2	20,18	0,15	11,16	0,62	-1,87	0	0	67027
Usp53	6,73	0,22	3,70	0,10	-1,87	0	0	99526
Taf1d	42,79	0,72	23,59	0,65	-1,87	0	0	75316
LOC108168395	8,16	0,68	4,46	0,95	-1,87	1,21347E-13	3,44294E-12	108168395
Vimp	70,07	6,86	38,48	1,62	-1,87	0	0	109815
Plk2	121,76	3,83	67,09	0,94	-1,87	0	0	20620
Tmem206	6,61	0,44	3,65	0,12	-1,88	0	0	66950
Gm5801	1,02	0,76	0,56	0,49	-1,88	0,163971412	0,634064357	545056
Spdya	2,42	0,40	1,33	0,07	-1,88	1,42476E-08	2,36333E-07	70891
Hvcn1	9,52	0,67	5,25	0,13	-1,88	0	0	74096
5930430L01Rik	3,18	0,24	1,77	0,11	-1,88	2,22045E-16	7,82284E-15	319982
Gm14117	1,82	0,42	0,96	0,73	-1,88	0,029339726	0,162440427	100418259
Zfp622	27,50	0,42	15,05	0,92	-1,88	0	0	52521
Slc23a2	15,44	0,41	8,49	0,24	-1,88	0	0	54338
Gareml	3,64	0,36	2,02	0,30	-1,88	5,33718E-11	1,16479E-09	242915
Sfxn4	1,44	0,03	0,79	0,13	-1,88	1,5692E-06	1,9789E-05	94281
Sars	161,75	6,13	88,78	4,25	-1,88	0	0	20226
Igf2r	33,04	1,14	18,12	1,86	-1,88	0	0	16004
Frs3	2,47	0,47	1,35	0,03	-1,88	9,9043E-07	1,28369E-05	107971
Ngrn	10,72	0,49	5,87	0,25	-1,88	3,21965E-15	1,04576E-13	83485
1700037C18Rik	3,69	0,27	2,07	0,38	-1,88	1,68371E-06	2,11399E-05	73261
Egfr	3,32	0,30	1,84	0,18	-1,88	5,30354E-13	1,41278E-11	13649
Tor3a	13,99	0,48	7,71	0,61	-1,88	0	0	30935

Elmo3	2,32	0,12	1,27	0,23	-1,89	4,28925E-08	6,70415E-07	234683
E130311K13Rik	4,93	0,53	2,66	0,37	-1,89	1,6396E-09	3,02078E-08	329659
Gfra1	10,54	0,23	5,73	0,35	-1,89	0	0	14585
2210418O10Rik	5,49	3,55	3,01	2,81	-1,89	0,187728969	0,701282854	100504263
Ddx50	62,50	3,97	34,02	0,70	-1,89	0	0	94213
Zscan22	4,24	0,05	2,29	0,19	-1,89	6,66134E-16	2,2956E-14	232878
Sash1	2,10	0,29	1,13	0,19	-1,90	2,54241E-13	6,94647E-12	70097
Nfkbie	23,10	3,07	12,54	0,75	-1,90	0	0	18037
Dhrs11	8,61	0,43	4,69	0,45	-1,90	1,05967E-10	2,25247E-09	192970
AW209491	13,06	0,68	7,06	0,54	-1,90	0	0	105351
Nop14	62,18	3,08	33,62	1,37	-1,91	0	0	75416
Eif5	137,04	1,01	74,14	4,06	-1,91	0	0	217869
Dld	89,03	2,30	48,11	1,36	-1,91	0	0	13382
Phf10	41,55	1,07	22,36	1,26	-1,91	0	0	72057
Noc3l	23,47	1,22	12,64	0,32	-1,91	0	0	57753
Gdap1l1	1,40	0,19	0,74	0,24	-1,91	0,000103288	0,000995994	228858
Det1	4,18	0,52	2,28	0,38	-1,91	2,30699E-11	5,20865E-10	76375
Gdnf	1,44	0,20	0,79	0,13	-1,91	1,61477E-07	2,34752E-06	14573
Tmem38b	3,73	0,03	1,98	0,29	-1,91	3,24008E-10	6,48087E-09	52076
Zfp184	4,47	0,60	2,39	0,23	-1,91	1,68584E-11	3,85955E-10	193452
Tbce	12,21	0,47	6,60	0,30	-1,92	0	0	70430
Twistnb	26,17	0,85	14,01	0,81	-1,92	0	0	28071
LOC108168974	3,60	0,41	1,94	0,45	-1,92	2,76998E-08	4,44177E-07	108168974
Cebpd	26,18	1,45	14,10	0,52	-1,92	0	0	12609
Fam60a	14,21	1,37	7,63	0,69	-1,92	0	0	56306
Foxn2	6,47	0,13	3,49	0,09	-1,92	0	0	14236
Zbtb11	9,66	0,18	5,18	0,07	-1,92	0	0	271377
Zfp810	1,31	0,21	0,71	0,07	-1,92	3,38151E-06	4,09079E-05	235050
Gm4462	1,17	0,18	0,62	0,15	-1,92	0,003896504	0,027548816	100043475
Pdia4	213,65	15,58	114,64	12,69	-1,92	0	0	12304
Nle1	11,27	0,94	6,10	0,28	-1,92	0	0	217011
Gtpbp4	35,39	0,17	18,95	0,79	-1,93	0	0	69237
Acsbg1	1,68	0,18	0,90	0,11	-1,93	1,95687E-07	2,81181E-06	94180
Ptpn2	11,34	0,47	6,07	0,38	-1,93	0	0	19255
Thumpd2	1,39	0,13	0,73	0,12	-1,93	2,33633E-10	4,77118E-09	72167
Cmtr2	7,67	0,35	4,08	0,29	-1,93	0	0	234728
Grem2	6,30	0,59	3,39	0,06	-1,93	0	0	23893
Deb1	22,59	2,22	11,87	0,96	-1,93	7,40176E-11	1,59708E-09	26901
Zfp507	11,66	0,18	6,22	0,21	-1,94	0	0	668501
Trim35	63,02	3,04	33,62	1,20	-1,94	0	0	66854
Vnn1	1,49	0,16	0,81	0,17	-1,94	2,72298E-05	0,000287933	22361
Asns	160,11	1,96	85,17	2,62	-1,94	0	0	27053
Lama2	19,23	0,67	10,23	1,45	-1,94	0	0	16773
Zfp39	2,60	0,17	1,38	0,08	-1,94	2,89149E-10	5,82178E-09	22698
Rnf114	21,64	1,72	11,52	0,85	-1,94	0	0	81018
Mars	56,52	2,62	30,05	2,32	-1,94	0	0	216443

Lgals8	36,08	0,34	19,11	1,10	-1,94	0	0	56048
Eif4ebp1	277,08	18,61	147,19	10,92	-1,94	0	0	13685
Cngb1	2,10	0,19	1,14	0,17	-1,95	2,06368E-12	5,16654E-11	333329
Fam161b	1,04	0,10	0,55	0,04	-1,95	9,63194E-06	0,000108922	217705
Enpp2	1,72	0,37	0,91	0,07	-1,95	1,88493E-08	3,08637E-07	18606
Nhlrc3	9,15	0,45	4,89	0,62	-1,95	1,11022E-16	3,99131E-15	212114
Efcab7	6,55	0,84	3,46	0,31	-1,95	2,56871E-11	5,76543E-10	230500
Cobl	1,25	0,10	0,66	0,08	-1,95	3,81845E-09	6,74456E-08	12808
Surf2	29,84	1,51	15,79	1,31	-1,95	0	0	20931
Fam129a	50,51	2,03	26,75	0,45	-1,95	0	0	63913
Xylb	3,45	0,36	1,80	0,20	-1,95	8,24896E-14	2,38329E-12	102448
Fam199x	3,49	0,58	1,84	0,14	-1,95	1,33227E-15	4,48648E-14	245622
Elmsan1	9,02	0,57	4,76	0,40	-1,95	0	0	238317
Nsun3	5,88	0,46	3,11	0,14	-1,95	0	0	106338
Ticam1	11,56	0,29	6,06	0,42	-1,96	0	0	106759
Lmbrd1	15,22	1,00	8,03	0,21	-1,96	0	0	68421
Oxnad1	4,37	0,19	2,28	0,24	-1,96	0	0	218885
Gtpbp10	7,94	0,76	4,17	0,25	-1,96	0	0	207704
Pparg	2,22	0,14	1,18	0,12	-1,96	3,62233E-09	6,41548E-08	19016
Orc4	14,03	0,28	7,37	0,46	-1,96	0	0	26428
LOC108167950	1,29	0,18	0,65	0,41	-1,96	0,012902817	0,080016805	108167950
Lrig3	15,40	0,54	8,14	0,28	-1,96	0	0	320398
Rbpj	77,46	2,69	40,79	1,64	-1,96	0	0	19664
Sh2d4a	2,00	0,14	1,06	0,04	-1,96	9,52947E-08	1,4215E-06	72281
Ngf	11,91	1,23	6,29	0,06	-1,96	0	0	18049
Nfx1	26,57	0,88	13,95	0,32	-1,97	0	0	74164
Trim56	10,90	0,26	5,77	0,94	-1,97	0	0	384309
Setd6	18,78	1,61	9,76	0,53	-1,97	0	0	66083
Hemk1	5,79	0,85	3,03	0,36	-1,97	1,79856E-13	5,00372E-12	69536
Slc25a29	1,45	0,08	0,75	0,15	-1,97	0,000111389	0,001066464	214663
Trmt61a	8,12	1,03	4,30	0,52	-1,97	3,9857E-14	1,18599E-12	328162
Abcb1a	4,01	0,28	2,09	0,27	-1,97	1,24345E-14	3,87119E-13	18671
Ccdc47	80,47	0,87	42,06	1,06	-1,97	0	0	67163
LOC102636028	2,36	0,36	1,20	0,12	-1,97	8,78689E-05	0,00085834	102636028
Atp2a3	6,05	0,32	3,13	0,43	-1,98	0	0	53313
Cers6	5,41	0,23	2,82	0,20	-1,98	0	0	241447
Nop58	141,50	1,07	73,75	1,44	-1,98	0	0	55989
Rin1	30,68	1,92	15,98	2,05	-1,98	0	0	225870
Rpl10a	115,57	1,88	60,28	9,01	-1,99	0	0	19896
Ccdc112	9,59	0,21	4,94	0,26	-1,99	0	0	240261
Smim3	7,42	0,62	3,89	0,40	-1,99	0	0	106878
Mib2	6,72	0,68	3,51	0,22	-1,99	0	0	76580
Cttnbp2nl	20,83	1,03	10,74	0,77	-2,00	0	0	80281
Sp140	2,95	0,21	1,54	0,50	-2,00	6,14095E-07	8,19122E-06	434484
Harbi1	3,00	0,39	1,53	0,30	-2,00	2,08503E-11	4,74026E-10	241547
Bdkrb2	5,37	0,72	2,79	0,15	-2,00	1,22125E-15	4,12779E-14	12062

Mex3b	4,66	0,11	2,43	0,31	-2,00	0	0	108797
Arfrp1	16,10	0,97	8,28	0,51	-2,00	0	0	76688
Gm8445	7,76	0,33	3,94	0,55	-2,00	1,90262E-10	3,92223E-09	667077
Timm8a1	83,10	3,25	42,74	0,80	-2,00	0	0	30058
Gm4924	7,19	0,66	3,66	0,31	-2,01	0	0	237412
Hgh1	13,99	0,31	7,22	0,91	-2,01	0	0	59053
Slc17a9	1,06	0,21	0,55	0,03	-2,01	0,000113376	0,001084807	228993
Gnl3	77,37	0,29	39,62	2,13	-2,01	0	0	30877
Gm10130	6,37	0,20	3,25	0,11	-2,01	0	0	102633750
2610005L07Rik	2,68	0,19	1,37	0,05	-2,02	0	0	381598
Atp2a2	101,27	0,96	51,82	2,26	-2,02	0	0	11938
D8Ertd82e	1,91	0,41	0,99	0,24	-2,02	4,31884E-08	6,74579E-07	244418
Gars	275,54	9,37	141,00	5,53	-2,02	0	0	353172
Rab20	4,96	1,10	2,55	0,10	-2,02	1,67894E-07	2,43462E-06	19332
Cep126	1,15	0,22	0,58	0,02	-2,02	2,10606E-08	3,43125E-07	234915
Nmd3	50,63	1,72	25,76	0,67	-2,02	0	0	97112
Prpsap2	18,04	0,23	9,09	0,92	-2,03	0	0	212627
Etv5	8,55	0,41	4,35	0,16	-2,03	0	0	104156
Calcrl	4,86	0,15	2,46	0,14	-2,03	0	0	54598
Pogk	3,09	0,12	1,55	0,13	-2,03	0	0	71592
Ube2w	6,28	0,22	3,16	0,34	-2,03	0	0	66799
Zfp951	3,18	0,15	1,63	0,43	-2,03	2,62013E-14	7,92532E-13	626391
Zfpm2	32,35	0,76	16,44	1,12	-2,03	0	0	22762
Wars	90,95	3,68	46,19	0,90	-2,03	0	0	22375
Ibtk	16,15	0,51	8,16	0,35	-2,03	0	0	108837
Fam171b	19,79	0,80	9,99	0,74	-2,03	0	0	241520
Cep290	6,53	0,64	3,31	0,12	-2,04	0	0	216274
Zfp598	39,55	3,83	20,08	1,27	-2,04	0	0	213753
Zfp330	47,77	1,36	24,16	1,16	-2,04	0	0	30932
Dgat2	8,29	0,31	4,16	0,28	-2,04	0	0	67800
Cep162	7,71	0,24	3,90	0,13	-2,04	0	0	382090
Dph7	6,67	0,25	3,35	0,22	-2,04	0	0	67228
Zfp777	9,70	0,80	4,90	0,23	-2,04	0	0	72306
Bhlhe40	22,31	1,24	11,31	0,52	-2,05	0	0	20893
Tnfaip2	65,94	6,59	33,29	2,82	-2,05	0	0	21928
Sepsecs	4,01	0,38	2,01	0,09	-2,05	6,14042E-12	1,46058E-10	211006
1700096K18Rik	1,78	0,15	0,88	0,16	-2,05	0,000114035	0,001090663	73571
Itprip	23,25	2,91	11,70	1,02	-2,05	0	0	414801
Tgif1	26,81	1,33	13,50	0,23	-2,05	0	0	21815
Hyou1	111,44	4,15	55,94	2,13	-2,05	0	0	12282
Tmem11	26,44	3,15	13,33	0,38	-2,06	0	0	216821
AU041133	3,69	0,39	1,81	0,25	-2,06	2,09327E-11	4,75427E-10	216177
Mfsd7c	1,30	0,08	0,65	0,10	-2,06	1,93453E-07	2,7867E-06	217721
Polr3e	7,99	0,52	4,01	0,30	-2,06	0	0	26939
Nfkbil1	18,27	0,84	9,22	0,56	-2,06	0	0	18038
Ier3	82,65	8,64	41,46	2,42	-2,06	0	0	15937

Trim34b	1,52	0,13	0,75	0,17	-2,06	0,000195295	0,00179616	434218
D10Wsu102e	49,83	0,41	24,88	0,67	-2,07	0	0	28109
D330041H03Rik	2,79	0,33	1,37	0,21	-2,07	9,61453E-14	2,75868E-12	654822
Dhrs3	2,38	0,27	1,19	0,15	-2,07	2,1105E-10	4,33319E-09	20148
Prrc1	15,63	0,49	7,82	0,34	-2,07	0	0	73137
Snora64	3,80	1,16	1,72	1,37	-2,07	0,093622731	0,41969285	104366
LOC108168832	3,69	1,19	1,77	0,88	-2,07	0,005235052	0,035913982	108168832
Rchy1	40,35	2,24	19,98	0,85	-2,07	0	0	68098
Crls1	26,98	1,31	13,38	0,09	-2,08	0	0	66586
Hivep2	2,36	0,15	1,17	0,05	-2,08	0	0	15273
Ift88	3,96	0,22	1,95	0,21	-2,08	0	0	21821
Uprt	14,87	1,50	7,36	1,06	-2,08	6,55032E-15	2,08612E-13	331487
Cdkn2aip	14,26	0,63	7,04	0,52	-2,08	0	0	70925
Snai2	23,69	1,36	11,68	0,65	-2,08	0	0	20583
Ccdc181	9,18	0,15	4,49	0,55	-2,08	0	0	74895
Eif2s2	193,63	2,64	95,80	2,59	-2,08	0	0	67204
Zfp3	3,49	0,46	1,75	0,41	-2,08	2,49785E-08	4,0408E-07	193043
Abtb2	5,51	0,45	2,75	0,14	-2,08	0	0	99382
Nars	347,01	7,23	171,66	5,11	-2,09	0	0	70223
Ilf3	45,89	1,30	22,74	0,87	-2,09	0	0	16201
Aaed1	57,33	3,45	28,21	1,06	-2,09	0	0	66129
Sirt1	30,44	1,86	15,00	1,11	-2,09	0	0	93759
Tmem86a	9,19	0,60	4,53	0,15	-2,09	0	0	67893
Syvn1	31,35	0,95	15,49	0,37	-2,09	0	0	74126
Ccdc9	13,28	0,71	6,60	0,60	-2,09	0	0	243846
Triqk	3,73	0,28	1,84	0,30	-2,09	1,00015E-08	1,68526E-07	208820
Pla2g2e	1,57	0,42	0,80	0,26	-2,09	0,002592914	0,019124048	26970
Gm4070	5,12	0,44	2,54	0,25	-2,09	0	0	100042856
Gm8783	4,53	0,40	2,21	0,82	-2,10	2,16069E-09	3,93647E-08	667723
Mtbp	15,19	0,66	7,48	0,53	-2,10	0	0	105837
Pdzd8	32,08	1,37	15,71	0,30	-2,10	0	0	107368
Nrg1	4,55	0,31	2,24	0,24	-2,11	0	0	211323
Mmp19	7,91	0,28	3,86	0,25	-2,11	0	0	58223
Nr2c1	9,81	0,21	4,79	0,09	-2,11	0	0	22025
Aars	221,34	10,68	108,30	6,85	-2,11	0	0	234734
Ccdc186	20,76	0,29	10,14	0,28	-2,11	0	0	213993
Zfp1	11,81	0,02	5,75	0,32	-2,11	0	0	22640
Plp1	1,85	0,11	0,90	0,08	-2,11	4,17696E-10	8,26452E-09	18823
Fbxl2	6,14	0,46	3,01	0,04	-2,12	0	0	72179
Plk3	12,28	1,11	6,02	0,26	-2,12	0	0	12795
Gpcpd1	7,24	0,11	3,51	0,07	-2,12	0	0	74182
Slc11a2	25,72	1,61	12,56	0,67	-2,12	0	0	18174
Gata3	3,78	0,66	1,84	0,13	-2,12	3,23075E-14	9,70816E-13	14462
Fsd1l	2,59	0,16	1,25	0,13	-2,12	0	0	319636
Pex11g	2,22	0,31	1,06	0,17	-2,12	4,42458E-09	7,76422E-08	69129
Wdfy1	4,71	0,21	2,30	0,23	-2,12	0	0	69368

Tiparp	20,38	1,23	9,88	0,27	-2,13	0	0	99929
Mtpap	14,61	0,71	7,03	0,48	-2,13	0	0	67440
Zfp930	11,39	0,44	5,51	0,54	-2,14	0	0	234358
Eif2b3	12,35	0,45	5,94	0,39	-2,14	0	0	108067
Lamb1	151,72	6,64	73,29	7,05	-2,14	0	0	16777
Rasgef1a	1,28	0,09	0,61	0,08	-2,14	2,22591E-10	4,55584E-09	70727
Cdsn	32,95	1,13	15,88	0,69	-2,14	0	0	386463
Cry2	5,85	0,45	2,83	0,07	-2,14	0	0	12953
Fgf2	1,08	0,16	0,50	0,23	-2,14	0,012217333	0,076216773	14173
Mtss1l	10,70	1,06	5,18	0,48	-2,14	0	0	244654
Wdr43	63,29	0,66	30,54	0,37	-2,14	0	0	72515
Gtpbp2	21,13	1,41	10,24	0,83	-2,14	0	0	56055
Ddx31	8,42	0,59	4,05	0,21	-2,14	0	0	227674
Piga	8,60	0,06	4,14	0,14	-2,14	0	0	18700
Syne1	6,03	0,60	2,90	0,39	-2,15	0	0	64009
Zfp623	13,20	0,64	6,34	0,24	-2,15	0	0	78834
Accs	2,92	0,34	1,41	0,05	-2,15	0	0	329470
Fbxo42	14,57	0,61	6,99	0,45	-2,15	0	0	213499
Zyg11b	18,55	0,30	8,88	0,28	-2,15	0	0	414872
Nfu1	70,78	1,88	33,81	1,94	-2,16	0	0	56748
Cwf19l2	7,69	0,32	3,71	0,36	-2,16	0	0	244672
Zfp94	1,98	0,26	0,94	0,15	-2,16	6,77858E-12	1,60653E-10	22756
LOC102635048	1,33	0,34	0,60	0,55	-2,16	0,028832874	0,160047479	102635048
Rassf5	7,52	0,19	3,62	0,31	-2,16	0	0	54354
Zcchc7	15,38	0,13	7,31	0,42	-2,17	0	0	319885
Alg2	19,12	0,47	9,11	0,33	-2,17	0	0	56737
Cxcl1	137,20	12,00	65,26	7,43	-2,17	0	0	14825
Manea	12,65	0,74	6,03	0,36	-2,17	0	0	242362
4632404H12Rik	1,77	0,08	0,85	0,10	-2,17	3,09863E-13	8,41596E-12	74034
Eprs	122,05	1,63	58,03	2,21	-2,17	0	0	107508
Zzz3	23,38	0,59	11,11	0,03	-2,17	0	0	108946
A830010M20Rik	2,43	0,16	1,15	0,07	-2,18	0	0	231570
Klra5	1,46	0,43	0,72	0,23	-2,18	0,001565107	0,012070633	16636
Zfp566	5,04	0,77	2,43	0,40	-2,18	1,34167E-11	3,10894E-10	72556
Crebrf	14,46	0,92	6,83	0,23	-2,18	0	0	77128
Gtpbp6	14,59	1,26	6,93	0,60	-2,18	0	0	107999
Dus2	7,96	0,67	3,71	0,45	-2,18	0	0	66369
Uhrf1bp1	12,78	0,52	6,04	0,41	-2,19	0	0	224648
Rps6ka2	22,44	0,70	10,58	0,86	-2,19	0	0	20112
Jrk	3,71	0,28	1,76	0,08	-2,19	0	0	16469
Per2	1,33	0,10	0,64	0,14	-2,19	3,64118E-10	7,25146E-09	18627
Rnf138	11,19	1,01	5,24	0,56	-2,19	0	0	56515
Samd8	10,09	0,80	4,76	0,20	-2,19	0	0	67630
Kdm5b	3,48	0,15	1,66	0,21	-2,20	0	0	75605
Icam1	2,56	0,81	1,20	0,13	-2,20	2,68789E-07	3,77914E-06	15894
Klf4	62,13	2,35	29,22	1,44	-2,20	0	0	16600

Trdmt1	1,91	0,11	0,89	0,01	-2,20	5,8985E-12	1,40596E-10	13434
Cnnm4	8,00	0,28	3,76	0,06	-2,20	0	0	94220
Jag1	1,62	0,14	0,77	0,03	-2,20	0	0	16449
Tmem144	8,89	0,63	4,14	0,35	-2,20	0	0	70652
Chd5	2,03	0,31	0,95	0,21	-2,20	5,55112E-15	1,77779E-13	269610
Pck2	66,11	4,86	31,01	1,55	-2,20	0	0	74551
Btbd8	2,19	0,65	1,01	0,26	-2,21	0,000196183	0,001802513	100503185
Lmo4	31,34	1,32	14,61	0,66	-2,21	0	0	16911
Akr1b7	2,71	0,32	1,27	0,18	-2,21	1,66875E-06	2,09752E-05	11997
Antxr2	27,79	0,66	12,95	0,63	-2,21	0	0	71914
Ttc39b	6,69	0,06	3,13	0,14	-2,21	0	0	69863
2310034P14Rik	1,01	0,04	0,48	0,16	-2,21	0,003421344	0,024507407	69579
Atf6	17,47	0,69	8,15	0,03	-2,21	0	0	226641
Cyb5r1	146,46	4,96	67,99	1,23	-2,22	0	0	72017
Slc26a11	6,27	0,25	2,93	0,14	-2,22	0	0	268512
Plcl2	1,35	0,32	0,63	0,08	-2,23	2,23902E-08	3,63881E-07	224860
Slc38a7	42,82	2,14	19,83	0,31	-2,23	0	0	234595
Siah2	12,57	0,61	5,87	0,66	-2,23	0	0	20439
Lrrc73	1,81	0,11	0,86	0,36	-2,24	5,96122E-05	0,000596709	224813
Hook2	14,57	0,62	6,71	0,19	-2,24	0	0	170833
Klf2	29,81	0,99	13,70	1,02	-2,24	0	0	16598
Dnaja3	37,14	0,75	17,02	1,14	-2,25	0	0	83945
Epg5	9,25	0,55	4,27	0,49	-2,25	0	0	100502841
Qtrt1	11,95	0,89	5,56	0,43	-2,25	0	0	60507
Als2cl	5,50	0,45	2,55	0,33	-2,25	0	0	235633
Gfpt2	6,33	0,87	2,92	0,32	-2,26	0	0	14584
Tmem47	3,56	0,24	1,63	0,18	-2,26	0	0	192216
Xpot	81,35	0,46	37,20	1,78	-2,26	0	0	73192
Gm32856	6,83	0,92	3,11	0,26	-2,26	0	0	102635552
Foxs1	2,89	0,34	1,35	0,22	-2,26	1,7942E-07	2,5919E-06	14239
2700046G09Rik	1,14	0,31	0,51	0,10	-2,26	0,000252666	0,002273547	67188
Plag1	2,88	0,21	1,30	0,07	-2,26	0	0	56711
BC024978	3,00	0,06	1,35	0,12	-2,27	0	0	414069
Irx1	10,11	0,52	4,58	0,91	-2,27	0	0	16371
Itfg2	24,48	2,42	11,11	0,24	-2,28	0	0	101142
Tcaim	2,35	0,05	1,05	0,10	-2,28	0	0	382117
Hdac4	5,50	0,22	2,50	0,20	-2,28	0	0	208727
Pard6a	12,55	0,40	5,64	0,26	-2,28	0	0	56513
Ier2	33,18	3,02	15,05	1,23	-2,29	0	0	15936
Pnrc2	67,07	3,62	30,15	1,82	-2,29	0	0	52830
Cftr	4,81	0,32	2,17	0,28	-2,29	0	0	12638
Rpp38	4,25	0,57	1,90	0,08	-2,30	0	0	227522
Slc7a5	154,53	7,71	69,15	8,41	-2,31	0	0	20539
Stambpl1	8,81	0,10	3,94	0,34	-2,31	0	0	76630
Aff1	21,06	0,47	9,44	0,43	-2,31	0	0	17355
Zfp655	20,14	0,32	8,99	0,35	-2,31	0	0	72611

Cxcl2	2,79	0,50	1,21	0,54	-2,31	2,85667E-05	0,000300679	20310
Dtna	7,16	0,27	3,21	0,35	-2,31	0	0	13527
Bbs5	2,72	0,18	1,20	0,17	-2,32	3,69704E-14	1,10441E-12	72569
Manf	138,71	10,38	61,56	3,80	-2,32	0	0	74840
Hist1h1c	3,15	0,55	1,42	0,23	-2,32	5,60126E-05	0,000563636	50708
Arhgef2	38,10	1,32	16,94	0,92	-2,32	0	0	16800
Entpd2	1,05	0,18	0,48	0,10	-2,32	0,000131239	0,001242739	12496
Trim34a	1,52	0,15	0,66	0,17	-2,33	5,25117E-08	8,09429E-07	94094
Nrip2	3,46	0,24	1,56	0,20	-2,33	2,30926E-14	7,0315E-13	60345
Chd2	16,97	1,18	7,51	0,34	-2,33	0	0	244059
Myc	75,20	5,55	33,35	3,71	-2,33	0	0	17869
Zfp639	13,01	0,33	5,73	0,45	-2,34	0	0	67778
Gm6223	2,95	0,38	1,28	1,46	-2,34	0,023961781	0,136990747	621414
2410006H16Rik	109,04	9,43	48,02	1,30	-2,35	0	0	69221
Cxcl10	36,69	2,35	16,20	0,90	-2,35	0	0	15945
Mknk1	14,96	1,09	6,61	0,24	-2,35	0	0	17346
Spata5l1	3,90	0,32	1,71	0,12	-2,35	3,9318E-10	7,80646E-09	214616
Neurl3	3,15	0,40	1,37	0,27	-2,35	5,15032E-13	1,37277E-11	214854
Lhfpl2	13,44	0,14	5,90	0,15	-2,36	0	0	218454
Fam20a	8,44	0,60	3,72	0,29	-2,36	0	0	208659
Got1	54,11	1,49	23,74	0,50	-2,36	0	0	14718
Nnmt	3,17	0,11	1,40	0,09	-2,36	2,22045E-15	7,3318E-14	18113
Setd4	1,77	0,12	0,77	0,05	-2,36	9,3614E-13	2,42322E-11	224440
Bnip3l-ps	1,81	1,79	0,78	0,69	-2,36	0,10745385	0,466580374	100043324
Fam84b	4,36	0,34	1,92	0,13	-2,36	0	0	399603
Spata5	19,06	0,59	8,29	0,41	-2,36	0	0	57815
Steap1	41,83	1,14	18,39	1,40	-2,36	0	0	70358
F3	10,62	0,71	4,55	0,51	-2,37	0	0	14066
Pawr	5,67	0,54	2,48	0,30	-2,37	1,44329E-15	4,84253E-14	114774
Tnfrsf11b	3,66	0,71	1,59	0,06	-2,38	9,41469E-14	2,70303E-12	18383
Cebpg	18,46	0,98	7,99	0,29	-2,38	0	0	12611
Ficd	4,93	0,29	2,12	0,18	-2,38	0	0	231630
Engase	1,44	0,09	0,62	0,04	-2,38	2,55987E-11	5,74896E-10	217364
Mtus1	1,05	0,03	0,45	0,02	-2,38	5,69544E-14	1,66551E-12	102103
Alkbh1	12,24	0,44	5,29	0,09	-2,39	0	0	211064
Pdcd2	36,29	1,51	15,51	1,42	-2,39	0	0	18567
Cxadr	4,05	0,04	1,74	0,06	-2,39	0	0	13052
Ern1	21,23	0,75	9,22	0,88	-2,39	0	0	78943
Gm7334	7,65	3,07	3,34	3,55	-2,39	0,042883649	0,224140039	654432
Sqstm1	713,34	52,53	307,87	10,06	-2,39	0	0	18412
Cry1	11,60	0,25	5,01	0,28	-2,40	0	0	12952
Zfp938	12,74	1,01	5,42	0,69	-2,40	0	0	237411
Tcp11l2	10,79	1,06	4,68	0,30	-2,40	0	0	216198
Qtrtd1	13,02	0,94	5,53	0,54	-2,40	0	0	106248
Cars	41,34	2,04	17,77	1,50	-2,41	0	0	27267
Kdm6b	4,18	0,33	1,81	0,17	-2,41	0	0	216850

Mthfr-ps1	3,28	0,56	1,42	0,38	-2,42	3,55879E-08	5,61808E-07	387594
Car6	22,57	1,26	9,52	0,86	-2,42	0	0	12353
Mars2	4,22	0,50	1,82	0,18	-2,42	0	0	212679
Cbarp	7,12	0,59	3,05	0,20	-2,43	0	0	100503659
Amdhd2	98,25	7,11	41,78	0,28	-2,43	0	0	245847
Zfp939	1,38	0,08	0,58	0,04	-2,43	2,51354E-13	6,87171E-12	233147
Kyat1	2,61	0,31	1,10	0,11	-2,43	2,8344E-13	7,73041E-12	70266
Mdfic	28,87	0,14	12,17	0,81	-2,43	0	0	16543
Prmt6	7,23	0,43	3,08	0,14	-2,44	0	0	99890
Chka	10,04	0,74	4,25	0,38	-2,44	0	0	12660
Gm5345	11,52	0,40	4,86	0,68	-2,45	0	0	384808
Tigd2	11,98	0,87	5,02	0,53	-2,45	0	0	68140
Eea1	42,36	0,66	17,87	0,52	-2,45	0	0	216238
Apobec1	5,06	0,18	2,10	0,42	-2,45	0	0	11810
Ift172	11,65	0,56	4,91	0,26	-2,45	0	0	67661
Nr1d2	32,49	0,92	13,60	0,40	-2,46	0	0	353187
Cebpb	46,24	0,70	19,27	0,94	-2,46	0	0	12608
Gm21948	1,03	0,21	0,44	0,03	-2,46	1,51481E-05	0,000166367	100328588
Atp5l	2,66	1,99	1,13	1,37	-2,47	0,060616257	0,29790625	27425
Rsbn1	7,00	0,46	2,92	0,11	-2,47	0	0	229675
Gnpnat1	24,52	0,84	10,18	0,91	-2,48	0	0	54342
Rhbdd1	22,44	0,52	9,32	0,45	-2,48	0	0	76867
Srfbp1	28,84	0,61	11,90	0,65	-2,49	0	0	67222
Il17ra	15,52	1,40	6,44	0,22	-2,50	0	0	16172
Slc20a1	56,89	0,54	23,45	0,53	-2,50	0	0	20515
Zbtb21	3,51	0,08	1,45	0,08	-2,50	0	0	114565
Hey2	1,50	0,19	0,61	0,17	-2,51	5,39269E-08	8,30685E-07	15214
Iars	102,08	0,37	41,94	3,26	-2,51	0	0	105148
Spry4	2,65	0,18	1,09	0,04	-2,52	0	0	24066
6330416G13Rik	3,60	0,23	1,49	0,19	-2,52	0	0	230279
Usp36	11,71	0,70	4,82	0,27	-2,52	0	0	72344
D16Ertd472e	7,86	0,29	3,22	0,06	-2,52	0	0	67102
Trmt13	4,72	0,08	1,94	0,27	-2,53	0	0	229780
Hectd2	2,07	0,15	0,83	0,13	-2,54	0	0	226098
Gem	4,11	0,38	1,66	0,04	-2,55	2,22045E-16	7,82284E-15	14579
Gm32457	1,24	0,38	0,50	0,21	-2,55	0,000118191	0,001128285	102635015
Catsper2	1,27	0,08	0,51	0,05	-2,56	1,80488E-11	4,12383E-10	212670
Ereg	6,01	0,36	2,40	0,33	-2,56	0	0	13874
Vldlr	6,13	0,16	2,47	0,31	-2,56	0	0	22359
Trit1	9,82	0,46	3,94	0,19	-2,57	0	0	66966
Yars	76,95	2,71	31,04	2,70	-2,57	0	0	107271
Spata7	6,00	0,46	2,38	0,33	-2,57	0	0	104871
Gm6485	1,02	0,33	0,38	0,13	-2,57	0,004004259	0,02824521	624251
Klf11	10,77	0,51	4,30	0,11	-2,58	0	0	194655
Rn7s2	12,88	13,86	5,17	0,85	-2,58	0,003770534	0,0267574	103949
Zfp964	2,51	0,35	1,00	0,11	-2,59	0	0	636741

Nfxl1	18,71	0,31	7,42	0,12	-2,60	0	0	100978
Usp18	5,32	0,26	2,09	0,33	-2,60	0	0	24110
Adck3	1,59	0,07	0,62	0,10	-2,60	1,38778E-14	4,30296E-13	67426
Bcat1	24,06	0,61	9,55	0,44	-2,60	0	0	12035
Slc22a23	5,15	0,24	2,05	0,15	-2,61	0	0	73102
BC037704	1,25	0,24	0,49	0,12	-2,61	4,83361E-06	5,72194E-05	100502982
Gm9776	3,15	0,23	1,21	0,22	-2,62	3,40838E-14	1,02084E-12	328309
Hivep1	6,45	0,20	2,55	0,21	-2,62	0	0	110521
Snora65	2,55	1,43	0,94	0,40	-2,62	0,081695552	0,377513919	104367
1700030J22Rik	1,56	0,12	0,61	0,09	-2,63	2,45549E-11	5,53029E-10	69528
B930095G15Rik	2,41	0,16	0,95	0,18	-2,63	0	0	320268
Fam175a	11,39	0,85	4,42	0,39	-2,64	0	0	70681
Zhx2	6,85	0,36	2,68	0,17	-2,65	0	0	387609
Gm13217	2,86	0,32	1,06	0,49	-2,65	6,26704E-08	9,57314E-07	100417571
Rsc1a1	2,05	0,58	0,79	1,37	-2,66	0,074608594	0,351933859	69994
Ero1l	50,01	1,65	19,26	0,58	-2,67	0	0	50527
Mettl22	8,69	0,45	3,33	0,25	-2,68	0	0	239706
Tbc1d31	22,73	0,56	8,73	0,37	-2,69	0	0	210544
Lonrf1	4,28	0,12	1,63	0,13	-2,70	0	0	244421
Mertk	2,91	0,27	1,12	0,09	-2,71	0	0	17289
Npy1r	2,48	0,14	0,93	0,08	-2,71	0	0	18166
Zbtb10	5,37	0,52	2,02	0,23	-2,72	0	0	229055
Rnd1	27,32	2,53	10,35	0,63	-2,73	0	0	223881
Cnbd2	2,34	0,15	0,88	0,05	-2,73	0	0	70873
Osmr	9,29	0,49	3,50	0,39	-2,74	0	0	18414
Tars	195,06	9,66	73,30	4,93	-2,75	0	0	110960
Timp3	216,54	11,67	81,25	5,55	-2,75	0	0	21859
Rps13	1,20	1,07	0,43	0,75	-2,76	0,101835422	0,448512954	68052
Zbtb2	20,25	1,41	7,58	0,38	-2,76	0	0	381990
Recql4	21,82	2,33	8,17	0,63	-2,77	0	0	79456
Mtm1	6,20	0,53	2,28	0,16	-2,77	0	0	17772
Atf4	422,43	1,68	155,46	10,45	-2,81	0	0	11911
Zfp945	7,13	0,37	2,62	0,15	-2,81	0	0	240041
Mitf	1,36	0,06	0,50	0,07	-2,81	0	0	17342
Arhgef4	2,74	0,08	1,02	0,26	-2,82	1,11022E-16	3,99131E-15	226970
Alg12	10,40	0,09	3,76	0,24	-2,84	0	0	223774
LOC108167848	6,34	0,49	2,27	0,20	-2,85	0	0	108167848
Gm2446	9,91	1,61	3,63	0,71	-2,86	0	0	100039830
Ypel5	30,18	1,20	10,80	1,05	-2,87	0	0	383295
Dus4l	10,58	0,12	3,80	0,41	-2,87	0	0	71916
Serpina3g	1,32	0,32	0,47	0,07	-2,87	9,67287E-07	1,25637E-05	20715
Ifrd1	122,98	3,53	43,98	1,69	-2,88	0	0	15982
Them4	10,72	0,25	3,78	0,37	-2,89	0	0	75778
Arl14ep	41,33	1,90	14,60	1,29	-2,91	0	0	212772
Fosl1	37,89	3,42	13,50	1,25	-2,92	0	0	14283
Zfp202	3,16	0,28	1,13	0,20	-2,92	0	0	80902

Creld2	133,69	6,37	47,07	0,95	-2,93	0	0	76737
Jdp2	27,92	1,54	9,86	0,71	-2,94	0	0	81703
Fam71f1	1,78	0,37	0,61	0,08	-2,95	4,16558E-10	8,24916E-09	330277
Klf9	9,66	0,87	3,36	0,14	-2,95	0	0	16601
Plcxd2	3,13	0,29	1,09	0,04	-2,95	0	0	433022
Tmem154	2,81	0,28	0,99	0,18	-2,95	0	0	320782
Gm21092	13,64	0,56	4,77	0,10	-2,95	0	0	100861634
Rn7s1	12,56	13,49	4,39	0,66	-2,96	0,001251519	0,009866624	103948
Zfas1	54,12	3,37	18,80	0,46	-2,97	0	0	68949
Snhg15	14,71	0,44	5,10	0,17	-2,97	0	0	100041286
Gm4604	1,12	0,26	0,34	0,37	-2,99	0,009616966	0,061823356	100043718
LOC102640040	2,16	0,48	0,70	0,30	-2,99	0,000808307	0,006624673	102640040
Tma16	14,79	0,07	5,09	0,33	-2,99	0	0	66282
Dusp8	3,07	0,41	1,07	0,13	-2,99	0	0	18218
C920006O11Rik	1,22	0,06	0,42	0,08	-3,01	3,09333E-10	6,2036E-09	320295
Akna	6,83	0,64	2,32	0,16	-3,05	0	0	100182
Slc3a2	134,04	7,73	45,25	1,96	-3,06	0	0	17254
Grhl1	1,74	0,36	0,58	0,07	-3,07	2,55351E-14	7,74944E-13	195733
Rps29	1,23	0,67	0,40	0,69	-3,08	0,042938008	0,224321685	20090
Ccdc96	1,19	0,06	0,39	0,10	-3,08	1,30551E-12	3,33408E-11	66717
Gm18588	4,47	0,78	1,42	0,88	-3,10	8,97168E-07	1,1701E-05	100417394
N4bp2l1	8,10	1,34	2,67	0,24	-3,11	0	0	100637
Slc7a2	34,35	0,76	11,41	1,18	-3,11	0	0	11988
Kdm7a	4,05	0,28	1,33	0,16	-3,12	0	0	338523
Dennd4a	9,96	0,45	3,28	0,21	-3,13	0	0	102442
Megf10	2,26	0,28	0,75	0,04	-3,14	0	0	70417
Rad52	7,17	0,45	2,37	0,05	-3,14	0	0	19365
Hhipl1	4,35	0,41	1,44	0,11	-3,14	0	0	214305
Sdf2l1	80,53	10,89	26,33	3,43	-3,14	0	0	64136
Cyp1a1	3,55	0,63	1,16	0,10	-3,17	0	0	13076
Stk40	6,76	0,46	2,21	0,23	-3,17	0	0	74178
Slc25a33	18,22	1,01	5,92	0,25	-3,17	0	0	70556
Fhl4	2,99	0,52	0,96	0,11	-3,19	0	0	14202
Fam110c	26,07	2,35	8,43	0,89	-3,20	0	0	104943
Angptl6	16,46	1,33	5,27	0,27	-3,23	0	0	70726
Snhg1	54,46	2,00	17,32	0,80	-3,24	0	0	83673
Ndrg1	185,70	9,54	59,01	3,76	-3,25	0	0	17988
Aldh18a1	97,16	5,21	30,78	2,20	-3,26	0	0	56454
Lck	3,34	0,35	1,07	0,26	-3,28	0	0	16818
Gm14539	1,12	0,97	0,33	0,57	-3,29	0,073275063	0,347038522	100503459
Gpr85	6,38	0,15	1,98	0,21	-3,30	0	0	64450
Relb	29,19	2,34	9,11	0,48	-3,31	0	0	19698
Gm40363	1,13	0,09	0,36	0,13	-3,34	3,3645E-07	4,65467E-06	105244826
Pdzd7	1,65	0,16	0,52	0,10	-3,34	0	0	100503041
Irf5	1,53	0,20	0,48	0,14	-3,39	1,68754E-13	4,70916E-12	27056
Lins1	5,04	0,04	1,53	0,05	-3,40	0	0	72635

Ero1lb	7,21	0,68	2,18	0,06	-3,41	0	0	67475
Zfp967	4,01	0,96	1,20	1,24	-3,41	0,000745524	0,006151984	100303732
Pgk1-rs7	1,19	0,43	0,37	0,16	-3,42	7,98799E-06	9,14154E-05	668435
LOC108169077	1,67	0,40	0,51	0,08	-3,42	1,13867E-10	2,41222E-09	108169077
Hk2	9,43	0,24	2,85	0,09	-3,42	0	0	15277
Gm21811	274,75	7,48	82,71	3,00	-3,43	0	0	101056094
LOC106740	8,84	0,91	2,71	0,29	-3,43	0	0	106740
Amz1	5,18	0,53	1,52	0,20	-3,47	0	0	231842
Slc22a21	1,48	0,07	0,44	0,05	-3,47	0	0	56517
Vdac3-ps1	1,14	1,30	0,33	0,30	-3,47	0,010685161	0,067832763	22336
Rab39b	14,07	1,49	4,15	0,35	-3,48	0	0	67790
Dusp4	8,80	0,60	2,61	0,29	-3,48	0	0	319520
Unc5b	1,84	0,22	0,54	0,04	-3,52	0	0	107449
Cpox	32,50	1,47	9,37	0,19	-3,58	0	0	12892
Gm10031	1,06	0,99	0,31	0,36	-3,58	0,027719184	0,154873455	100039026
Spry1	1,15	0,29	0,33	0,10	-3,58	1,58338E-08	2,61412E-07	24063
Klf5	16,83	0,54	4,95	0,91	-3,59	0	0	12224
Zbed5	6,78	0,11	1,95	0,01	-3,60	0	0	71970
6820431F20Rik	1,69	0,15	0,49	0,10	-3,60	0	0	547150
Gm20652	1,04	0,13	0,30	0,11	-3,63	2,92094E-10	5,87849E-09	102636610
Slc6a9	88,70	5,27	25,20	2,53	-3,64	0	0	14664
Vegfa	26,35	1,02	7,49	0,39	-3,65	0	0	22339
Hspa5	1901,46	23,16	532,83	12,20	-3,68	0	0	14828
Wfs1	22,04	1,49	6,19	0,43	-3,69	0	0	22393
Hmox1	321,41	8,74	89,75	5,80	-3,70	0	0	15368
Casp4	11,41	1,24	3,13	0,22	-3,79	0	0	12363
Snhg17	12,33	0,36	3,36	0,37	-3,82	0	0	68108
Gm10557	349,52	9,76	94,27	4,12	-3,82	0	0	383374
Snhg12	34,21	1,09	9,12	1,14	-3,83	0	0	100039864
Creb5	1,65	0,11	0,44	0,06	-3,88	0	0	231991
5430416N02Rik	5,99	0,36	1,59	0,10	-3,90	0	0	100503199
G0s2	1,13	0,35	0,29	0,03	-3,93	0,000170306	0,001580306	14373
Gm6483	8,60	0,28	2,23	0,25	-3,95	0	0	624198
Gm10060	94,21	11,30	24,78	5,45	-3,96	0	0	100041567
1810010H24Rik	2,19	0,09	0,58	0,14	-3,97	0	0	69066
Mef2b	2,18	0,53	0,55	0,06	-3,99	1,43286E-09	2,66024E-08	17259
Gdf15	10,40	0,75	2,67	0,14	-4,01	0	0	23886
Gm42346	2,31	0,14	0,59	0,05	-4,03	0	0	105247207
Rgs16	2,75	0,44	0,70	0,09	-4,04	0	0	19734
Gch1	16,24	0,14	4,13	0,29	-4,06	0	0	14528
Otub2	8,42	0,31	2,10	0,18	-4,14	0	0	68149
Gm3365	1,69	0,03	0,42	0,04	-4,17	0	0	100041482
Arid5a	10,00	0,90	2,49	0,20	-4,18	0	0	214855
Tmem74	3,75	0,15	0,90	0,04	-4,25	0	0	239408
Agpat9	17,80	0,58	4,30	0,03	-4,27	0	0	231510
Tac1	1,04	0,15	0,23	0,11	-4,34	1,96156E-06	2,43876E-05	21333

Ciart	10,44	1,22	2,43	0,62	-4,39	0	0	229599
Tspyl4	10,01	0,91	2,34	0,10	-4,42	0	0	72480
LOC102636313	3,45	0,31	0,80	0,09	-4,44	0	0	102636313
Ets2	58,63	1,50	13,66	1,06	-4,44	0	0	23872
Socs2	4,53	0,08	1,05	0,07	-4,47	0	0	216233
Xbp1	88,34	0,62	20,21	1,52	-4,48	0	0	22433
Nupr1	332,28	22,19	74,89	5,09	-4,58	0	0	56312
Dnajb9	82,49	7,53	18,44	0,77	-4,61	0	0	27362
Arntl	18,51	1,36	4,12	0,14	-4,63	0	0	11865
Gm35816	1,71	0,22	0,37	0,04	-4,70	2,13869E-07	3,05389E-06	102639520
Gm19410	1,82	0,19	0,39	0,08	-4,74	0	0	100502846
Kif21b	3,09	0,13	0,67	0,04	-4,77	0	0	16565
Gadd45a	137,37	4,72	29,09	0,79	-4,87	0	0	13197
Mthfd2	115,95	3,96	24,57	1,87	-4,87	0	0	17768
Stbd1	23,61	1,03	4,94	0,15	-4,93	0	0	52331
Slc7a11	9,18	0,53	1,89	0,33	-5,01	0	0	26570
Ppp1r15a	86,93	4,92	17,93	0,20	-5,01	0	0	17872
Stard5	22,85	1,39	4,69	0,15	-5,05	0	0	170460
Slc1a4	37,56	0,62	7,70	0,58	-5,06	0	0	55963
Hoxa1	1,56	0,10	0,32	0,07	-5,07	1,11022E-16	3,99131E-15	15394
Gm20584	7,54	0,97	1,47	2,55	-5,10	0,000336879	0,002960873	100384905
Gm9923	1,81	0,72	0,36	0,32	-5,20	2,95747E-08	4,72586E-07	100040505
Dhrs9	1,65	0,14	0,34	0,15	-5,21	0	0	241452
Hoxb9	1,44	0,22	0,28	0,01	-5,23	2,10942E-15	6,98537E-14	15417
Gpt2	28,65	0,59	5,62	0,41	-5,28	0	0	108682
Paqr3	5,89	0,12	1,12	0,25	-5,36	0	0	231474
Snhg5	75,09	1,56	14,21	0,81	-5,44	0	0	72655
Nos2	2,74	0,33	0,53	0,09	-5,47	0	0	18126
Slc7a1	53,88	0,81	10,10	1,19	-5,52	0	0	11987
Acot2	21,37	1,53	3,93	0,26	-5,58	0	0	171210
Nfil3	11,71	0,44	2,10	0,30	-5,72	0	0	18030
Cth	35,22	1,04	6,40	0,57	-5,73	0	0	107869
Maats1	4,06	0,41	0,74	0,13	-5,75	0	0	320214
Rasgef1b	1,79	0,23	0,33	0,13	-5,94	0	0	320292
Gm20071	14,33	0,45	2,49	0,27	-5,96	0	0	100504116
Napb	7,63	0,20	1,31	0,25	-6,00	0	0	17957
Soat2	2,88	0,42	0,49	0,06	-6,05	0	0	223920
Neat1	47,24	3,33	8,07	0,58	-6,05	0	0	66961
Ptx3	48,34	1,94	7,72	0,99	-6,54	0	0	19288
Rpl12-ps1	1,27	1,10	0,17	0,30	-6,88	0,003011191	0,021877626	241053
Ccl20	2,04	0,38	0,30	0,03	-6,99	3,10366E-08	4,94048E-07	20297
Trim66	1,20	0,04	0,17	0,01	-7,31	0	0	330627
Gm13073	3,38	0,10	0,45	0,05	-7,59	0	0	105247217
Gm9299	1,22	1,19	0,16	0,27	-7,68	0,001486039	0,011532742	668675
1810032O08Rik	14,00	1,16	1,86	0,22	-7,87	0	0	66293
Areg	6,06	1,29	0,74	0,25	-8,19	0	0	11839

Extl1	1,97	0,17	0,26	0,11	-8,20	0	0	56219
Mmp13	17,42	1,60	2,14	0,17	-8,36	0	0	17386
Gm4521	1,48	0,74	0,17	0,30	-9,37	3,47004E-06	4,18794E-05	100043564
Herpud1	201,84	3,34	21,88	1,63	-9,56	0	0	64209
Rab11b-ps2	1,38	0,53	0,09	0,16	-13,44	2,55692E-10	5,19386E-09	665419
Fibin	2,31	0,16	0,17	0,05	-14,00	0	0	67606
Adm2	14,62	0,53	1,09	0,38	-14,06	0	0	223780
Ddit3	275,09	6,33	19,24	1,23	-14,83	0	0	13198
Slc30a1	5,09	0,74	0,35	0,03	-14,84	0	0	22782
Mir6363	1,02	0,91	0,00	0,00	-14,96	0,108901768	0,471111582	102466634
Trib3	145,01	7,80	10,02	0,91	-15,05	0	0	228775
Chac1	134,97	11,17	9,23	1,10	-15,14	0	0	69065
Atf3	49,32	1,63	2,97	0,48	-16,89	0	0	11910
Cass4	1,69	0,09	0,06	0,02	-26,20	0	0	320664
Hba-a1	2,49	4,21	0,08	0,14	-27,39	0,000215643	0,00196123	15122
Gm12396	1,63	1,44	0,00	0,00	-131,68	0,00385554	0,027297151	100042305
Hbb-bt	2,11	3,57	0,00	0,00	-168,84	0,003146831	0,022736777	101488143

Table S2. Primer design was performed using the ProbeFinder (version 2.53) provided on the website of Roche Life Science.

gene name	forward primer (5 ´- 3 ´)	reverse primer (5 ´- 3 ´)
Irs1	ctatgccagcatcagcttcc	ttgctgaggtcatttaggtcttc
Irs2	tgactataccgagatggccttt	gaggtgccacgataggttgt
Igf2r	ccttctctagtggattgtcaagtg	agggcgctcaagtcatactc
Igf1r	gagaatttccttcacaattccatc	cacttgcatgacgtctctcc
Insr1	tctttcttcaggaagctacatctg	tgtccaaggcataaaaagaatagtt
Insig1	cagatccagcggaatgtca	ggatacagtaaaccgacaacagc
Insig2	tctatgtccgttcttggttgc	tttttcagcaataactttgcattc
Socs1	tctgtctcccccatcagc	gcgtgctaccatcctactcg
Socs2	cgcgagctcagtcaaaca	agttccttctggagcctctttt
Socs3	atttcgcttcgggactagc	aacttgctgtgggtgaccat
Stat1	tgagatgtcccggatagtgg	cgccagagagaaattcgtgt
Stat2	ggaacagctggaacagtggt	gtagctgccgaaggtgga
Stat3	gttcctggcaccttggatt	caacgtggcatgtgactctt
Socs4	aatatgcagccgaagctctg	tgcgctgaatctcgaagtaa
Socs5	agcgggaagctgatctttta	cgttctcattacggcttcct
Socs6	tgtgagagtcatcaatttgttcaag	ataaactcaggacgccttgc
Stat5a	gagctggtgttccaggtga	ggtggcagtagcattgtgg
Stat5b	gaaacgagctggtctttcaagt	ctggctgccgtgaacaat
Rasa1	gagaaaaattactctatccagttgcac	ggctcgtacacgccttctat
Nras	ttgagacctcagccaagacc	tggcgtatctcccttaccag
Rra	tcacaagctggtggtcgtag	tgggatcatagtcagacacaaag
Hras	tgccatcaacaacaccaagt	ccattggcacatcatctgaa
Kras	tgtggatgagtatgaccctacg	ccctcattgcactgtactcct
Sos1	tcggcaactcactttacttgaa	tgtccacacacttccaactaattc
Sos2	gacctgcagtcgctctacatt	gaggcgtggaaggacataac
Araf	gaagacaagcccaagatgga	agctgccataggcctcct
Braf	agtcaccacagaaacctatcgtt	acaccacatcttgcgggta
Raf1	ccagagtgctgtgcagtgtt	tccaatctaagcgtgctttct
Srf	ctgacagcagtggggaaac	gctgggtgctgtctggat
Elk3	caccaagtggactgtttctgg	gttgagcagtgtggggaact
Elk4	actctcagcccgtttgctc	gggccatgactgttgagtaca
Elk1	gctccccacacataccttga	gggtgcaattggactcaga
Rab20	tgaagcagtggcgttcct	tacagggagcccagaccat
Rab7b	tcgaggaataccagaccacac	ctcctgaccacctgtgtcc
Rab21	tggtggaaaaagagtaaaccttg	cagtgcgtggaatctctcc
Rab5c	ggagaggagcggagtctgt	atgcaaagaggcacttaatgg
Rab15_l	aaggctgatgaagagcagaaa	tccatgccgtactccttagc
Rab39b_l	agatcgagccaggaaaacg	tgtagtaggcgcgagtgatg
Rab13_l	gatccgaaccgtggacatag	gcggtagttattgtcttgaatcg
Rab1a	tgggaaaacaatcaagctacag	tggaagtgattgttcgaaatctt
Rab24	gatgttcaggactatgccgataa	catccacactttggcctgt
Rab2a	tgggaaacagataaaactccaga	tgaccgtgtgatagaacgaaa
Rab5b	aagccagccctagcattgt	ctgcatatgcctgagcctct
Rabep1	gagcttttcccaagcaaaga	acctgttcacgtgactgcat
Rab34	ttgtgggttccaagaaggac	ggcatccttctccattaggg
Rab10os	cttggacaacagcacaccag	tgtataggagggtatcgcattca
Rab2b	actgcaaatctgggatacgg	cgtgatgtcatacaccagca

Tbc1d16	cgcagaagcgaaaggaatac	cctctggagtcatggagagc
Tbc1d9b	tacctgaccgtcaaccacct	acgtccacccactgtaccac
Tbc1d10a	gattgagcgtgacctgcac	ccttcagcacacggaaca
Tbc1d15	aagaaaccacacaccaatgga	caggaacgaccatttgcttt
Tbc1d7	gggaagctacctcgaagtcc	cgatggcaagaaagacttcat
Tbc1d4	cctctcagtttcccttaggattc	ccttgaatataacttcagttccttga
Tbc1d31	cagcaagatcaacgggaaat	ggcagcgatttcttgatctc
Tbc1d22a	tcgtccaatgacatggaaac	ggctggtcttcggtcaac
Tbc1d24	tgggtacagcctaagcaggt	cacagacctccttttgagtgg
Tbc1d23	cttctgccactgtcaacgtc	tcctgcttcaagggcttct
Rap2a	accagcagagcttccaagac	gttcccgaccaggatgact
Rap2c_l	ttgtacaacttgtgttgtccagtaaa	gtgcaaatttcctctgagtttgt
Rapgef1	gccaacaagcccagtgaa	tctgtcagagggagctcagg
Rap2b	aagtcaggctggtttaactgcta	agtgacatcactttacatccaacc
Rapgef3	cgacaccacaggttggaa	gagccaaacaggtgcattc
Rapgef2	aaagaaggtgccggtgaag	ttcaaagcttgtggagagtttatg
Arhgef4	tgaaaacatcgacaagattgct	gttccgagctcctgacca
Arhgef19	ccaggaggacaaggagatca	gtcctcacacactggacctg
Arhgef2	ctccggaggatcaagacg	ctcaacattcttctgtagcagctc
Arhgef39	aggaaggtcgccctgaat	gccaaagccacgacaaga
Rhou	cctacggccttcgacaact	actcatcctgtcctgcagtgt
Rhoa	gaatgacgagcacacgagac	tcctgtttgccatatctctgc
Rhob	cagactgcctgacatctgct	gtgcccacgctaattctcag
Rhoj	aacccagcctcttaccacaa	caaggttttggggtcatctc
Dlc1	cccctcatgacgaacaaact	gctggtccttgggaacatac
Sirt1	cagtgagaaaatgctggccta	ttggtggtacaaacaggtattga
Sirt3	tgctactcattcttgggacctc	gggcactgatttctgtactgc
Sirt4	tgatgtccaaaggctggaa	agagttggagcggcattg
Sirt6	gacctgatgctcgctgatg	ggtacccagggtgacagaca
Sirt2	tcaacacctcctgcagaaaa	ctcacacctgggagttgctt
Sirt7_l	tgtcatgcaactcctcatgaat	ggtcgccaaggagaagatt
Sirt5_l	ggccgagtttaacatggaga	ccgggaaaatgaaacctga
Creb1_l	ggaagagagaggtccgtctaatg	cacatattctttcttctttctacgaca
Crebrf_l	cggatccagtgcctgaac	Ctgcttgggtcaccactttt
Creb5	atcatgggcatgcaaggt	agcgcagccttcagtctc
Creb3l1	tccggagaaagatcaagaataaga	ctccaggctgtccatgtattc
Creb3	ccctcaacccttcctctca	cttgcggctttcttgagc
Creb3l2	tccggagaaagatcaagaataaga	ctccaggctgtccatgtattc
Crebl2	tccgagaggaactggaaatg	tgagctctgctgaggcttg
Crebzf	ccgcctcacaacctggta	ctaagcagccgagcatgag
Crebbp	acaagcgaaaccaacaaacc	cctgcaccaacagaaccaat
*Crtc1_*	tccgttccctgctctcac	aagagctgggggtgttcata
*Crtc3_*	ttgaccaacagcccatga	ggtggctctgctgtaccaa
*Crtc2_*	agagtctggctggcgaag	gcagacggcagtctaaacaa
Slc2a1_l	gaccctgcacctcattgg	gatgctcagataggacatccaag
Slc2a8_l	cagctgatggttgtcactgg	ccagcgccactctaggac
Slc2a6	gcgactcctggagagagaga	tgaaattgcccagcacag
Gnao1	ccgccaaagacgtgaaat	aagccatcttcatggatgatct
Gnaq	tcttgtggagtcagacaatgaga	ccaggggtaggtgataattgtt
Gna12	gataacttggaccggattgg	cttggtggcctttctagcc

Gnai2	tcaatgactcagccgcttac	gggatgtagtcactctgtgcaa
Gna11	cactggcatcatcgagtacc	gatccacttcctgcgctct
Gna13	ggtaccccagagtggtcttg	tctctgcagttgggaagttg
Gnai1	acgattcggcagcgtactat	tcctgctgagttgggatgta
Gnai3	ggagtccattaacaatctgttatcc	tcttcaaactggcactgaatgt
Adcy6	catcgcagactttgacgaga	aggtgctaccgatggtcttg
Adcy1	agatgggacttgacatgatcg	cgcatgttcaggtctacttcag
Adcy3	ggccttagagaagatgcaggta	aagctcagcatcatgacgaa
Leprot	tcttggaaatgtccctctacg	tgcctcatacccttcaggag
Leprotl1	tcacgttggacttctgtcgt	gccaacagctcagcatcac
Tbp	ggcggtttggctaggttt	tctgggttatcttcacacacca
Sdha	ccctgagcattgcagaatc	tcttctccagcatttgcctta
Rab3b	gtagccgaggtgggaacc	cagtcactgaagccatctcg
S100a10	gtcttcggcactagcctcat	ggcattttgaagagtctgtcg

Table S3. mHypoA-2/10 cells were cultured under low and high glucose concentrations and RNA levels determined by qRT-PCR with *Sdha* or *Tbp* as a reference gene. ΔΔCp using *Sdha* or *Tbp* were calculated and significant differences determined using two-way ANOVA and Sidak ́s post-test or two-samples t-tests.

qRT-PCR	*tbp*			*sdha*		
gene	change in %	ΔΔCp ANOVA Sidak ́s	ΔΔCp t-test	change in %	ΔΔCp ANOVA Sidak ́s	ΔΔCp t-test
Crtc2	88,35	***	*	103,6	***	*
S100a10	50,71	***	**	71,76	***	**
Irs1	41,24	***	*	52,09	***	*
Elk3	40,09	***	**	49,57	***	**
Rab3b	38,43	*	ns	45,51	*	ns
Insig1	34,21	***	**	43,05	***	**
Rap2a	27,19	***	**	44,85	***	**
Tbc1d16	31,16	***	ns	40,29	***	*
Rab20	32,55	ns	ns	38,47	ns	ns
Rap2b	23,75	***	ns	46,07	***	ns
Tbc1d4	28,97	***	**	37,39	***	**
Adcy6	27,55	**	****	37,01	**	**
Gnai2	24,03	**	****	38,97	***	****
Nras	24,54	***	*	32,19	***	*
Leprot	18,53	***	ns	35,49	***	ns
Igf1r	20,99	**	****	27,91	**	****
Rhoa	20,09	ns	****	28,16	ns	**
Gnai3	16,91	*	**	29,84	*	****
Gna11	15,07	*	****	29,48	*	**
Rab1a	22,48	ns	****	15,74	ns	***
Hras	15,32	*	***	22,11	*	**
Rab2a	18,83	ns	****	13,14	ns	*
Rab5b	17,35	ns	**	12,89	ns	ns
Gnao1	7,99	ns	*	21,82	ns	*
Rras	10,8	ns	*	16,39	ns	**
Adcy3	9,841	ns	ns	16,43	ns	ns
Crebl2	9,732	ns	ns	16,17	ns	ns
Crtc1	8,833	ns	ns	16,44	ns	ns
Arhgef19	5,515	ns	ns	13,85	ns	ns
Elk1	6,191	ns	ns	12,66	ns	**
Rab13	11,36	ns	**	6,008	ns	ns
Elk4	4,935	ns	*	11,42	ns	***
Rab5c	8,921	ns	ns	5,302	ns	ns
Sirt4	-0,5461	ns	ns	13,21	ns	ns
Rap2c	0,3793	ns	ns	10,58	ns	ns
Insig2	2,677	ns	ns	8,19	ns	ns
Gnaq	-0,4639	ns	ns	9,912	ns	*
Insr1	1,664	ns	ns	7,052	ns	ns
Kras	2,19	ns	ns	5,888	ns	ns

Rapgef2	-1,101	ns	ns	8,162	ns	*
Rhou	-0,00885	ns	ns	5,555	ns	ns
Creb1	-2,42	ns	ns	3,238	ns	ns
Rab7b	-0,907	ns	ns	1,076	ns	ns
Rab21	2,253	ns	ns	-3,395	ns	ns
Sirt2	-6,568	ns	*	5,105	ns	ns
Sirt5	-6,796	ns	ns	5,083	ns	ns
Tbc1d23	-4,091	ns	ns	1,955	ns	ns
Sos1	-3,471	ns	ns	1,318	ns	ns
Rab10os	-0,8372	ns	ns	-2,189	ns	ns
Arhgef39	-5,325	ns	ns	1,918	ns	ns
Gna12	-6,428	ns	ns	2,1	ns	ns
Creb3l1	-5,389	ns	ns	0,9837	ns	ns
Igf2r	-5,142	ns	*	-0,07038	ns	ns
Irs2	-5,253	ns	ns	-0,24	ns	ns
Rab2b	-5,904	ns	ns	0,2621	ns	ns
Crebbp	-6,28	ns	**	-0,7759	ns	ns
Gna13	-9,342	ns	ns	-0,2348	ns	ns
Creb3l2	-8,229	ns	ns	-2,097	ns	ns
Sos2	-7,372	ns	ns	-3,631	ns	ns
Srf	-9,88	ns	ns	-4,049	ns	ns
Gnai1	-11,89	ns	ns	-2,786	ns	ns
Sirt7	-12,92	ns	**	-2,433	ns	ns
Tbc1d10a	-10,58	ns	ns	-4,827	ns	ns
Tbc1d24	-11,78	ns	**	-6,348	ns	ns
Rab34	-8,13	ns	ns	-12,55	ns	*
Creb3	-13,52	ns	**	-7,969	ns	ns
Rapgef3	-16,36	ns	**	-5,89	ns	ns
Crtc3	-15,1	ns	**	-9,108	ns	ns
Tbc1d7	-14,64	ns	ns	-9,718	ns	ns
Rabep1	-10,03	ns	ns	-14,61	ns	ns
Tbc1d9b	-15,69	ns	*	-10,31	ns	ns
Rhob	-15,01	ns	*	-11,58	ns	*
Rasa1	-16,08	ns	*	-12,32	ns	**
Rhoj	-18,29	ns	*	-12,4	ns	ns
Tbc1d22a	-17,9	ns	****	-12,85	ns	***
Rab15	-16,17	ns	*	-18,65	ns	ns
Rab24	-17,19	ns	*	-21,5	ns	*
Sirt6	-24,1	**	**	-14,7	ns	ns
Crebrf	-23,83	ns	****	-19,32	ns	****
Tbc1d15	-24,07	ns	**	-19,8	ns	**
Sirt3	-26,98	***	**	-18,02	ns	*
Rapgef1	-27,69	ns	**	-17,84	ns	ns
Sirt1	-28,13	ns	***	-20,15	ns	***
Leprotl1	-28,8	ns	****	-20,07	ns	****
Dlc1	-27,2	ns	*	-26,16	ns	**

Slc2a1	-28,64	ns	**	-26,11	ns	***
Slc2a6	-30,75	ns	ns	-24,22	ns	ns
Creb5	-32,17	ns	**	-27,03	ns	*
Arhgef4	-33,22	ns	**	-28,91	ns	*
Adcy1	-32,41	ns	**	-30,79	ns	****
Crebzf	-34,65	ns	****	-30,51	ns	****
Rab39b	-41,25	ns	***	-42,54	ns	**
Arhgef2	-45,78	***	**	-46,81	***	***
Tbc1d31	-54,7	***	***	-52,56	***	***
